# Protein homeostasis imprinting across evolution

**DOI:** 10.1093/nargab/lqae014

**Published:** 2024-02-15

**Authors:** Thodoris Koutsandreas, Brice Felden, Eric Chevet, Aristotelis Chatziioannou

**Affiliations:** Center of Systems Biology, Biomedical Research Foundation of the Academy of Athens, Athens, Greece; e-NIOS Applications PC, Kallithea-Athens, Greece; University of Rennes, INSERM U1230, Rennes, France; INSERM U1242, University of Rennes, Rennes, France; Centre de Lutte Contre le Cancer Eugène Marquis, Rennes, France; Center of Systems Biology, Biomedical Research Foundation of the Academy of Athens, Athens, Greece; e-NIOS Applications PC, Kallithea-Athens, Greece

## Abstract

Protein homeostasis (a.k.a. proteostasis) is associated with the primary functions of life, and therefore with evolution. However, it is unclear how cellular proteostasis machines have evolved to adjust protein biogenesis needs to environmental constraints. Herein, we describe a novel computational approach, based on semantic network analysis, to evaluate proteostasis plasticity during evolution. We show that the molecular components of the proteostasis network (PN) are reliable metrics to deconvolute the life forms into Archaea, Bacteria and Eukarya and to assess the evolution rates among species. Semantic graphs were used as new criteria to evaluate PN complexity in 93 Eukarya, 250 Bacteria and 62 Archaea, thus representing a novel strategy for taxonomic classification, which provided information about species divergence. Kingdom-specific PN components were identified, suggesting that PN complexity may correlate with evolution. We found that the gains that occurred throughout PN evolution revealed a dichotomy within both the PN conserved modules and within kingdom-specific modules. Additionally, many of these components contribute to the evolutionary imprinting of other conserved mechanisms. Finally, the current study suggests a new way to exploit the genomic annotation of biomedical ontologies, deriving new knowledge from the semantic comparison of different biological systems.

## Introduction

Protein homeostasis (a.k.a. proteostasis) refers to a complex and interconnected network of processes that affects the ability of the cell to handle its protein needs. The molecular mechanisms controlling proteostasis are implicated in cell fitness, aging and contribute to disease onset. The underlying network of cellular mechanisms (i.e. the proteostasis network – PN) includes protein synthesis, co- and/or post-translational folding, quality control, degradation, as well as adaptive signaling in response to proteostasis imbalance (1). Moreover, proteostasis regulates a multitude of cellular processes and consequently has a powerful contribution to the large phenotypic diversity observed. As a result, the PN is a crucial biological module, with direct relevance to diseases linked with aberrant protein conformation.

From Prokaryotes to Eukaryotes, the PN is subjected to evolutionary pressure for each species, to cope with intrinsic and extrinsic demands. Evolution is shaped by factors such as genome complexity, the repertoire of post-translational modifications, the presence of subcellular compartments, the emergence of multicellularity and cell differentiation. Cells exhibit high protein synthesis yields and secretion due to all these factors, thus a robust PN is mandatory to provide homeostasis. An effective PN is also instrumental for eukaryotic cells with temporal variations of protein expression (e.g. neurons and endocrine cells). Each of these constraints increase the needs for updated, adaptive mechanisms, ensuring protein homeostasis (2). As such, the PN has been subjected to organism-specific selection. For instance, compartment-specific proteostasis control machineries in the cytosol, the endoplasmic reticulum (ER) or the mitochondria are required in eukaryotic cells, whereas dedicated systems are found in the cytosol and periplasm of Gram-negative Bacteria (3).

It is therefore evident that the PN undergoes important adaptations through evolution. Traditionally, the evolutionary relationships are determined through the comparative analysis of molecular sequences of conserved genes or proteins (4,5). Thus, the PN evolutionary profile could be investigated using proteins considered as its fundamental entities, as references. Heat shock proteins (HSPs) are such molecules because they exert important roles in maintaining protein homeostasis. Some HSP families (e.g. HSP40, HSP70) are highly represented in most cells and the nature of this representation might reflect the underlying evolutionary relationships - e.g. 3 HSP40 members in *Escherichia coli* (6) and 49 in *Homo sapiens* (7). While the output phylogenetic tree could probably depict correctly the evolutionary clades and ancestral lineages, as well as speciation events, which led to more complex HSP families and PNs, the content of amino acid sequences fails to thoroughly explain the evolution of the PN functional components. Therefore, a different strategy is necessary to obtain deeper insights for the evolution of PN structure. Studies have reported computational models of proteostasis in *E. coli* (8) and in Eukaryotes (9), however an overall layout of proteostasis evolution is still lacking.

A useful criterion to build such a layout could be the semantic description of PN-related gene sets, exploiting the available annotation of biological vocabularies. However, despite the essential role of proteostasis across species, its annotation is fragmented and problematic. As a result, the investigation and evaluation of its evolutionary imprinting is further hindered. Its large complexity is poorly documented by the available vocabularies, such as Gene Ontology (GO) (10,11), Reactome (12) and KEGG (13,14). Potential semantic terms that could thoroughly encapsulate and describe the biological content of the PN, like ‘proteostasis machinery’ or ‘proteostasis network’, are not included in any of these vocabularies. On the other hand, known functional components of the PN are described as descendant terms of other broad mechanisms. For instance, protein folding is described in the GO Biological Process (GO-BP) domain (GO:0006457) as a protein maturation and cellular process. Consequently, the genomic annotation of the PN is not standardized and it could be described only based on those of relevant sub-processes and the available literature.

Herein, we propose a novel phylogenetic analysis approach, which is based on semantic network analysis and describes the functional characteristics of species, under a specific cellular process (i.e. proteostasis). The core concept of this approach is the comparative analysis of semantic networks, as they are derived from the functional interpretation of pre-defined sets of biological modules (i.e. list of genes/proteins). We implemented this approach to evaluate the accuracy of the PN as an evolutionary marker and to identify its conserved and differential components. We created PN-related gene lists for hundreds of species (Archaea, Bacteria and Eukaryotes) and then we used the GO-BP annotation to translate them into semantic networks of biological processes. The derived networks were further analyzed to construct the PN-based phylogenetic clustergram and define the main components of PN. Proteostasis-based evolutionary map performed well vs. the gold-standard ribosomal RNA (rRNA) sequence-based evolution metric (15,16), while it outperformed those of HSP40 and HSP70 families. Hence, we show that the structure of PN represents a reliable metric for the partition of main taxonomic kingdoms, providing a snapshot of the overall, functional diversification of cellular functionality in the tree of life. Conserved and ‘kingdom-specific’ PN components were identified, implying that proteostasis is associated with a plethora of important cellular functions, in different taxonomies, apart from its core machinery. Additionally, we found that PN components are associated with the evolutionary imprinting of other conserved biological mechanisms of species.

## Materials and methods

### Data acquisition

#### Definition of the PN-related gene lists

**Figure 1.**
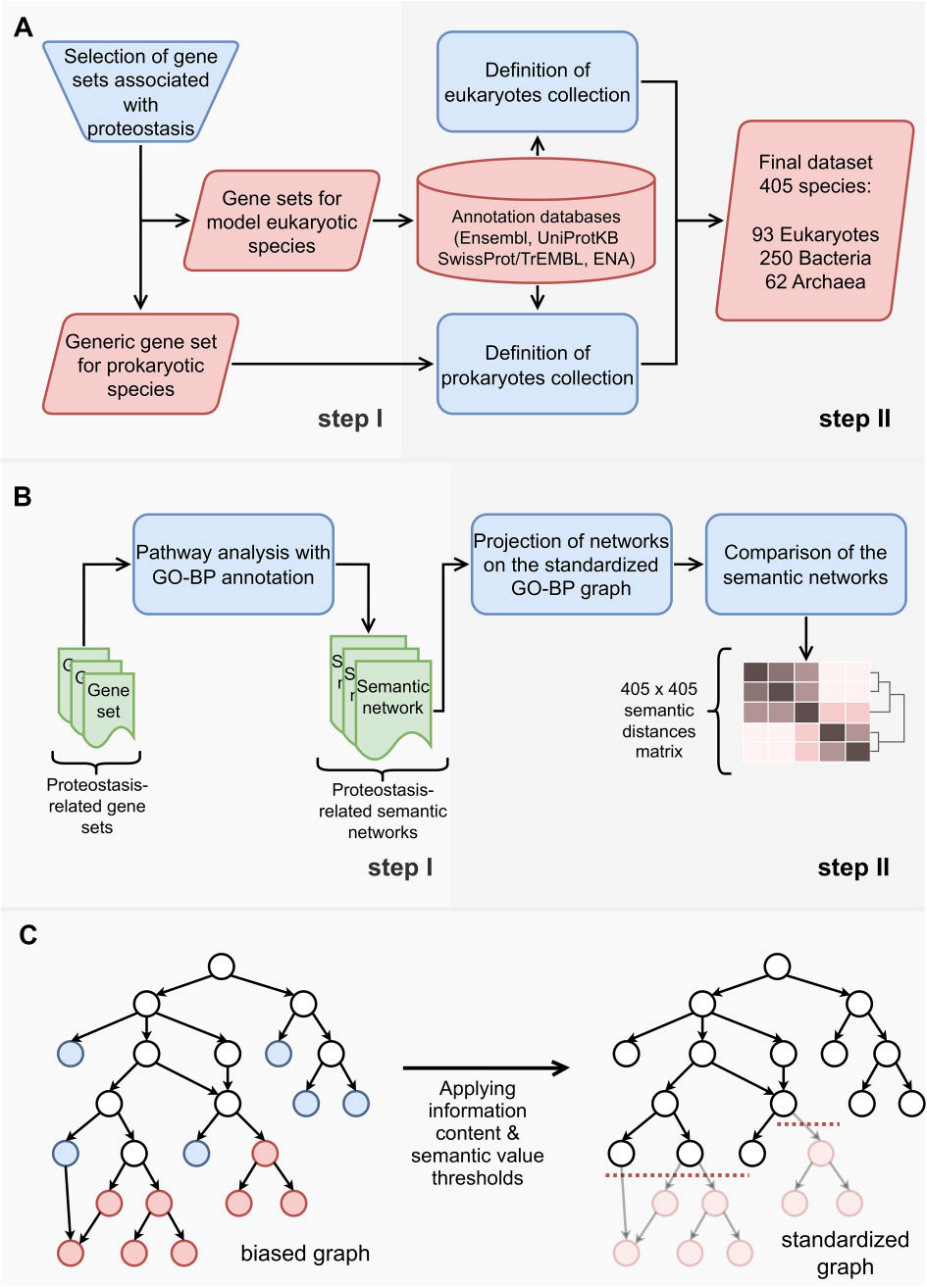
Schematic analytical workflow. (**A**) Process of data acquisition. Proteostasis-related gene lists were constructed manually for model species (step I). Then, public databases were used to collect genomic homologies to expand species collection and concentrate specific data for rRNA, HSP40 and HSP70 sequences. Only species with ample annotation were selected in the final set (step II). (**B**) Analysis workflow. Gene lists were translated into semantic networks through prioritized pathway analysis of GO-BP (step I). A standardized version of the GO-BP graph was constructed to remove potential annotation bias from the results. Finally, comparative analysis was performed to calculate the differences among the PN-related networks (step II). (**C**) Construction of the standardized GO-BP graph: Terms residing low in the branches of the graph bear high IC (red and blue nodes). Some of them are located in deep, densely populated branches (red nodes), reflecting the fact that these processes are more extensively studied than others. This imbalance in knowledge representation is a source of annotation bias, which was neutralized by filtering out terms in voluminous ontological regions (elongated and abundant branches), with high information content (red nodes).

As there is not any standardized genomic annotation for proteostasis, we used manual selection of genes strongly associated with the key functional components of proteostasis (Figure 1A, step I). In the case of Eukaryotes, this procedure was not feasible for all species with annotated genomes, because only a small part of them has been determined using human readable format (i.e. genes are named using nomenclature systems originated from the HGNC committee (17)). For example, inositol-requiring enzyme 1, a sensor of the Unfolded Protein Response in the Endoplasmic Reticulum, has been annotated in the Ensembl database (18), according to the automatic genome-wide determination of transcripts for all fungi (e.g. it is named MGG_15988 for *Magnaporthe grisea* and PAS_chr3_1104 for *Komagataella pastoris*), apart from *Saccharomyces cerevisiae*, for which it has been named IRE1. The genomic annotation of Prokaryotes in the UniprotKB (19) has been determined using a common and human readable nomenclature. Thus, we adopted a semi-supervised, multi-step workflow to define the PN-related gene lists and selected genes, which encode proteins related to protein folding, quality control and degradation, autophagy and relevant signaling pathways, and they are activated spatially in endoplasmic reticulum, cytosol or mitochondria. These seed gene lists were defined for eight Eukaryotes (*Homo sapiens, Gallus gallus, Danio rerio, Xenopus tropicalis, Caenorhabditis elegans, Drosophila melanogaster, Saccharomyces cerevisiae* and *Arabidopsis Thaliana*) and a generic gene list was created for the prokaryotic domain (see Data availability). For Eukaryotes, we applied homology mappings so as to retrieve putative, functionally similar genes, from the Ensembl database (vertebrates, fungi, metazoans and plants). The aforementioned model species were used to find homologies, with species belonging to the same taxonomic classification level (e.g. *S. cerevisiae* was used as a reference species for fungi and *A. thaliana* for plants). Hence, representative proteostasis-related gene lists were constructed automatically for hundreds of Eukaryotes. However, in order to exclude spurious annotations, only species pertaining to a number of genes, above 75% of the reference gene sets, were included into the final set. The prokaryotic gene list was used to construct a proteostasis profile for thousands of species. Data were extracted from UniprotKB, which contains numerous reference proteomes of fully sequenced species. To focus on taxonomically proximal species with ‘good quality’ genomic annotations, only species with complete proteome detector (CPD index equal to ‘Standard’) were selected. As thousands of Bacteria met that criterion, a random selection was then performed to reduce their number down to 250. The selection procedure adopted the constraint to select at least one member for each taxonomic Class of Bacteria with available data for the other phylogenetic criteria that were examined in this study (rRNAs, HSP40 and HSP70 sequences). The last constraint was applied also to Eukaryotes and Archaea to finalize the group of species that was used in the downstream analysis (Figure 1A, step II).

#### Data collection for rRNA and HSP sequences

After the creation of the PN-related gene lists for Eukaryotes and Prokaryotes, we collected data for ribosomal sequences (18S and 16S rRNAs), as well as specific heat shock protein families (HSP40 and HSP70). An exhaustive analysis of biological data public repositories was performed, to collect all these raw data. Specifically, FASTA format files for the rRNAs were manually collected from the European Nucleotide Archive (ENA) repository (20), while FASTA files for the HSP families were programmatically retrieved from both Ensembl and UniprotKB (Figure 1A, step II). Keywords like ‘interact’, ‘cooperate’, ‘stimulate’, ‘fragment’, ‘pseudogene’, ‘exchange factor’, etc. (see Data availability), were used to exclude sequences from these lists, which had been falsely retrieved as heat shock proteins. The PN-related genes sets and the retrieved data for rRNA and HSP sequences, for each species were included in the analysis, conforming with the following criteria: (i) genomic annotation in the GO-BP, (ii) availability of gene sequences rRNAs and (iii) at least one annotated amino acid sequence of HSP40 (*dnaJ*) and HSP70 (*dnaK*).

### Construction of the PN-related semantic networks

#### Pathway analysis of the PN-related gene lists

A common ontological vocabulary was needed, so as to functionally interpret the PN-related gene sets. For this scope, we used the GO-BP, applying prioritized pathway analysis to its corpus, translating each PN-related gene list into a set of GO-BP terms. Therefore, a PN semantic profile was constructed for each species (Figure 1B, step I). We used the prioritized pathway analysis described by Koutsandreas *et al.* (21), while the whole implementation is accessible through a publicly available, Galaxy-engine platform (http://www.biotranslator.gr:8080/). The algorithm utilizes the statistical distribution of enrichment scores, derived from the respective gene lists of each species, which is reordered according to the frequency of the scores, further corrected by non-parametric, permutation resampling, so as to prioritize the final set of enriched pathways. For each species, this semantic processing identified a network of biological processes, delineating the semantic tree of proteostasis. We used two criteria to determine the enriched biological processes. Hypergeometric *P*-value cut-off was set to 0.05 and then, the GO-BP terms were prioritized according to the adjusted *P*-value of the algorithm. The cut-off for the adjusted *P*-value was set at 0.05, however if a species had fewer than 100 terms satisfying that threshold, the selection was extended to the first hundred terms to keep a comparable cardinality, among the sizes of lists.

#### Construction of the standardized GO-BP graph

While PN profiles were obtained using the same vocabulary, their semantic resolution was different, because the depth of genomic GO-BP annotation differs among species. In general, different research communities have developed rich, genomic annotations for specific model species, emphasizing on specific components of cellular physiology (22). Alternatively, the vast majority of species has been sketchily annotated, only through annotations generated by electronic inference (assignation of putative functionality to the genes following homology-based annotations from in-silico testing that measures the level of conservation of the nucleotide sequences of each gene at an evolutionary scale) (23). This causes inconsistencies regarding the semantic network profile across species, and even taxonomically related species, could have divergence in terms of their annotation coverage (24). Additionally, inherent inconsistencies regarding the structure and the depth of GO-BP branches generate bias that tampers with the comparative analysis (22). Some graph branches are more expanded than others, due to extensive annotation. This leads to distorted, descriptive capacity, regarding the degree of semantic specification that each term bears.

Our approach aimed at establishing a standardized version of GO-BP, in order to neutralize annotation imbalances, which incurred bias in the description of PN among species. The construction of such a standardized, unbiased graph relies on two metrics of ontological graphs, information content (IC) and semantic value (SV). IC measures the specificity of a term, considering the amount of its descendant nodes (25). Conceptually, a term with plenty of descendant nodes has low IC value because its semantic content can be further broken down into more specific concepts. On the other hand, graph leaves have the maximum IC value. IC is defined as follows:


\begin{equation*}{{\rm IC}}_t = - {{\rm log}}_2\left( {\frac{{{D}_t + 1}}{N}} \right)\end{equation*}

where ${D}_t$ refers to the number of descendants of term *t* and $N$ is the cardinality of the complete set of terms. As it considers only the number of its child-terms, IC does not integrate the topological information of a term. For example, all leaf nodes have the same IC, but are located at different depths within the graph, depending on the quality of annotation. SV is a metric, proposed to overcome that limitation. It depicts the semantic distance of a term from the root, considering the information contained in its ancestral plexus (26):


\begin{equation*}{{\rm SV}}_t = \mathop \sum \limits_{a \in A}^{} \frac{1}{{1 + {\rm exp}\left( { - {\rm IC}_a^{ - 1}} \right)}}\end{equation*}

where $A$ is the set of ancestors of term *t*. The SV of a term is linearly correlated to the amount of its ancestors. High values point out either increased distances from the root, or the existence of extensively described semantic regions. The latter causes annotation bias favoring the overpopulated tree branches.

We constructed a standardized version of GO-BP, by filtering out very specific terms (high IC) from expanded ontological branches (high SV). Terms exceeding the twentieth (20^th^) percentiles of IC and SV distributions were trimmed and substituted with their most proximal ancestors, conforming to these thresholds (Figure 1C). The new, pruned graph was suitable to compare the GO-BP networks among species, by trading off between the detail of annotation of semantic branches and the need to minimize annotation bias.

#### Update of the PN-related semantic networks using the standardized GO-BP graph

The PN-related semantic networks were updated by substituting terms, not included in the standardized graph, with their appropriate ancestral terms. Terms included in the PN profiles, but not in the standardized graph, were mapped to ancestral terms that existed in the graph, using a recursive search to the upstream layers of the GO-BP hierarchy. Thus, each one of these terms was substituted with a list of less specific terms. For example, the term ‘peptidyl-proline modification’ (GO:0018208) was substituted with ‘protein metabolic process’ (GO:0019538) and ‘macromolecule modification’ (GO:0043412). On the other hand, each enriched term included in the standardized graph remained in the respective PN profiles (Figure 1B, step II).

### Comparative analysis of the PN-related semantic networks

The comparative analysis of the PN-related semantic profiles was performed by calculating the semantic similarities of GO-BP terms sets of species. The calculation of these group-wise semantic similarities of the PN profiles was based on the exploitation of the hierarchical structure of GO-BP. Initially, terms with IC lower than 0.1 were filtered out from the enriched sets. These terms referred to very generic biological processes (e.g. biological process, biological regulation, etc.) and their eventual inclusion in the PN profiles would suppress the potential semantic difference between two species. The rest of the updated PN profiles were used for the comparative analysis.

In general, semantic comparison estimates the closeness of two ontological terms, and is based on the topological relevance of their ancestors (pairwise measures). Due to the mapping of the enriched terms to sets of terms in the standardized graph, we adjusted the concept of semantic comparison to be applicable on groups of terms. Initially, a global similarity matrix was constructed for all the terms of standardized GO-BP. To avoid bias of specific pairwise measures, the similarity of two terms was calculated by averaging three widely used metrics: Resnik (27), XGraSM (28) and AggregateIC (26). Then, the similarity of two enriched terms was calculated as the mean similarity of the respective sets of terms in the standardized graph. In this way, we estimated the pairwise similarities of enriched terms. Finally, the similarity of two species was calculated based on the average best matches formula (28):


\begin{eqnarray*} &&SSim({{s}_1,{s}_2})\nonumber\\ &&=\frac{{\mathop \sum _{{t}_n \in G{O}_1}^N \left[ {SSim\left( {{t}_n,G{O}_2} \right)} \right]{\mathrm{\ }} + \mathop \sum _{{t}_m \in G{O}_2}^M \left[ {SSim\left( {{t}_m,G{O}_1} \right)} \right]{\mathrm{\ }}}}{{\left| N \right| + \left| M \right|}}\end{eqnarray*}

where $G{O}_1$and $G{O}_2$ are the enriched GO-BP sets for the compared species and *N*, *M* their cardinalities. Each sum function in the numerator refers to one of the two GO-BP sets and aggregates the maximum similarities of its terms with the other set of terms. The aggregation of best matches between these two lists is averaged by dividing it with the sum of their sizes. The final distance matrix was defined by subtracting similarity scores by one (Figure 1B, step II), and the phylogenetic clustergram was generated using Ward's minimum variance method (29).

### Comparison of the PN with other phylogenetic criteria

#### Construction of the rRNA and HSP-based phylogenies

Gene sequences of 16S and 18S rRNAs were used to construct the reference phylogenetic tree, as they traditionally portray the evolutionary proximities of species. The ClustalW tool (30) was used to quantify the pairwise distances and construct the final distance matrix, based on an *ad hoc* multiple sequence alignment (MSA). Furthermore, the amino acid sequences of HSP40 and HSP70 families were analyzed to examine their potential as surrogate, evolutionary markers. Consensus sequences for the HSP proteins were established, as heat shock proteins of the same molecular weight could vary significantly, even in the same organism. Each protein class consists of different members, which encompass certain, identical, functional domains, yet other additional components or their tertiary structure might be different. For instance, the human genome encodes 13 proteins of the HSP70 family and around 50 members of HSP40, which are divided in three main sub-families (7). Members of the same HSP family clustered to a consensus sequence pattern for each organism. Starting from the whole set of amino acid sequences, fragments were filtered out. The trimmed part fed the CD-HIT clustering algorithm (31) with similarity threshold to 95%. CD-HIT keeps the longest sequence, as a representative feature of each cluster, conserving as much information as possible for each one. If the output included more than one clusters, then an extra step was performed, by constructing their multiple sequence alignment (MSA) with ClustalW and the respective hidden Markov model (HMM) with the HMMER3 hmmbuild algorithm (32). The final consensus sequence was generated using the hmmemit function of HMMER3. ClustalW was used to calculate the distance matrices of consensus sequences, similar to the case of ribosomal sequences.

#### Representations of phylogenies in a two-dimensional space

As the aim was to compare the phylogenetic clustergrams and evaluate their discriminative power, as well as their efficiency to reproduce well-shaped taxonomic clusters, the examined species were projected on a two-dimensional space, based on their distances. Specifically, the distance matrix of each phylogenetic approach was transformed into a two-dimensional orthogonal space, using the Multidimensional Scaling (MDS) technique (33). MDS performs non-linear, dimensionality reduction, projecting the data on a new orthogonal space, where distances among samples converge to the initial values, under a relative tolerance of cost function. The generated scatter plots illustrate the adjacency of species groups, indicating the divergence of each criterion through evolution.

### Identification of proteostasis components

#### Clustering of the PN profiles

A two-steps clustering workflow was implemented to identify the PN-related GO-BP clusters (Supplementary Figure S1.1). The enriched GO-BP terms were first substituted by the respective sets of ancestral terms in the standardized graph. Then, the following workflow was applied to the three separate taxonomic kingdoms: (i) Terms, which were enriched in > 90% of species were identified. This list was filtered by removing terms whose descendants were also included in it, reducing the semantic redundancy. Hence, only uniquely defined terms remained in the list, namely the most semantically specific terms that were quasi-omnipresent in the examined PN semantic profiles. (ii) Terms enriched in less than 90% of species were screened towards their ancestral paths to reveal their ‘generic’ ancestors. As ‘generic terms’ were designated all those having two edges distance from the graph root. If a generic term was identified to be linked with enriched terms in more than the 50% of kingdom species, then it was considered as significant. While these terms were not included in the initial enriched sets, the accumulation of enriched terms in their semantic branches made them indirectly associated with the PN semantic profile of many species in the same kingdom. The union of the term sets, derived from these two clustering steps, constituted the definite set of PN-related GO-BP terms.

#### Construction and decomposition of the association matrix between GO-BP clusters and species

We quantified the association of species with the PN-related GO-BP clusters, using the pathway analysis results. For each PN-related term, the negative logarithm of its corresponding adjusted *P*-value or that of the minimum adjusted *P*-value of its descendant terms was kept, based on its inclusion or not in the results of pathway analysis. The calculated log score represented a reliable index of the enrichment of the given gene set in the PN of the examined species. Finally, we decomposed the constructed association matrix, using the method of non-negative matrix factorization (NMF) (34), in order to cluster the PN-related terms into three major evolutionary classes, and reveal the common and differential parts of proteostasis across the different taxonomies.

### Evaluation of the contribution of PN components to the classification performance of other evolutionary conserved mechanisms

#### Definition of gene lists for 20 biological processes

In order to evaluate the robustness of our semantic network analysis methodology and the contribution of PN components in the evolutionary profile of other mechanisms, we examined the semantic networks of other evolutionary conserved, biological mechanisms. We selected to analyze 20 conserved processes, as defined in the GO-BP graph, and both pathway and comparative analysis were performed based on their genomic annotation. Gene sets were retrieved from GO-BP for each species and biological mechanism. Using these gene sets in pathway analysis could lead to significantly biased results (very high enrichment scores and artificially small *P*-values for the selected GO term, as well as its ancestral path). In order to mitigate potential bias infiltration due to the annotated content and the size of a specific gene set, we introduced a randomization process to select the appropriate subset of genes of each pair of species and GO term (Supplementary Figure S1.2). Specifically, the metrics stemming from the depth of genomic annotation and the distribution of hypergeometric *P*-values of the respective PN-related pathway analysis, were adopted as criteria to assert the optimal gene set size for the biological mechanisms to be compared. If the annotation comprised less than 10 genes, then all of them were used as input for the pathway analysis, as this size is considered fairly small to generate critically biased results. For larger genomic sets, a random sampling procedure was implemented, to generate different lists of genes, which sizes were selected, so as to produce approximately similar, extreme hypergeometric *P*-values, to those observed during the analysis of the respective PN-related gene set. An iterative binary search was employed to estimate the maximum subset of GO term annotation necessary for the pathway analysis, to yield the lowest log-transformed hypergeometric *P*-value to the order of magnitude of the average of the 10 lowest log-transformed *P*-values of the respective PN-analysis. As this procedure is based on the random selection of gene sets, the final solution is erratic, namely the optimal size changes, based on the number and annotation of the selected genes. In order to render the selection process statistically robust, it was repeated 30 times, to create a distribution of optimal gene set sizes. Finally, different gene sets were generated, with size randomly selected from this distribution, or equal to 10, in case the distribution mean was less than 10. The adoption of all these criteria by the workflow, mitigated the creation of either completely biased or non-informative, semantic networks.

#### Pathway and comparative analysis of semantic networks to evaluate the contribution of PN components

Prioritized pathway analysis of these sets of genes was implemented to obtain a semantic network profile for each species. If random gene sets were generated for a species based on the above workflow, then only the GO-BP terms enriched in more than 20% of the outputs were considered as part of the final semantic profile. Comparative analysis was used to calculate the final semantic distance matrix and its phylogenetic clustergram. Each phylogenetic tree was divided in three clusters, aiming to evaluate whether the examined biological process could reproduce the three taxonomic domains. The accuracy to separate the main taxonomic domains was evaluated using the homogeneity (HS) (35) and silhouette (SS) (36) scores. Additionally, we examined the impact of PN components in the classification performance of these 20 mechanisms. Prioritized pathway comparative analyses were repeated, by opting out from the derived semantic networks those GO-BP terms, which were included in the calculated PN components. Therefore, the derived semantic networks were limited to the non-PN components of the examined biological processes. The comparative analysis was performed on these condensed profiles and the impact was measured using the HS and SS scores.

## Results

### Workflow implemented to construct the PN-related semantic networks

#### Data collection and selection of 405 species for phylogenetic analyses

The initial task in this study was to define PN-related gene lists for hundreds of species, to create the respective semantic networks and perform the PN-based phylogenetic comparison. However, the lack of standardized genomic annotation for proteostasis in public databases, led us to implement a semi-supervised workflow in order to infer these gene lists. The adopted workflow included both manual selection of genes and automated procedures. Our goal was not only to investigate the PN-related phylogeny, but also to compare its accuracy with that of rRNA and HSP families, so we sought to create and analyze a collection of species with available annotation for all these criteria. We generated comprehensive lists of rRNA, HSP40, HSP70 sequences and a gene set related to proteostasis for 405 species (93 Eukaryotes, 250 Bacteria and 62 Archaea; Figure 1A). These lists were then used for the phylogenetic analyses.

#### PN-related semantic networks are shaped by informative but not excessively specialized GO-BP terms

The PN-related gene lists were used to create the PN semantic networks. Specifically, prioritized pathway analysis on genomic annotation of GO-BP was applied to translate each gene set into a list of GO-BP terms, imprinting the PN-associated semantic networks (Figure 1B, step I, for gene lists and pathway analysis results see Data availability). On average, the initial PN networks contained 52, 78 and 100 GO-BP terms for Archaea, Bacteria and Eukaryotes, respectively. We then mitigated potential annotation biases in these networks, reducing the size of the graph to include informative but not very specialized terms. Thus, we created a standardized version of GO-BP, by excluding terms with very specific semantic meaning, located in expanded ontological branches. Following this reasoning, the initial set of 20596 annotated GO-BP terms was decreased to 2693 terms (Figure 1C). The PN-related semantic profiles were updated accordingly and their resulting average sizes were of 39, 63 and 76 for Archaea, Bacteria and Eukaryotes, respectively (e.g. the PN semantic network of *Halomarina oriensis* is depicted in Supplementary Figure S2.1). These PN-related semantic networks were then used to create PN-based phylogenies and compared to other phylogenetic-based annotations.

### Ribosomal RNA, HSP40, HSP70 and PN-based phylogenies

#### Proteostasis profiles separate efficiently the main taxonomic kingdoms

The construction of PN-based phylogeny was based on the comparative analysis of the PN profiles for 405 species (Figure 1B, step II). Different semantic operators were used to calculate a consensus similarity score for each pair of terms and then for each pair of species, averaging the similarities of term lists. The calculated semantic distance matrix was used to cluster the species in a hierarchical tree, forming the PN-based clustergram. The result revealed that the three main taxonomic kingdoms are separated efficiently (Supplementary Figure S2.3). The only misclassification was observed for a group of seven Bacteria (mainly *Planctomycetes*), which was embedded into the branch of archaea. The distribution of semantic intra-distances of the three taxonomic kingdoms pointed out that the variance of the PN profiles increases from Archaea to Bacteria and peaks in Eukaryotes (Supplementary Figure S2.7). This signifies the higher complexity of the PN in Eukaryotes compared to Prokaryotes. Moreover, inter-distances distribution indicates that Archaea and Bacteria share more similar networks than with Eukaryotes. This likely reflects the heterogeneity of PN content, bisected into taxonomy-specific components and others that are conserved across evolution.

#### PN- and rRNA-based classifications outperform those based on HSP families.

**Figure 2.**
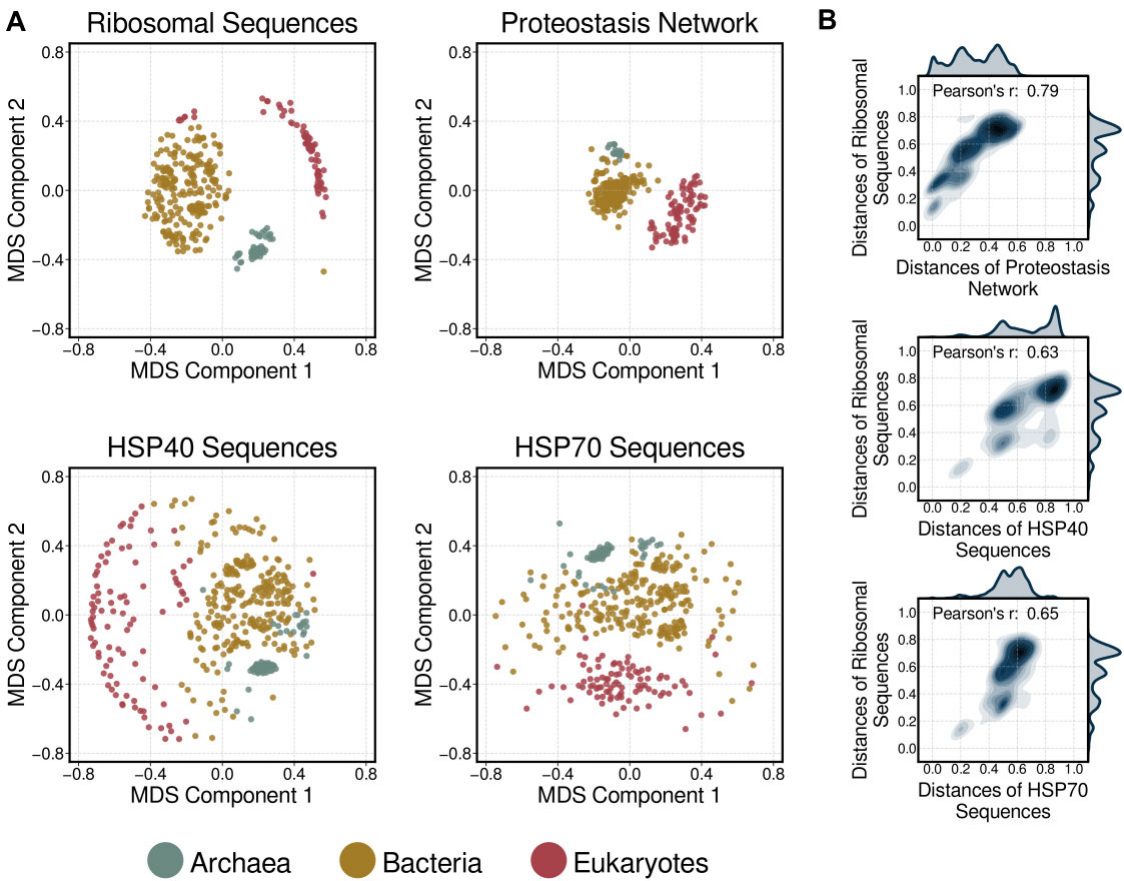
Establishment of PN-based phylogenetic trees. (**A**) Two-dimensional representation of species in function with their taxonomy. The derived evolutionary distance matrix of each criterion (rRNA, proteostasis and HSPs), was transformed into a two-dimensional orthogonal space through the Multidimensional Scaling (MDS) algorithm. Similarity distances of each organism from the centroid of the single class problem are projected in those exploratory scatter plots. (**B**) Pearson correlation of pairwise distances of rRNA sequences with the other three measures. Correlation is unbiased from taxonomic domain sizes, as we used a maximum of 80 randomly selected species from each domain for the calculation.

To evaluate the efficiency of proteostasis as an evolutionary marker, we compared the structure of PN-based phylogeny to those produced using sequence-based criteria. We created the phylogenetic clustergrams for rRNA nucleotide sequences, HSP40, HSP70 amino-acid sequences (Supplementary Figure S2.2-5) by applying the same agglomerative clustering used with PN semantic networks. To further explore the characteristics of each evolutionary tree, species were projected on a two-dimensional space (Figure 2A). Overall, rRNA sequences produced nearly independent sub-groups for each established taxonomic domain, similarly to the PN semantic profiles. The only inconsistency of rRNA-based phylogenetic clustergram with the reference was observed with a group of *Streptophyta*, which clustered close to Bacterial species (Supplementary Figure S2.2). PN evolution appeared more constrained than that of rRNA, which led to distantly separated kingdoms. The distributions of PN- and rRNA-based distances corroborated this finding (Supplementary Figure S2.6-7), as the PN semantic profiles produced systematically lower inter- and intra-distances among the taxonomic domains. Nevertheless, the pairwise distances among species for rRNA and PN showed high correlation (Figure 2B). Concerning the HSP-derived classification, a poor correlation of the HSP40 and HSP70 sequences with evolution was observed, as they only succeeded to separate eukaryotic and prokaryotic kingdoms, even so not flawlessly. A weaker but not neglectable correlation with rRNA sequence-based distances suggests that the intra-distances of taxonomic clusters follow approximately the same distribution to their inter-distances (Supplementary Figure S2.8-9). These observations imply that HSP-based classifications lack informative power as ‘isolated’ markers of species evolution.

#### The accuracy of proteostasis as evolutionary marker declines in lower taxonomic clades

We next sought to compare the accuracy of PN, as well as rRNA and HSPs sequences to classify species of the same taxonomic domain. We examined the class-level categorization of Archaea and that of phylum-level for Eukarya and Bacteria. For each criterion, different species clustering models, for a range of predefined number of clusters, were generated and the consistency of each model with the reference classification was assessed, using the homogeneity score. The output corroborated the findings inferred from the rRNA sequences and revealed an overall homogeneity of PN profiles in Bacteria and Archaea (Supplementary Figure S2.10). None of the criteria succeeded to absolutely reproduce the number of the reference taxonomic groups, indicating that species of different lower-level taxonomies share similar profiles. Regarding the PN-based clustering models, the low homogeneity scores for the prokaryotic domains demonstrate high conservation of PN topology, regardless of the lower-level taxonomic classifications. In contrast, the accuracy of PN-based models was significantly improved for Eukaryotes, suggesting that a key avenue of their evolutionary adaptation resides in the diversification of the plasticity of their genetic circuitry, enabling novel, emergent cellular functions (see the PN-components analysis below). Hence, the PN comprises variations that coherently segregate eukaryotic species, rescuing phyla segregations to a large extent. HSP-derived clusters were identical for all the examined numbers of clusters in Prokaryotes, while they showed high similarity to those obtained with rRNA sequences. However, they declined among Eukaryotes, probably due to the high variation of protein families in species-specific profiles. The efficiency of PN as a taxonomic marker in the lower evolutionary taxonomies is inverse to that of HSP sequences, implying that the profile of proteostasis is more informative for complex organisms, while lower species have significantly similar PN topologies.

### Proteostasis as a functional metric to trace evolution

#### The PN encompasses both conserved and taxonomy-specific components

**Figure 3.**
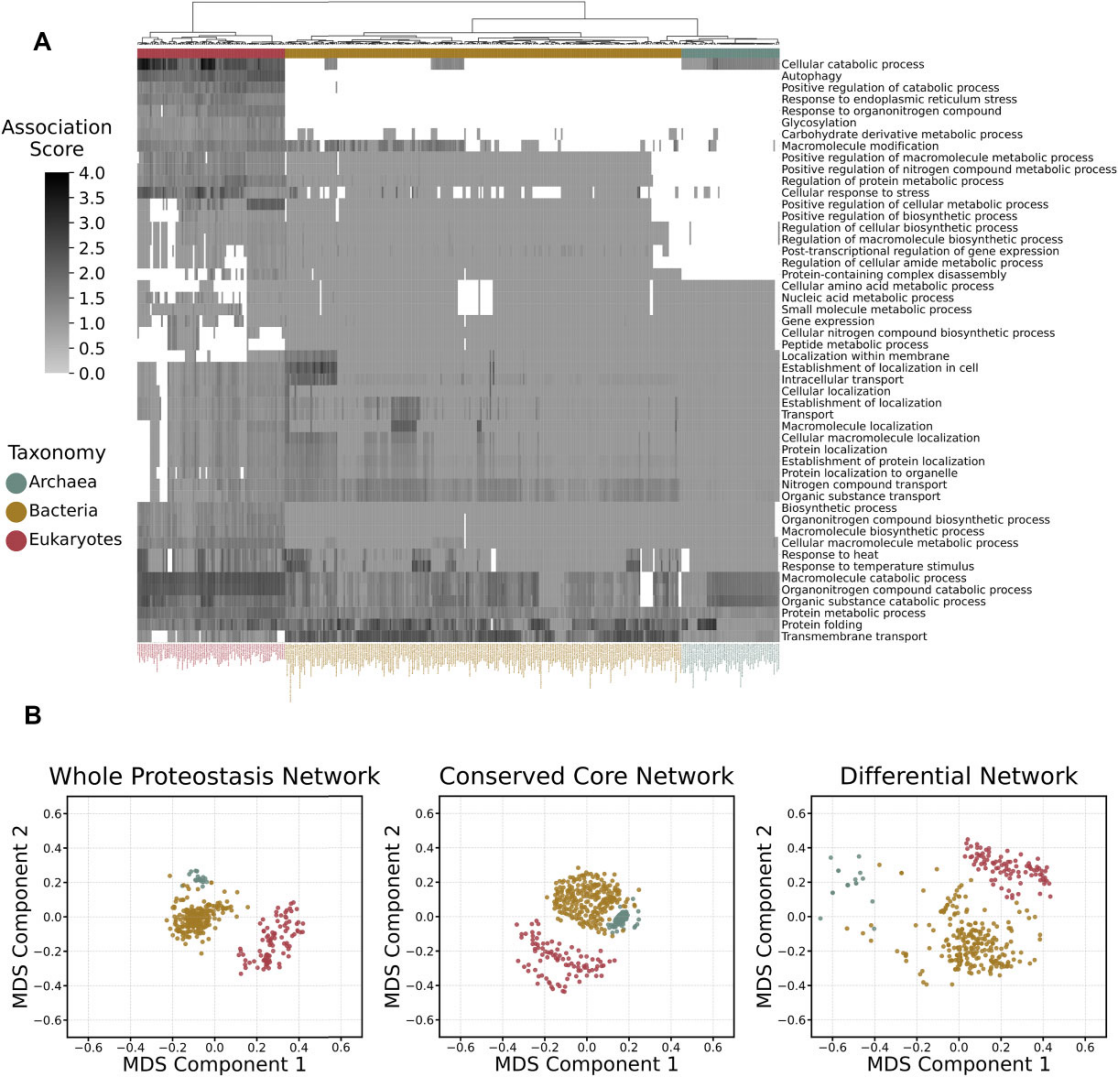
PN composition evolution throughout various phyla. (**A**) The association matrix of proteostasis-related semantic groups with the examined species. The score of each semantic group corresponds to the average negative log-transformed adjusted *P*-value of the GO-BP terms, which have been clustered into this group. (**B**) Two-dimensional representation of species based on their proteostasis semantic profiles. Conserved and differential components were used separately to investigate their contribution to taxonomic domain separation.

The semantic comparison of PN-profiles indicated that they include adequate biological information to distinguish the main taxonomic domains and partially the groups of eukaryotic Phyla. We thus explored the components of PN which underlie this classification efficiency. To delineate the content of the PN profiles and explore how they change across the three taxonomic kingdoms, we applied a clustering workflow starting from the results of pathway analysis and ending up to 50 semantic term groups. The adjusted *P*-values from pathway analysis were used to quantify the association of the examined species with these groups (Figure 3A). Then, the NMF-based decomposition was applied on the association matrix to transform it into a matrix of three components (Supplementary Figure S2.11), and another one which indicates the association of species with these components, showing noticeable kingdom-specificity (Supplementary Figure S2.12). This transformation also revealed the conserved, as well the differential parts of PN-profiles across the three kingdoms. Metabolic processes, transport- and localization-related processes, protein folding and cellular responses, especially due to temperature stimulus, were enriched in all tested species, thereby representing the PN ‘conserved core’. Alternatively, regulatory mechanisms, signaling pathways and mechanisms related to cell death constitute the differential PN component, which distinguish Bacteria from Archaea and Eukaryotes for Prokaryotes. The two-dimensional representations of species based on the whole PN (Figure 3B, left), the conserved PN (Figure 3B, middle) and the differential PN (Figure 3B, right) produced independent sub-groups for each taxonomic domain. These sub-groups increased their density when analyzing the PN ‘conserved core’ (Figure 3B, middle) and were sparser when analyzing the differential PN (Figure 3B, right). In the case of Archaea, the great sparsity in the differential PN reflects maximum semantic distances among species, which have been caused by the removal of the PN ‘conserved core’ and the subsequent elimination of their semantic profiles. These data unveil conservation and divergence within the PN, highlighting circuits of ubiquitous functions and emerging mechanisms indicative of each taxonomic domain.

#### The conserved PN profile

The obtained GO-BP term groups presented significant semantic relevance or even overlap. For example, the ‘conserved core’ included many terms, which correspond to catabolic processes. All these generic terms were identified because different components of catabolism were enriched for all the PN-related gene sets. Protein catabolism is one of these components. However, the identification of ‘protein metabolic process’ in the ‘conserved core’, signifies that additional processes related to the modification of proteins are found in the PN semantic profiles (such as ‘protein maturation’, ‘formation of translation preinitiation complex’, ‘protein peptidyl-prolyl isomerization’ and ‘histidyl-tRNA aminoacylation’), apart from ‘protein catabolic process’. Two other biological processes, highly represented in the ‘conserved core’, correspond to localization and transport. Terms related to response to stress designate another distinct set of processes. ‘Response to temperature stimulus’ is the semantic parent of ‘response to heat’, so their co-existence in the ‘conserved core’ produces semantic redundancy. This is likely due to genomic annotation resolution imbalances, across species of different taxonomies. Particularly, ‘response to heat’ is found as a quasi-omnipresent term in Archaea, while ‘response to temperature stimulus’ is detected in the PN profiles of Bacteria and Eukaryotes. Both of them imply the association of proteostasis with components of cellular response to abiotic stimuli.

#### The differential PN profile

The ‘conserved core’ includes the main PN-profile of Archaea (component 3). This profile lacks regulatory mechanisms, which have been assigned to that of Bacteria (component 1) and Eukaryotes (component 2). While prior knowledge about the regulatory processes in Archaea exists (in the genomic annotation of GO-BP), this finding implies that they may not have a strong contribution to PN regulation. For instance, the concept of ‘post-transcriptional regulation of gene expression’ exists in both Archaea and Bacteria. Its genomic annotation in Archaea contains translation initiation (*aif5A, tef5A, tif5A, eif2g, eif5a*) and elongation factors (*efp*), synthases (*dph2, dph5, dph6, dphB*), reductases (*cbiJ*) and ribosomal proteins (*rpl1, rpl13, rpl1P, rpl1p, rplA, rps4*), which participate in the process of protein biosynthesis. Alternatively, the same regulatory process is well documented in Bacteria, containing genes, which regulate translation and consequently could be considered as molecular entities of proteostasis apparatus, such as small heat shock proteins (*ibpA*), ribosome hibernation factors (*hpf*), elongation factors that act under stress conditions (*lepA*) and proteins, which monitor the translation of Sec system components (*secM*). The expression of such regulatory entities consequently forms a more complex PN semantic profile for Bacteria than for Archaea. In addition, specific processes related to programmed cell death and signaling pathways were enriched in Eukaryotes. Enrichment in terms associated with the response to endoplasmic reticulum (ER) homeostasis imbalance was effective in all examined eukaryotic species, as expected, pointing out this compartment as a hotspot for proteostasis. Regarding the process of autophagy, many descendant terms identified exclusively in Eukaryotes, related to either associated membranous structures (‘autophagosome assembly’) or subprocesses and mechanisms (‘lysosomal microautophagy’, ‘autophagy of nucleus’, ‘chaperone-mediated autophagy’). The extended PN profiles of Eukaryotes were further enriched by the integration of protein glycosylation and additional regulatory and cellular response mechanisms.

### Impact of PN evolution on other functional networks

#### Use of other conserved mechanisms as evolutionary markers

**Figure 4.**
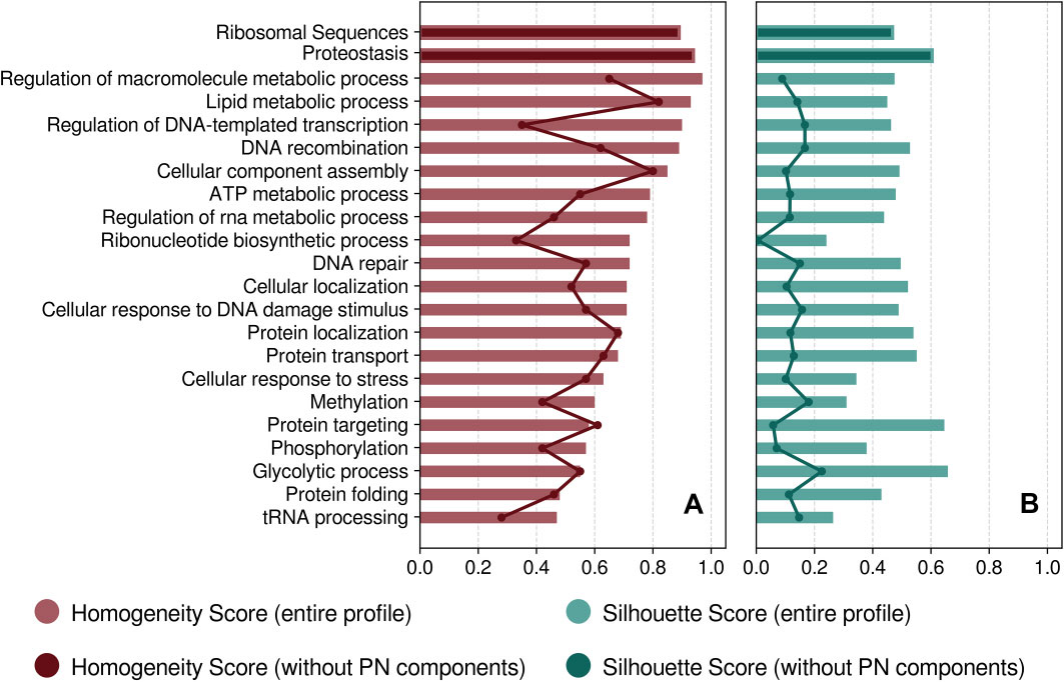
PN contribution to other evolutionary conserved mechanisms. Homogeneity score (**A, red**) refers to the separating ability of each biological process regarding the three main taxonomic domains, through the respective semantic network, derived from the pathway analysis of related genes. Silhouette score (**B, green**) indicates the degree of cohesion of cluster inference, by measuring the trade-off between intra- and inter-distances of each cluster member. Bars display these scores for the entire machinery of each process whereas the solid lines illustrate the same scores calculated after the removal of proteostasis related components from the semantic profile of each process.

At last, we examined the robustness of the proposed approach, as well the contribution of PN components to the evolutionary imprinting of other biological mechanisms, by running the comparative analysis for 20 GO-BP terms. Phylogenetic analysis of these conserved processes showed that those, which emerge as gains of functions during species evolution, or those, which evolved through higher functional complexity, segregate sufficiently the three kingdoms (Figure 4; S2.13A–S2.32A). The identified biological mechanisms are related to cell compartmentalization, regulatory networks, lipid metabolism and DNA recombination. Some performed marginally better in terms of accuracy compared to proteostasis or to rRNA sequences - *i.e*. slightly higher HS values due to the erroneously classified Bacteria in the case of PN, and the group of plants in the case of rRNA sequences. Nevertheless, these biological processes phylogenies exhibited lower SS values than those obtained for PN (Figure 4B), which means that the produced clusters were sparser (especially within Prokaryotes) and consequently, the phylogenetic trees contained broader clades. In addition, parts of the aforementioned mechanisms or those with narrower functional networks showed weaker performance with clustering efficiency, especially due to their strong commonalities among the Prokaryotes. HS measurements were below 0.8, because many Archaeal species were classified in Bacteria, and *vice versa*.

#### PN components impact the performance of other mechanisms as evolutionary markers

The description of PN components in the three kingdoms verified the strong functional association of proteostasis with a multitude of cellular processes. To further examine this hypothesis, we excluded the PN components from the semantic profiles of the analyzed mechanisms, repeating the comparative analysis and the construction of the respective phylogenetic trees (Figure 4; S2.13B–S2.32B). The overall classification performance of the majority of mechanisms then decreased significantly. Specifically, only a few processes (‘cellular component assembly’ and ‘lipid metabolism’) retained adequate information to distinguish accurately the taxonomic domains, implying that their mechanistic frameworks can differentiate the taxonomic kingdoms. In general, all processes with accurate performance suffered from low silhouette scores, and some lost their phylogenetic congruity. This analysis indicates that a significant part of the semantic description and components of PN is included in other mechanisms related to cell homeostasis and functions and partially represent their evolutionary divergence in the main taxonomic groups.

## Discussion

### PN-based classification: a novel approach for phylogenetic analysis

Taxonomies based on molecular sequences have increased our understanding of evolutionary processes. Phylogenies based on isolated macromolecules conserved among evolution, such as nucleic (DNA/RNA) or protein sequences, have their limits as they do not accurately represent the complexity of life evolution. Herein, we designed and applied a novel approach for phylogenetic comparison, which uses the semantic network graph as a new metric, to evaluate the complexity of protein homeostasis by monitoring the proteostasis network (PN). To the best of our knowledge, it is the first time that semantic networks analysis is used to examine a distinct, biological process as an evolutionary marker. Regardless of the ability of the PN topology to indicate the main taxonomic characteristics of a species, the proposed approach stands as a novel strategy for taxonomic classification, which assess the divergence of the semantic topology, providing species-specific PN profiles rather than analyzing individual sequences. This new method relies on the comparison of the enriched semantic graphs inferred by a pathway analysis tool and suggests that the semantic interpretation of gene/protein sets for different species could provide biological insights about the impacts of the evolutionary pressure, and the extent of conservation of mechanisms among different taxonomic domains.

### Current limitations of the approach regarding the genomic annotation

GO-BP as the basic framework for this study was used as it constitutes a unified description for all the known biological processes in living species (10). All well-established semantic operators (25,37) were developed to be applied on ontological graphs (directed acyclic graphs), in which the content of graph root is explained gradually, using parent-child relations in a top-down direction. The concept of common ancestors or that of the most informative common ancestor (38), which are used by many of these operators to estimate the similarity between two semantic terms, entails the distribution of terms in such a hierarchical structure and not simple vocabularies. Currently, there is not any other controlled vocabulary that describes the genomic universe of cellular functionality in the level of pathways or processes for this breadth of species, in a hierarchical graph. For instance, the BioCyc database (39) provides annotation for thousands of species but only for metabolic pathways, Reactome Pathways (12) includes a broader hierarchical functional annotation for molecular pathways, but only for a few species and the mappings of KEGG database (13,14) are available for thousands organisms but they are not structured hierarchically, and thus they do not allow the implementation of deductive logic strategies. Nevertheless, shortcomings of the proposed approach to adequately quantify potential nuances in the prokaryotic semantic topologies, as well as the need to apply the semantic comparison on the standardized GO-BP graph, portray the current limitations. A possible way to overcome this could be the massive integration of different vocabularies for the construction of a unified ontological scheme, like a hyper-ontology, with increased descriptive resolution. For example, the metabolic and signaling pathways described extensively in the KEGG database could be connected with related GO-BP terms in a hyper-graph, which would be able to describe not only the biological processes in cells, but also the underlying topology of molecular interactions. Moreover, the annotation bias that compelled the usage of the standardized graph for the evolutionary analysis could be attenuated with the continuous improvement of proteins and genes annotation. This is an ongoing effort (40) and future advancements could provide a more complete and in-depth functional annotation for the universe of species.

### The diversity of proteostasis along species evolution

In this study, we aimed at illustrating the complexity of the PN modular architecture, based on the proposed phylogenetic analysis approach. This analysis measured species-specific topological differences, and translated them into evolutionary branches. We show that monitoring PN characteristics provides reliable information about the evolution of living organisms in the level of kingdoms, whereas monitoring its individual components has limited interpretation. PN succeeded to separate the three main taxonomic domains almost infallibly, performing as accurately as rRNA sequences do. Moreover, PN performed much better than isolated PN components (e.g. conserved HSP families). This primarily indicates that this new method can disclose evolutionary differences among species of different taxa. It also demonstrates the utility of PN as a reliable evolutionary marker, able to classify species according to their main taxonomy, contrary to the limitations of the sequence-based criteria. The efficiency of the PN metric to increase taxonomic resolution dropped in Bacteria and Archaea, which suggests that their evolutionary pressure is more intensely dictated by the functional diversification of selected genes (e.g. mutagenesis) or chromosome variations. In Eukaryotes, the complexity and adaptability of molecular circuitries became a major driving force. For Eukaryotes, the evolution of compartmentalization impinges upon the complexity of protein circuitry, prompting evolution of the PN to cope with these additional constraints (3). Deconvolution of the PN in various molecular sets revealed that many, if not all, cellular processes are connected to the PN, especially in Eukaryotes. The conserved PN component across the vast majority of species is linked with protein production, folding and degradation. It is also linked with responses to external or internal stimuli, activation or repression of anabolic and catabolic processes, to maintain cell homeostasis, as well as the proper localization of macromolecules. This comprises heat shock proteins, which perform poorly in terms of separating the taxonomic super-kingdoms, but were recently shown to organize beyond the ‘de novo stress-inducible’ scheme, into a layered core-variable architecture in multi-cellular organisms (41). To cope with proteome complexity that arises with evolution, conserved core chaperones increased in abundance and new co-chaperone families appeared (42). Other functional modules, considered as ‘gain- or loss-’ of adaptive functions, were also found to be connected to the PN. This, in turn, could help identify the role of PN in maintaining those functional changes. The instrumental role of proteostasis as a robust indicator of cellular and organismal adaptation to evolutionary cues, is highlighted by the taxonomic underperformance that the other mechanisms exhibit when PN components are excluded. This provides evidence for a tight coupling between proteostasis and other major biological processes. As such, the architecture of the PN encapsulates critical biological information to categorize species according to their complexity and acts as a clear-cut fingerprint of evolution, as it has co-evolved with the cell proteome and provided a driving force for adaptation to favor emergence of new traits.

### Future directions

To conclude, the implementation of the proposed approach is not limited to the comparison of semantic networks to delineate the evolutionary maps of biological mechanisms. In a previous study (43) we used the genomic annotation of different ontological vocabularies to analyze and compare the imprinting of 12 host–viral interactomes, demonstrating that the human-SARS-CoV-2 interaction network share the highest functional similarities with those of enteroviruses (*Rhinovirus C15* and *Coxsackievirus A10*). Therefore, semantic analysis could also be used to measure and compare quantitatively the functional background of pathological states, diseases or phenotypes, elucidating their differentiated components and revealing hidden commonalities. A more specific scenario could be the implementation of semantic analysis to study the diversity of the same molecular mechanism, such as that of proteostasis, in various pathological conditions or to categorize diseases and possibly their treatments. Approaches relying on the analysis of PN sub-networks (e.g. the ‘chaperome’) were proposed to be effective in various pathologies, such as cancers (44), degenerative diseases (45) or in cell differentiation (46). An interesting approach could also rely on the whole PN to assess how its deregulation could mark disease appearance and progression, or even propose specific nodes as potential therapeutic targets. Finally, all these scenarios indicate that the current study paves the way in the development of a new class of computational approaches, able to automatically compare numerous biological conditions and systems and to detect their systemic differentiations, using as criterion their semantic description from various ontological scheme.

## Supplementary Material

lqae014_Supplemental_File

## Data Availability

The main part of code written in support of this manuscript, all the results and the data used to produce them, are publicly available in a figshare collection at https://doi.org/10.6084/m9.figshare.c.5427279.v4. Some tasks were performed using the software of e-NIOS Applications PC (BioInfoMiner), so the respective code is not available. However, an open version of BioInfoMiner and that of semantic comparative analysis are available in the Galaxy application http://www.biotranslator.gr:8080 upon registration.
