# Supplementary material for: Protein homeostasis imprinting across evolution
Source: NAR Genom Bioinform. 2024 Feb 15;6(1):lqae014. doi: 10.1093/nargab/lqae014 (PMC10939379; doi:10.1093/nargab/lqae014)
Supplement: lqae014_Supplemental_File [file lqae014_supplemental_file.docx]

**Supplementary Materials & Methods**

**Gain Ratio, Homogeneity and Silhouette Scores**

Gain Ratio (Han et al., 2011) is used as a feature selection measure in data mining. Given a dataset *A*, with samples belonging to a set of classes $C=\left\{ c_{1},c_{2},\ldots,c_{N} \right\}$ and *D* a subset of *A,* the entropy of the classes’ distribution in subset *D* is defined as follows:

$$H\left( D \right)= -\sum_{i=1}^{\left| C_{D} \right|} \frac{\left| C_{i,D} \right|}{\left| D \right|}log\left( \frac{\left| C_{i,D} \right|}{\left| D \right|} \right)$$

where$\left| C_{i,D} \right|$ is the number of samples in partition *D*, which belong to class $C_{i}$. $H\left( D \right)$quantifies the expected information, to classify correctly a sample $a_{i}$ in *D*. This entropy is maximized when each sample belongs to a different class. During the training process of decision trees, discrete or continuous variables (features) are examined to separate samples, with respect to their classes. Assume that a feature *F* is selected to separate further the samples in *D*, producing *M* groups. Then, the entropy of *D* is re-calculated, taking into account that partitioning:

$$H_{F}\left( D \right)= \sum_{j=1}^{\left| M \right|} \frac{\left| M_{j} \right|}{\left| D \right|}\times H(M_{j})$$

which is the weighted mean entropy of the derived *M* subsets. Information Gain of feature *F* is equal to the reduction of entropy after the split:

$$InfoGain\left( F \right)= H\left( D \right)- H_{F}\left( D \right)$$

Another useful measure is the Split Entropy, which is defined as the derived uncertainty due to the partitioning of samples:

$${Hsplit}_{F}\left( D \right)=- \sum_{j=1}^{\left| M \right|} \frac{\left| M_{j} \right|}{\left| D \right|}\times log\left( \frac{\left| M_{j} \right|}{\left| D \right|} \right)$$

Split Entropy value increases in function with the amount of the produced subsets and it is maximized when feature *F* creates a novel branch for each sample. It could function as a penalty factor, in order to avoid the selection of features, which tend to segregate the dataset into numerous clusters. Gain Ratio uses that factor to normalize the Information Gain, providing an unbiased measure of splitting information:

$$GainRatio\left( F \right)= \frac{InfoGain(F)}{{Hsplit}_{F}\left( D \right)}$$

Homogeneity (HS) (Rosenberg & Hirschberg, 2007) and silhouette (SS) (Rousseeuw, 1987) scores evaluate specific properties of an unsupervised clustering outcome, given the true classes of the data samples and their pairwise distances. Assuming that the samples of dataset *A* are being classified into a set of clusters $K=\left\{ k_{1},k_{2},\ldots,k_{m} \right\}$ by an unsupervised clustering algorithm. The number of samples of class $i$ which are assigned to cluster $j$ is denoted as$\left| A_{ij} \right|$. HS evaluates the quality of the derived *K* clusters to contain objects belonging to a unique class, by measuring the conditional entropy of the classes’ distribution given the proposed clustering:

$$H\left( K \right)=- \sum_{k=1}^{\left| K \right|} \sum_{c=1}^{\left| C \right|} \frac{\left| A_{ck} \right|}{\left| A \right|}log\left( \frac{\left| A_{ck} \right|}{\left| A_{c} \right|} \right)$$

Formally, $H\left( K \right)$ quantifies the uncertainty about the distribution of samples in the set of $C$ classes, given the *K* clusters. If each cluster$k$ is homogenous (i.e. it contains samples from only one class), then the conditional entropy $H\left( K \right)$ is equal to zero. The conditional entropy is maximum (equal to $H\left( A \right)$ - the entropy of the classes’ distribution in *A*) when the proposed *K*-clustering does not provide any information about the real classification. Homogeneity score is defined as:

$$HS= 1- \frac{H\left( K \right)}{H\left( A \right)}$$

Using both the normalized entropy and the subtraction, HS is bounded in the range [0, 1] and its desirable value is equal to 1. Silhouette score is a measure of cluster cohesion membership, as it quantifies the trade-off between intra- and inter-distances of the derived clusters. Given the aforementioned *K*-clustering, for each sample *i* assigned to cluster $k_{n}$, the factors $f_{1}\left( i \right)$ and$f_{2}\left( i \right)$ are defined as follow:

$$f_{1}\left( i \right)= \frac{1}{\left| k_{n} \right|-1}\sum_{j\in k_{n},j\neq i}^{\left| k_{n}-1 \right|} d(i,j)$$

$$f_{2}\left( i \right)= \left[ \frac{1}{\left| k_{m} \right|}\sum_{j\in k_{m}}^{\left| k_{m} \right|} d(i,j) \right]$$

where$d(i,j)$ is the distance between samples *i* and *j*. The factor $f_{1}\left( i \right)$ is the average distance of sample *i* to the other samples in cluster$k_{n}$ and indicates the merit of the assignment to that cluster. The factor $f_{2}\left( i \right)$ is the minimum average distance of sample *i* to all samples in any other cluster, apart from cluster$k_{n}$. Namely $f_{2}\left( i \right)$ measures the inter-distance of sample *i* to its neighboring cluster. The silhouette coefficient of sample *i* is defined as:

$${SS}_{i}=\{\frac{f_{2}\left( i \right)-f_{1}\left( i \right)}{\left\{ {f_{1}\left( i \right), f}_{2}\left( i \right) \right\}} if \left| k_{n} \right|>1 0 if \left| k_{n} \right|=1$$

${SS}_{i}$ranges from -1 to +1, where a high value implies that *i* is well-located in its group and far from the other clusters (without considering if it is correctly classified). The mean silhouette over all data samples measures the average cohesion of the derived clusters:

$$SS= \frac{1}{\left| A \right|}\sum_{i\in A}^{\left| A \right|} ss(i)$$

**Supplementary Figures**


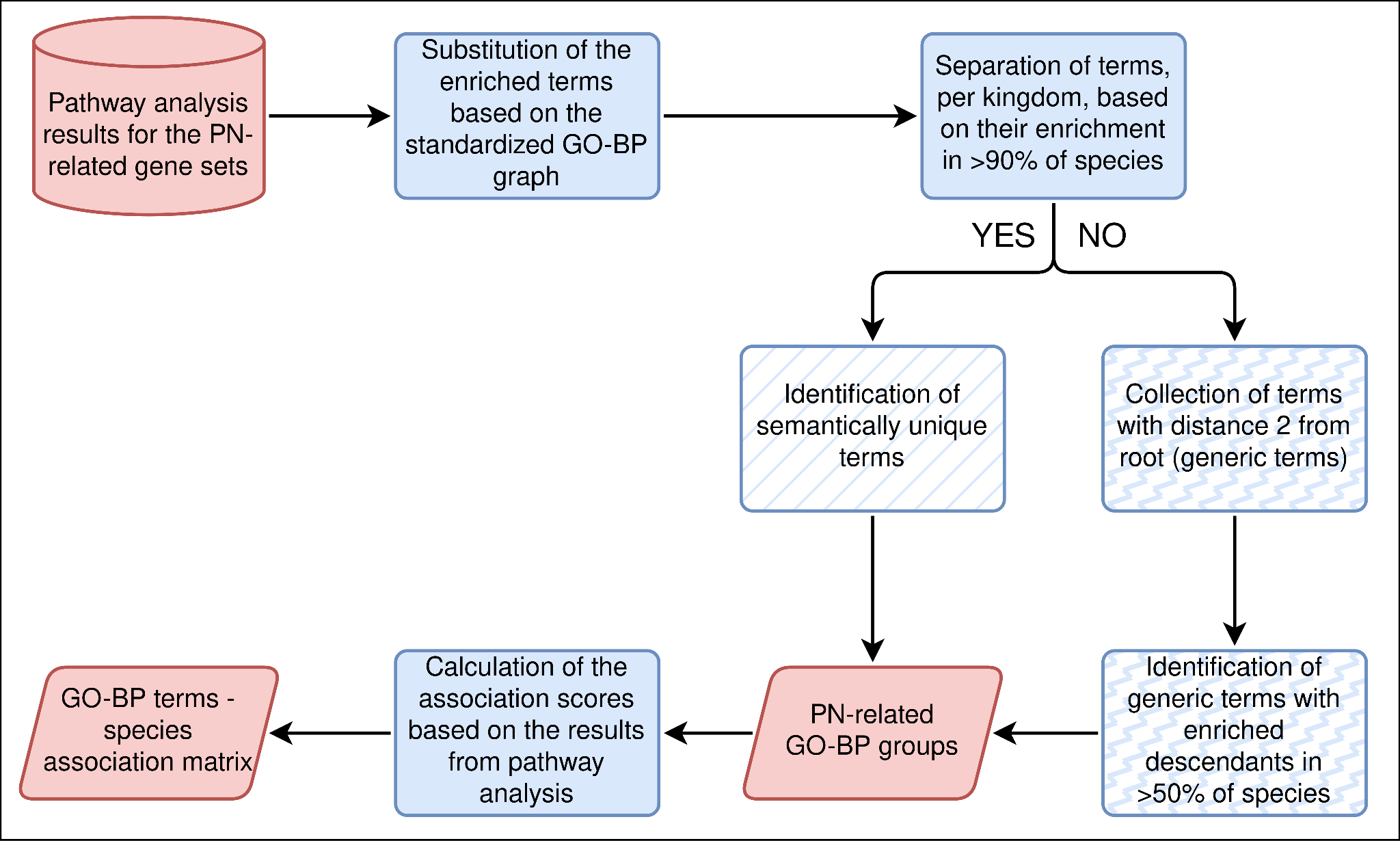


Figure S1.1: Schematic representation of the developed workflow for the identification of PN-related semantic groups, based on the results of pathway analysis.


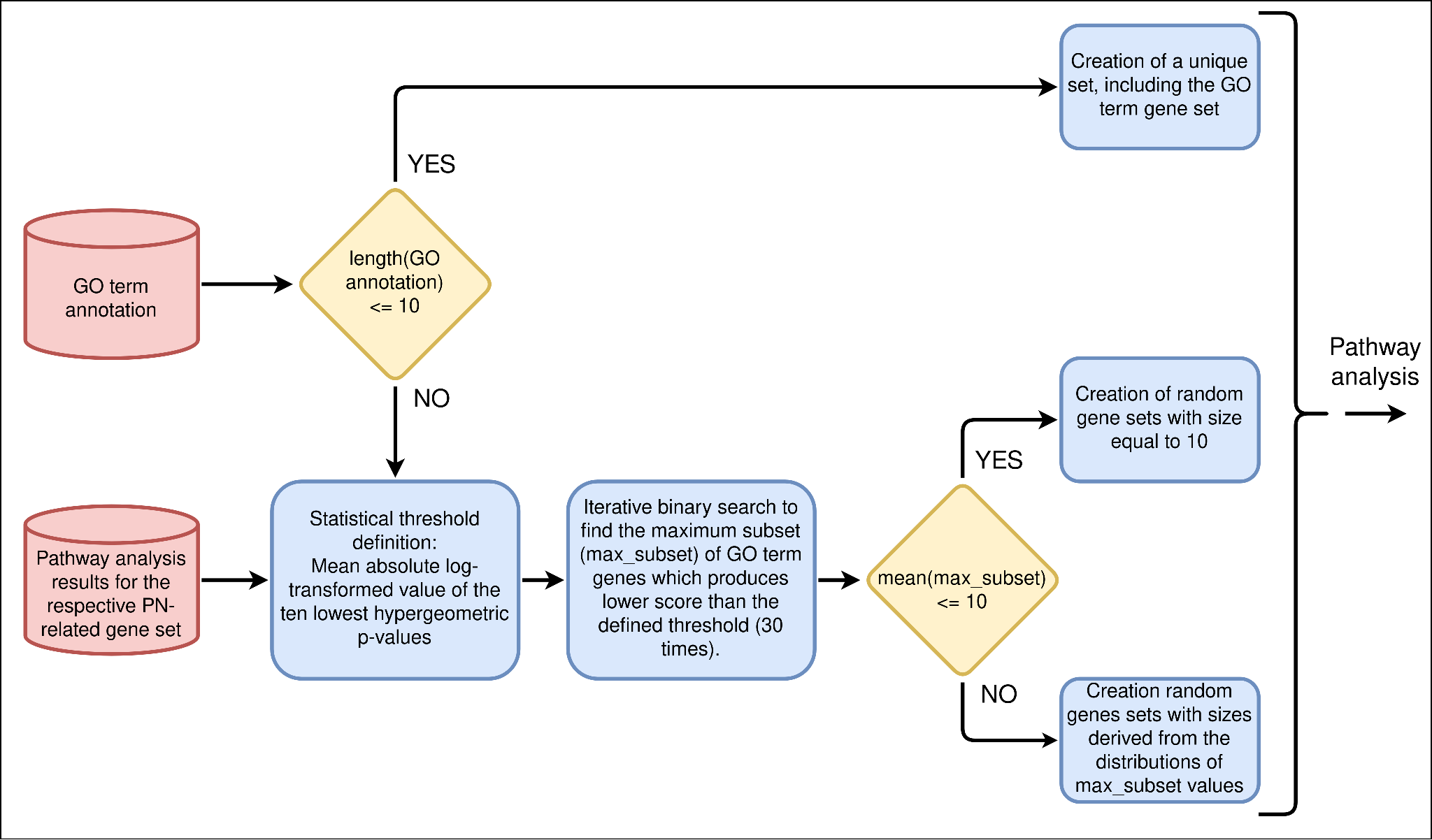


Figure S1.2: Schematic representation of the developed workflow for the determination of gene sets, used to derive the semantic profiles of the selected conserved mechanisms, through pathway analysis.


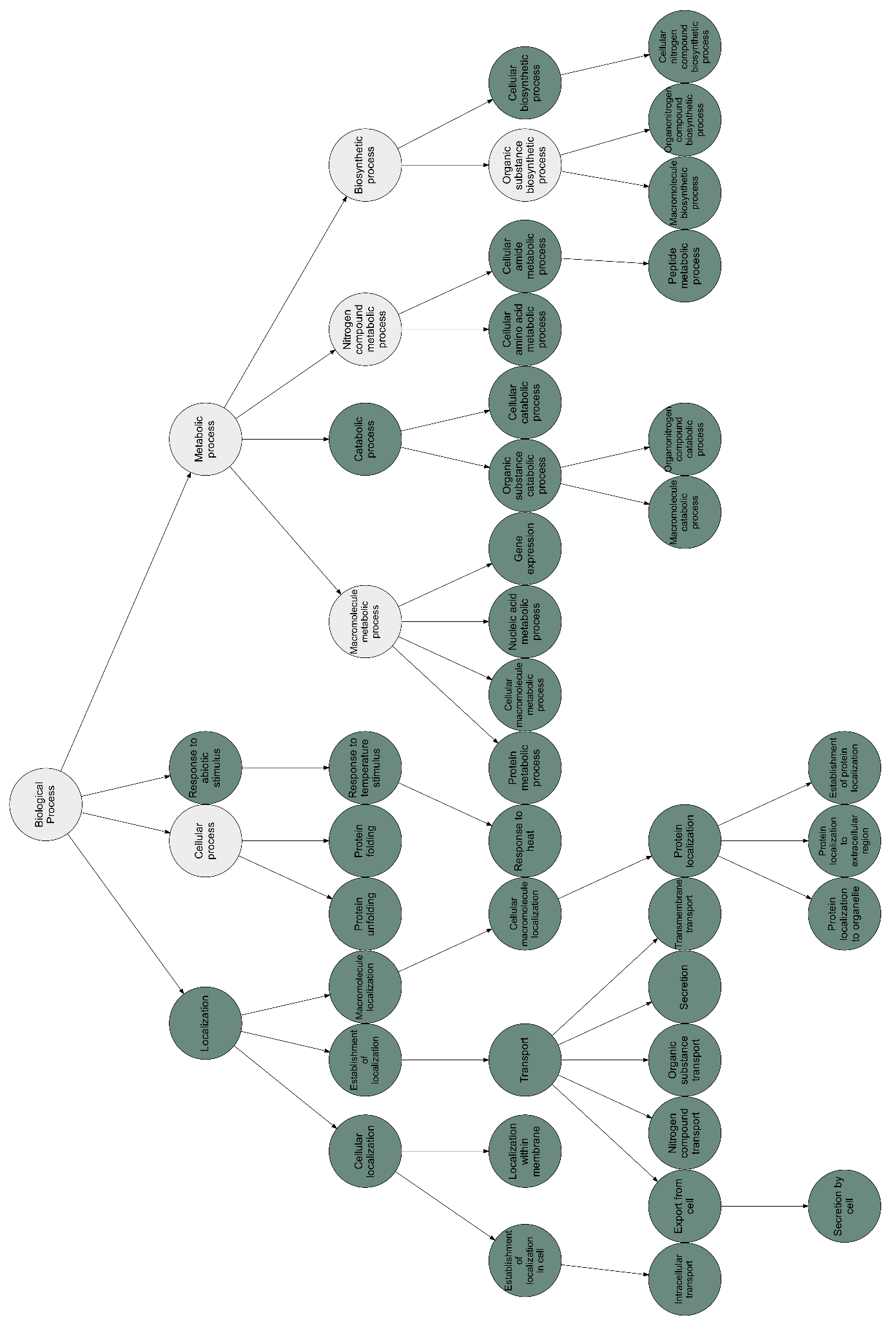


Figure S2.1: The PN semantic network for *Halomarina oriensis*, after the projection of the enriched terms on the standardized GO-BP graph.


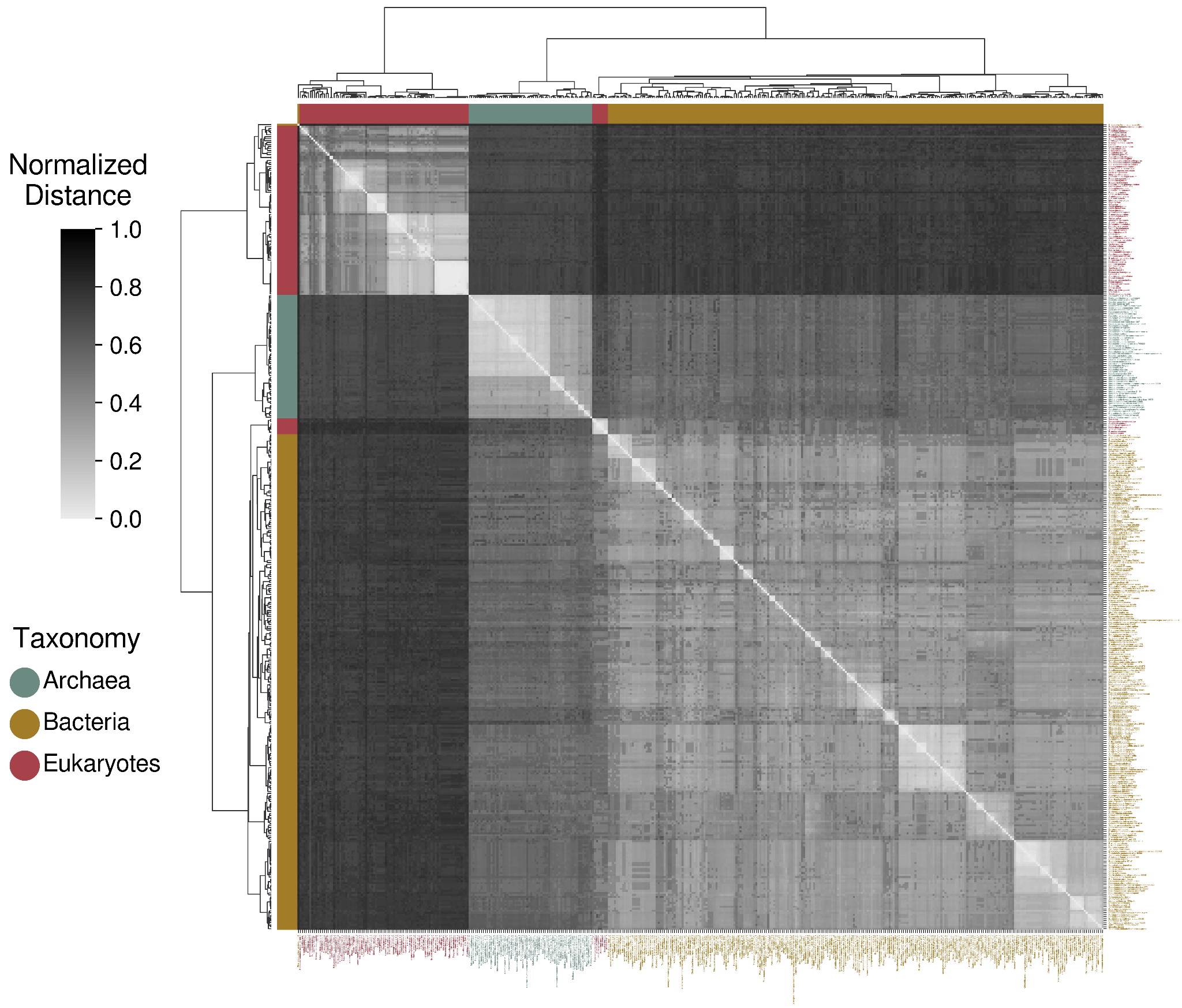


Figure S2.2: Phylogenetic clustergram derived from the comparison of the genomic ribosomal sequences (18S for eukaryotes and 16S for prokaryotes), using the ClustalW algorithm.


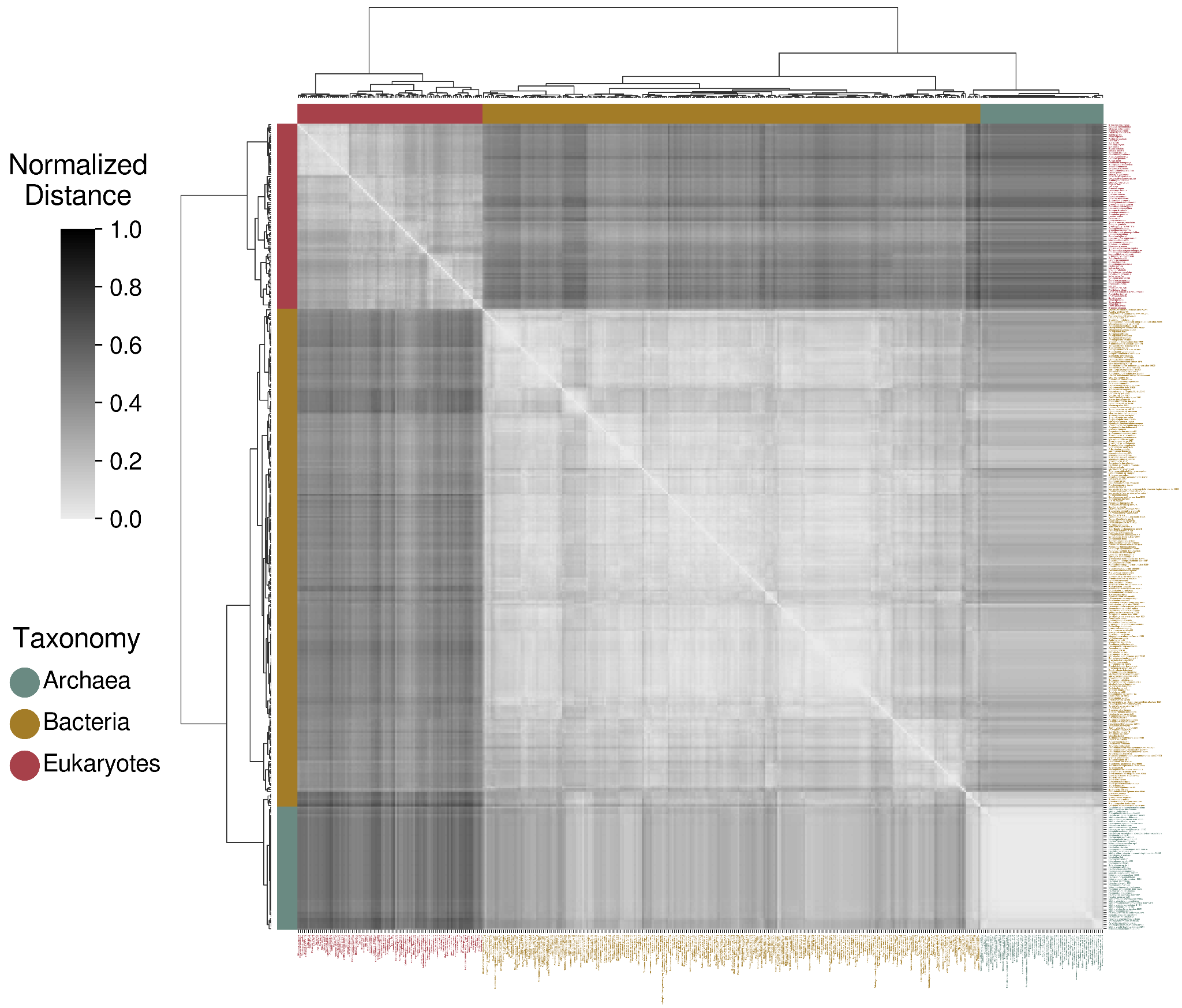


Figure S2.3: Phylogenetic clustergram derived from the comparison of proteostasis semantic networks. The networks were constructed performing pathway analysis on proteostasis related gene sets, using the GO-BP domain. Semantic analysis operators were applied to compare the networks based on the GO-BP graph topology.


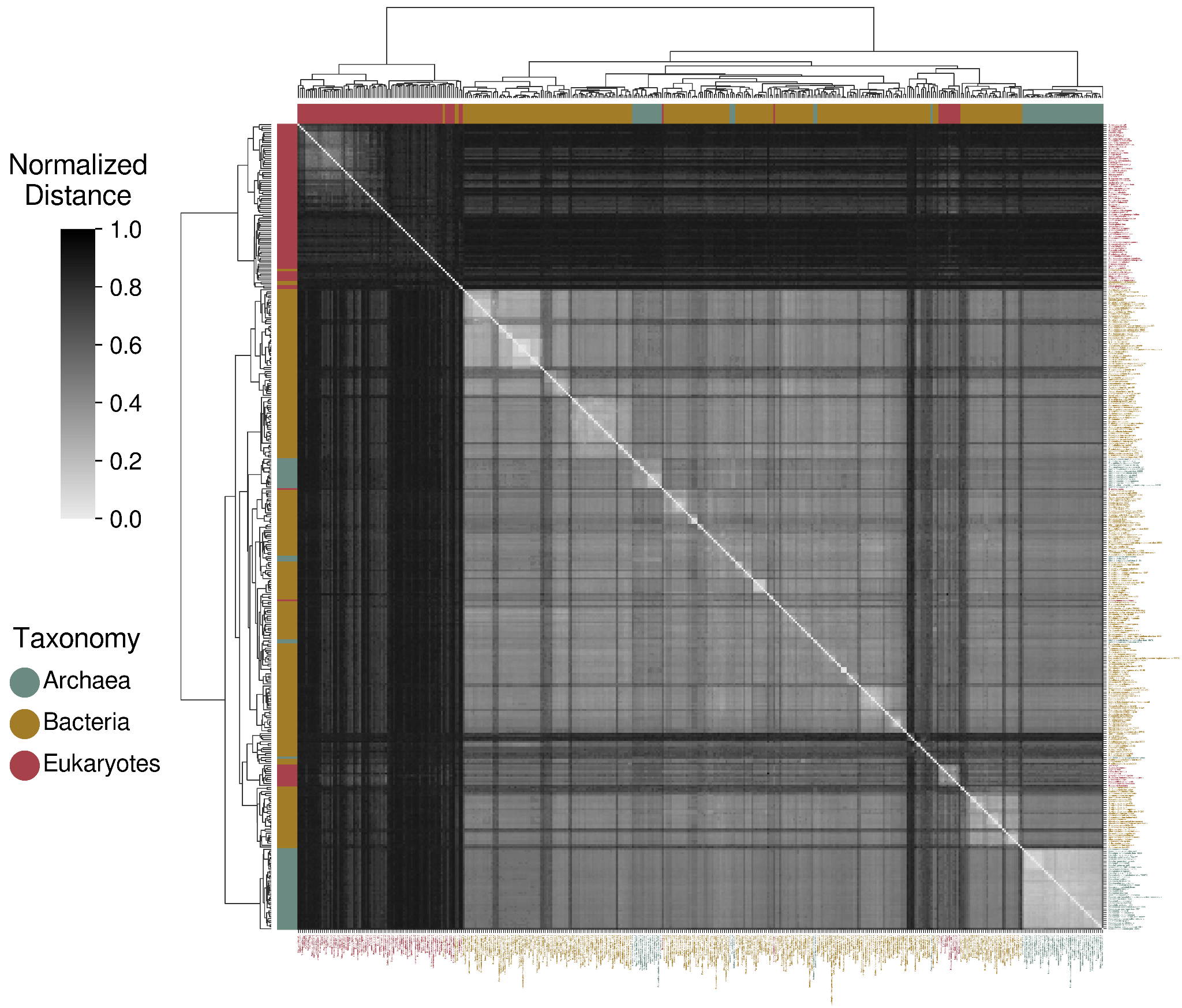
Figure S2.4: Phylogenetic clustergram derived from the comparison of heat shock protein 40kDa (HSP40) sequences. The CD-HIT and HMMER3 tools were used to construct a consensus amino acid sequence for each species. The phylogenetic comparison was performed using the ClustalW tool.


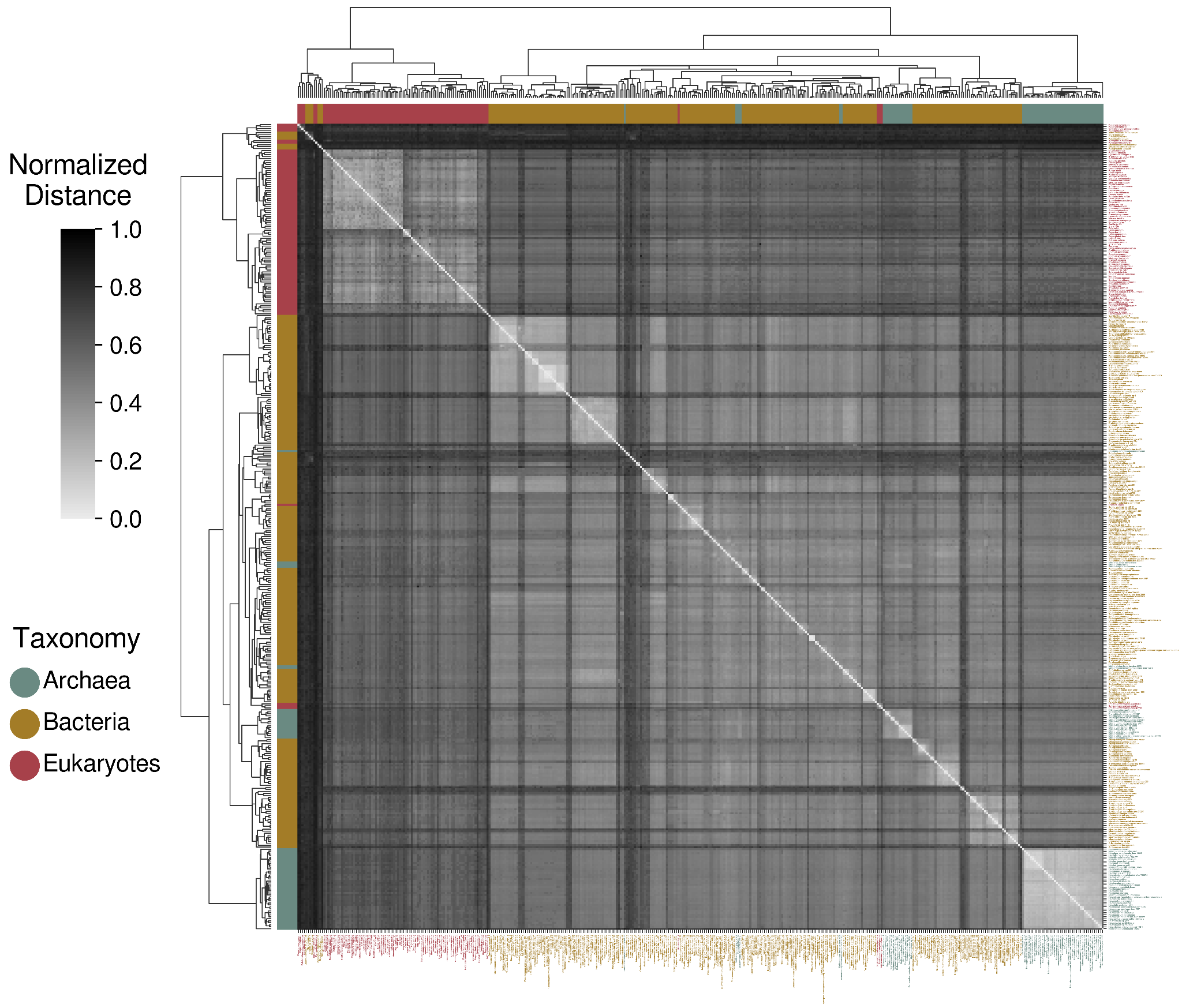


Figure S2.5: Phylogenetic clustergram derived from the comparison of heat shock protein 70kDa (HSP70) sequences. The CD-HIT and HMMER3 tools were used to construct a consensus amino acid sequence for each species. The phylogenetic comparison was performed using the ClustalW tool.


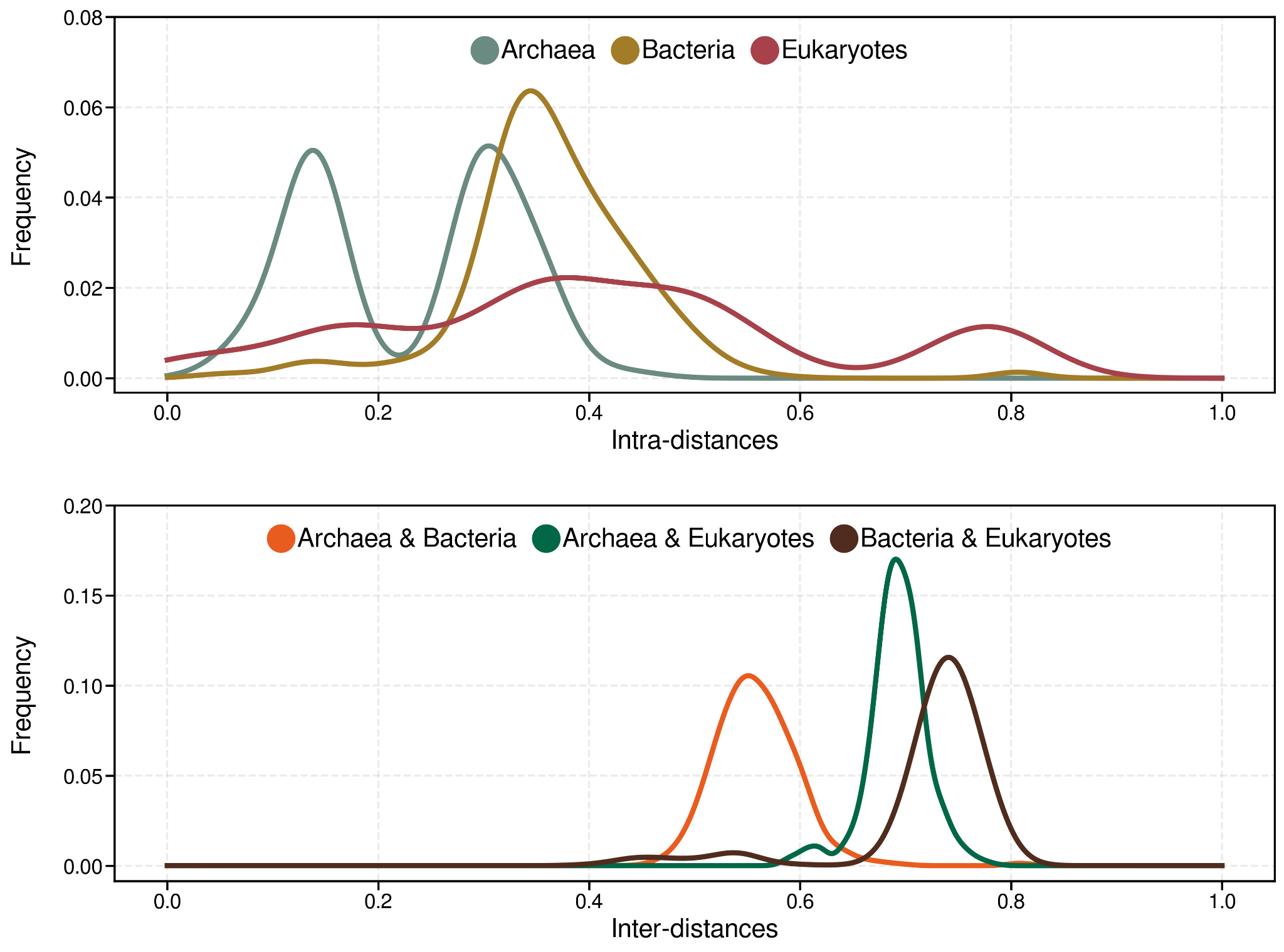
Figure S2.6: Distributions of pairwise distances of rRNA sequences in each taxonomic domain (intra-distances) and between the different domains (inter-distances).


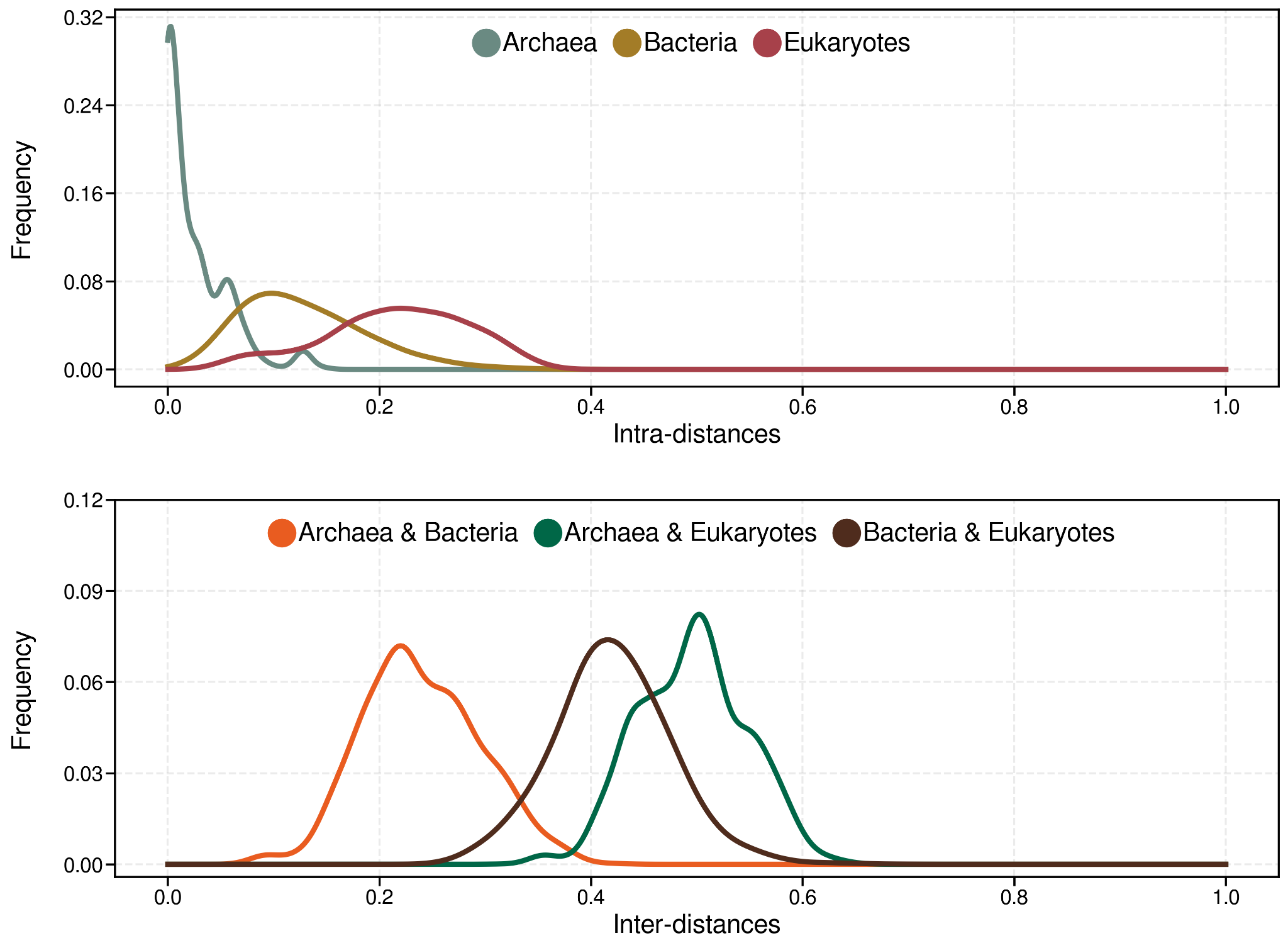
Figure S2.7: Distributions of pairwise PN-semantic distances in each taxonomic domain (intra-distances) and between the different domains (inter-distances).


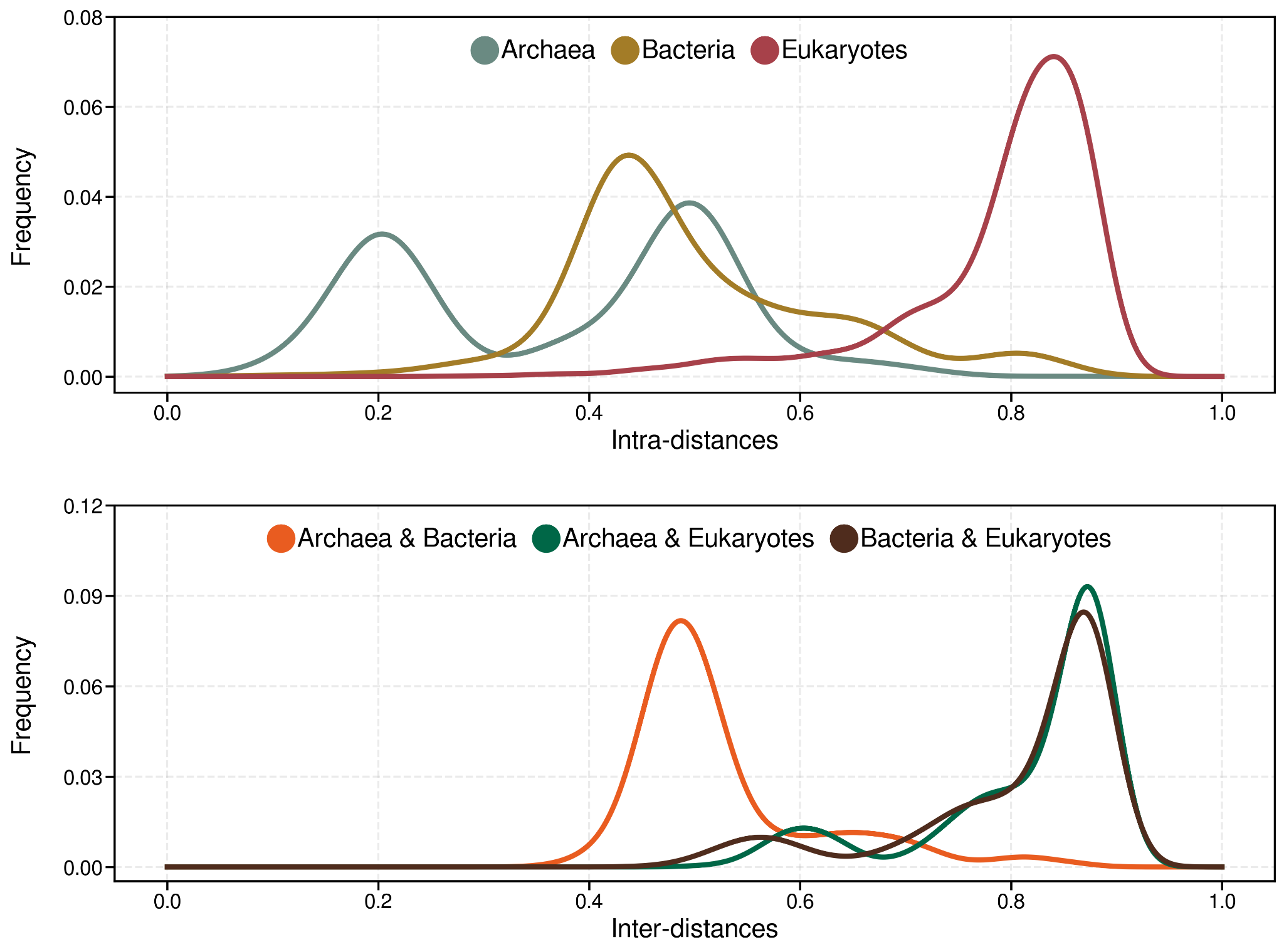
Figure S2.8: Distributions of pairwise distances of HSP40 sequences in each taxonomic domain (intra-distances) and between the different domains (inter-distances).


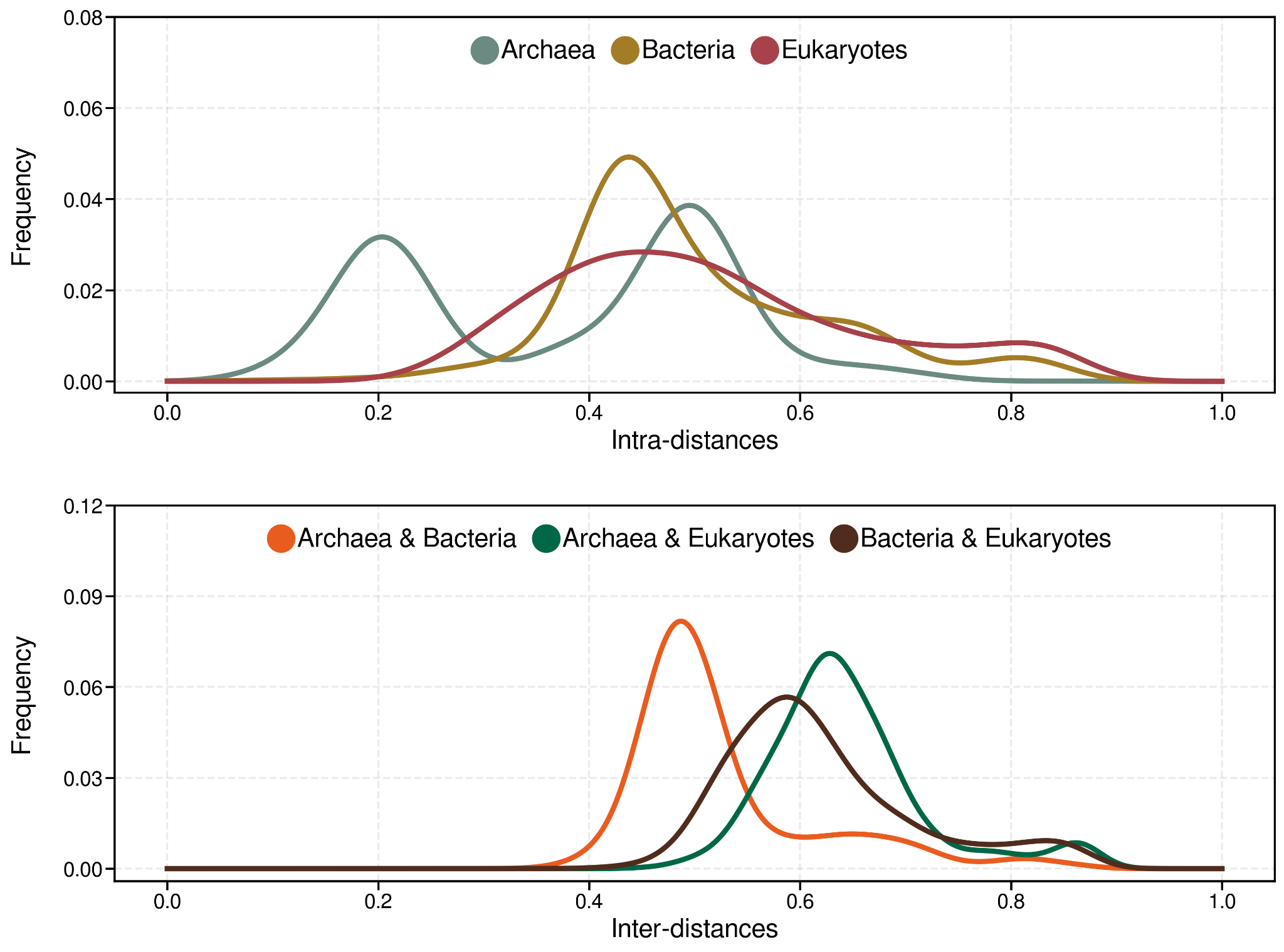
Figure S2.9: Distributions of pairwise distances of HSP70 sequences in each taxonomic domain (intra-distances) and between the different domains (inter-distances).


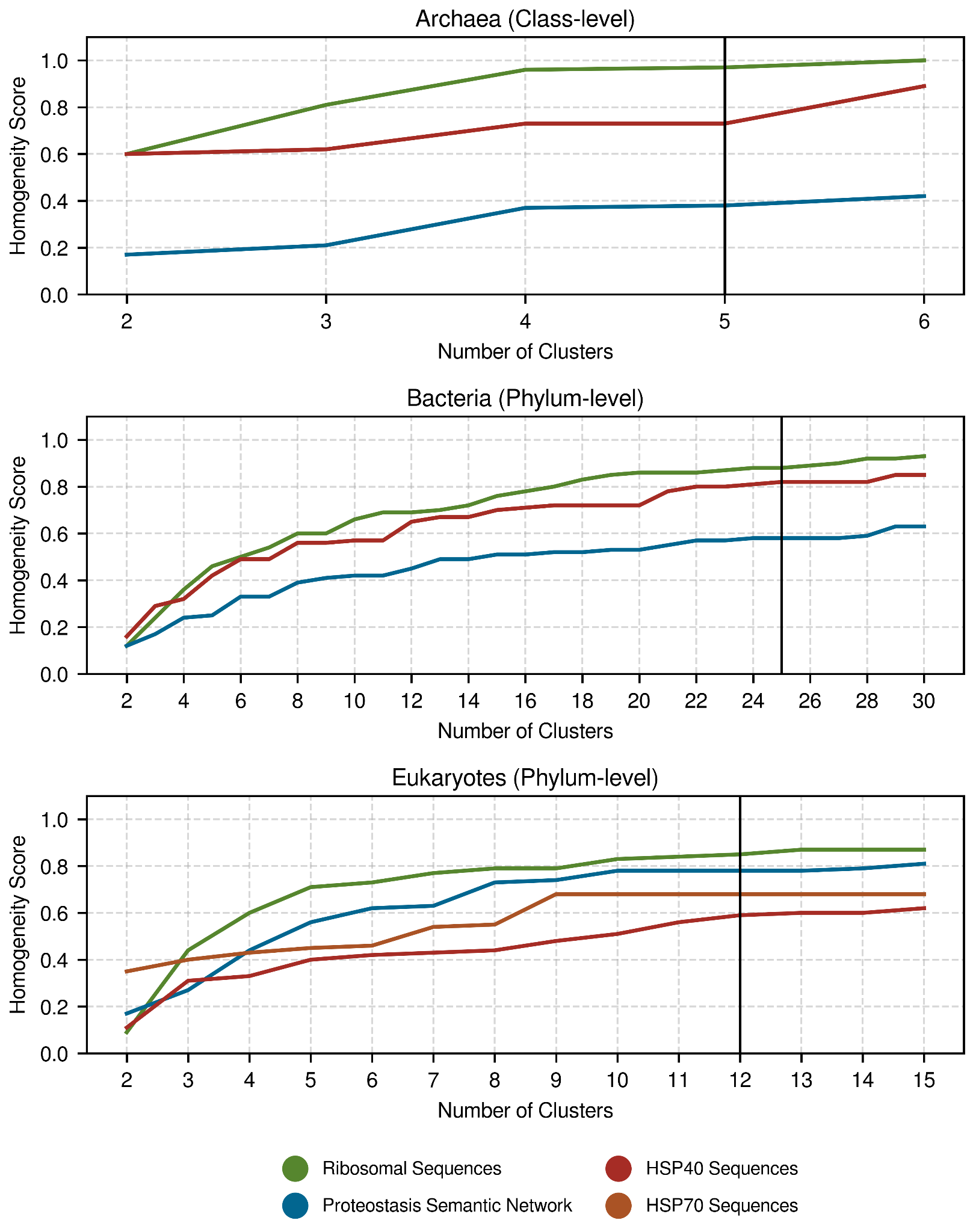


Figure S2.10: Evaluation of rRNA, HSP40, HSP70 and PN to separate effectively the species of the same domain into reference taxonomic sub-groups (Class-level for Archaea and Phylum-level for Bacteria and Eukaryotes). Different amounts of clusters were generated for each taxonomic category and the homogeneity score was calculated for each clustering outcome, based on the reference taxonomic classification of species. Vertical lines indicate the amount of reference taxonomic sub-groups.


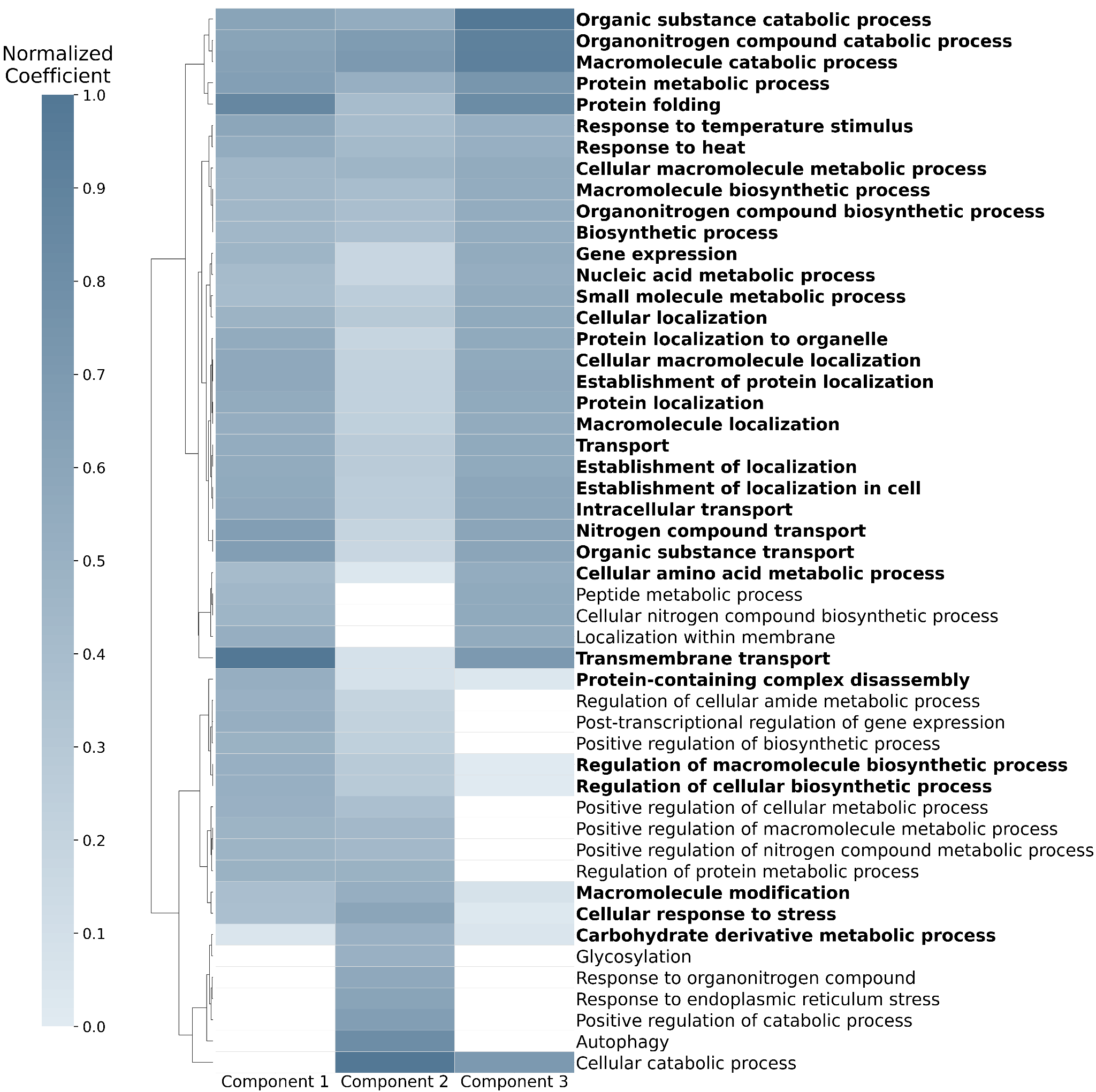
Figure S2.11: The features (components) matrix (n=3) obtained by applying non-negative matrix factorization to the association matrix between species and the PN-related GO-BP groups. The normalized coefficients indicate the contribution of each semantic group to the three NMF-based components. Bold semantic terms indicate the “conserved core” of PN profile across species.


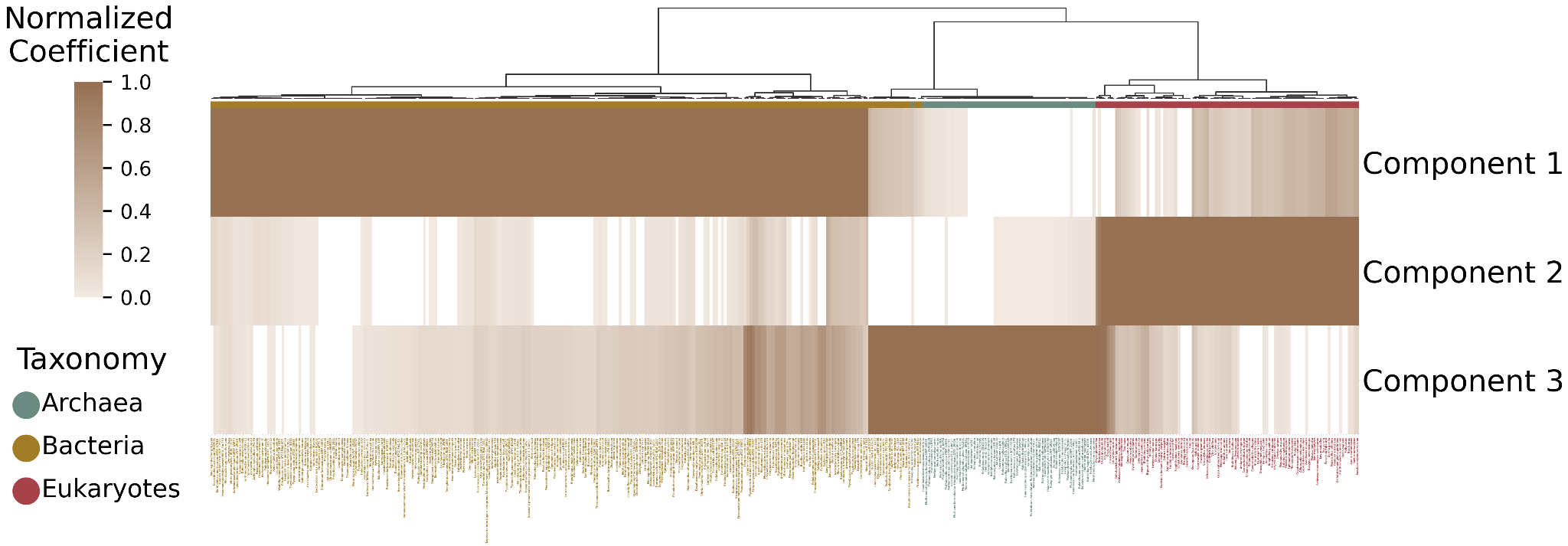
Figure S2.12: The coefficients matrix obtained by applying non-negative matrix factorization to the association matrix between species and the PN-related GO-BP groups. The normalized coefficients indicate the association between the species and NMF-based components.


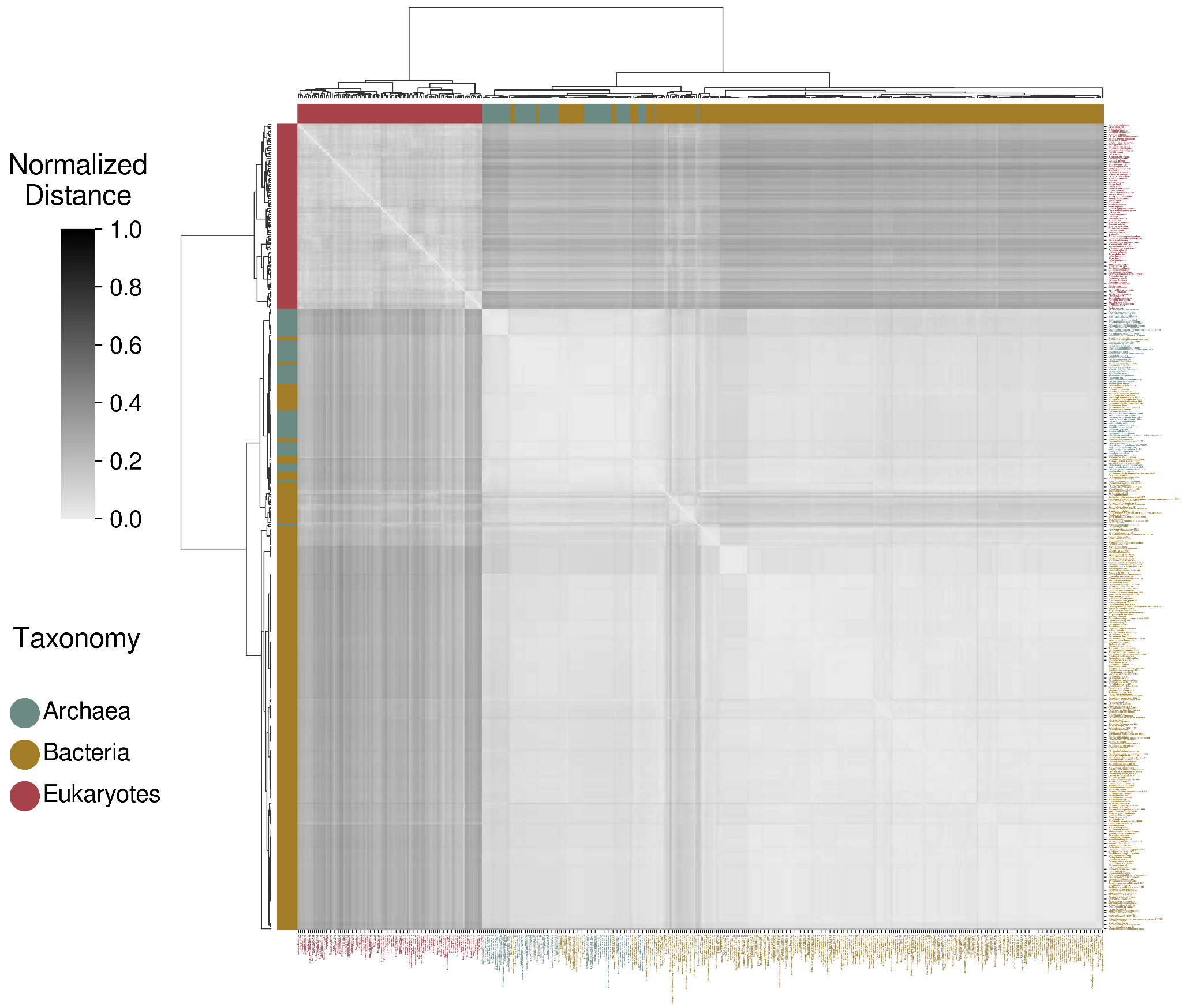


Figure S2.13A: Phylogenetic clustergram derived from the comparison of “ATP metabolic process” semantic networks.


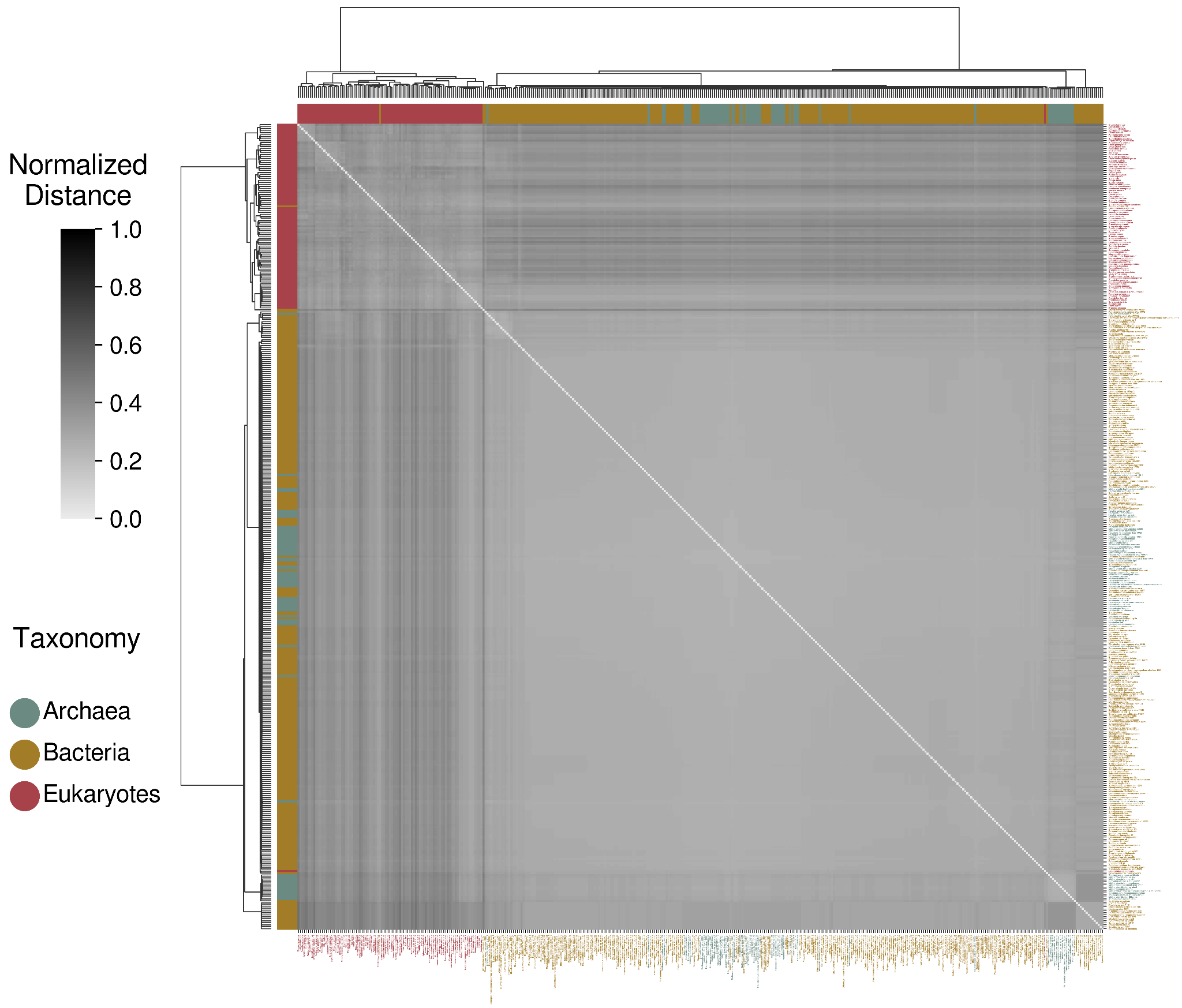


Figure S2.13B: Phylogenetic clustergram derived from the comparison of “ATP metabolic process” semantic networks, excluding terms associated with the obtained PN-related semantic groups.


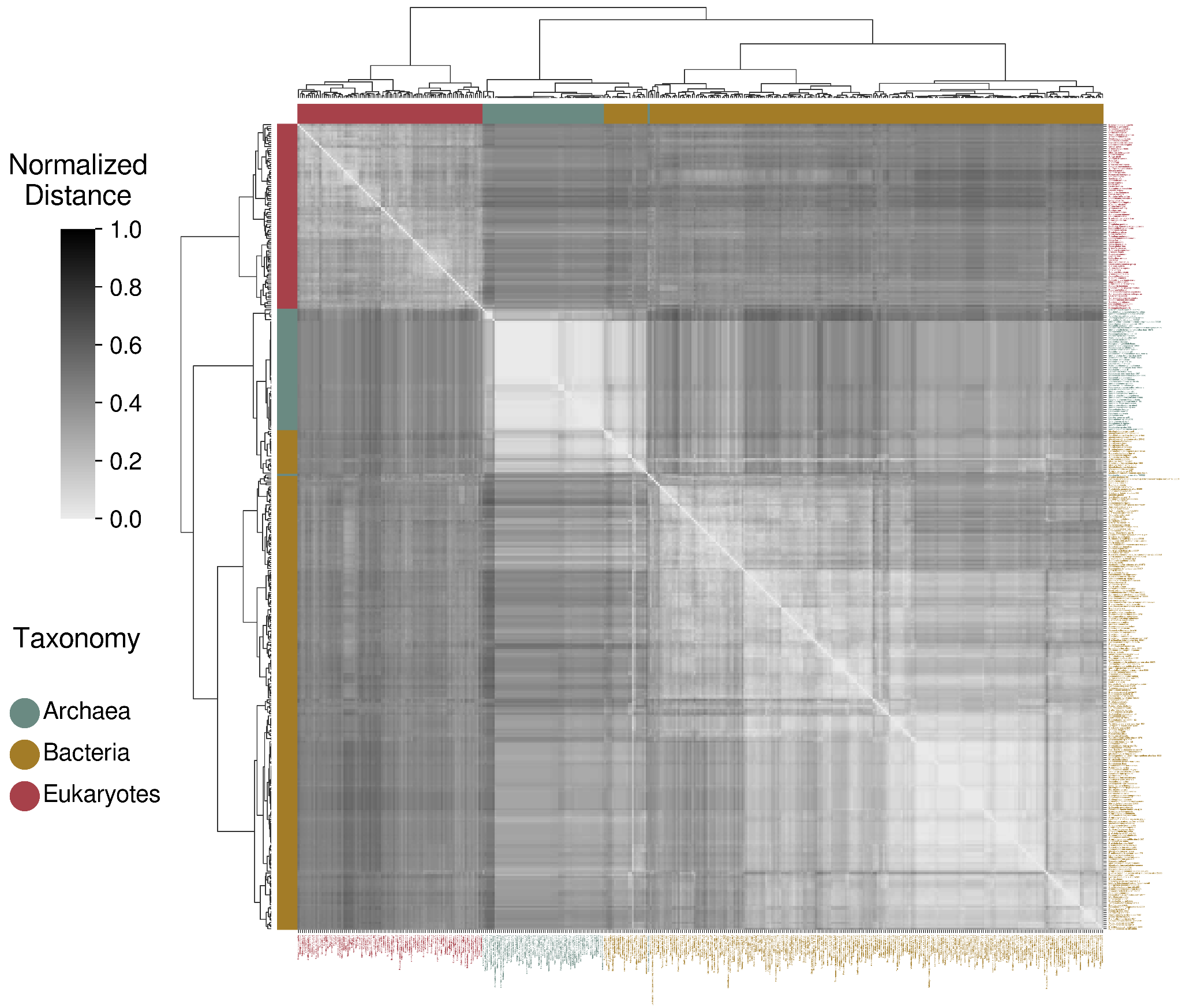


Figure S2.14A: Phylogenetic clustergram derived from the comparison of “cellular component assembly” semantic networks.


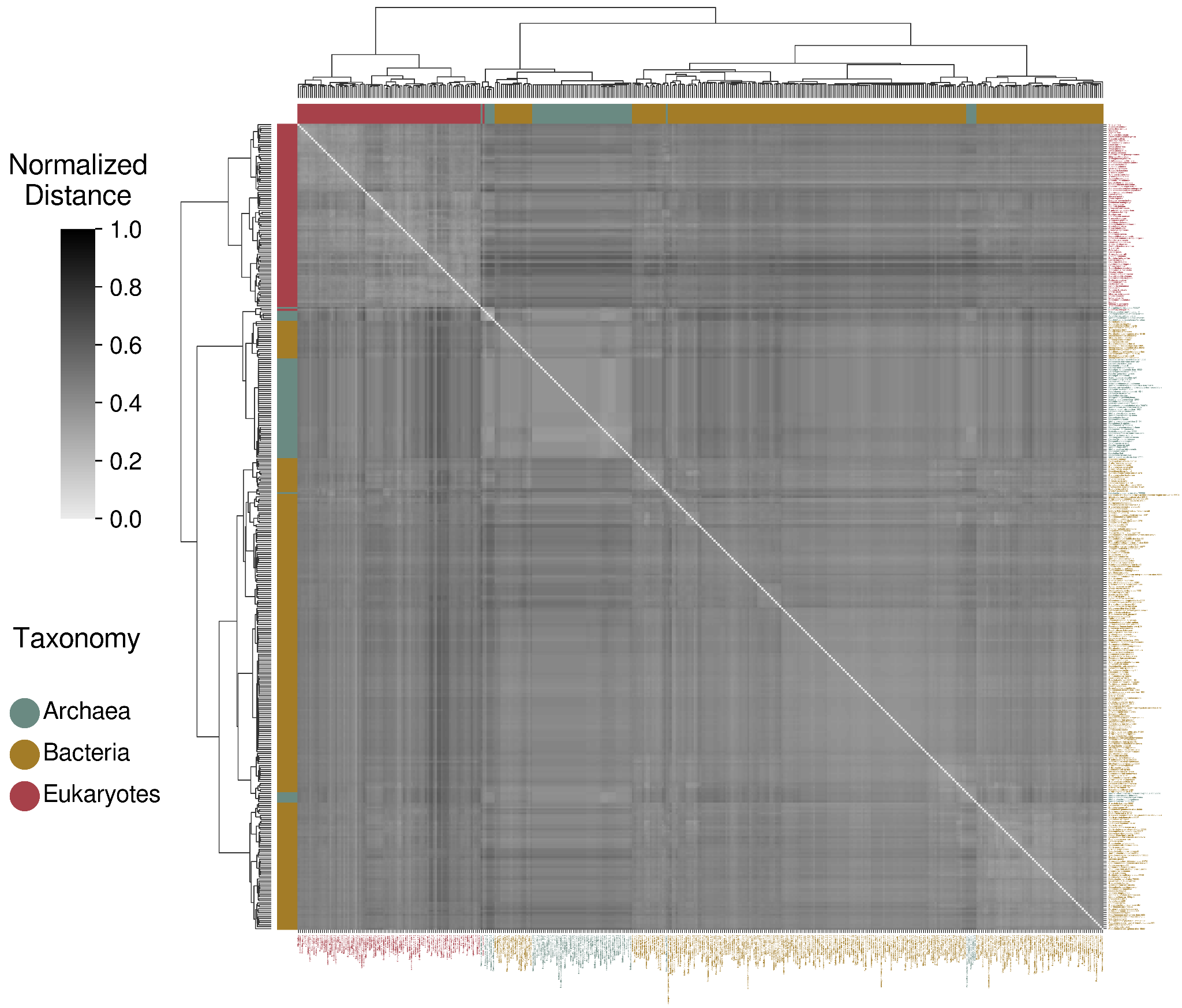


Figure S2.14B: Phylogenetic clustergram derived from the comparison of “cellular component assembly” semantic networks, excluding terms associated with the obtained PN-related semantic groups.


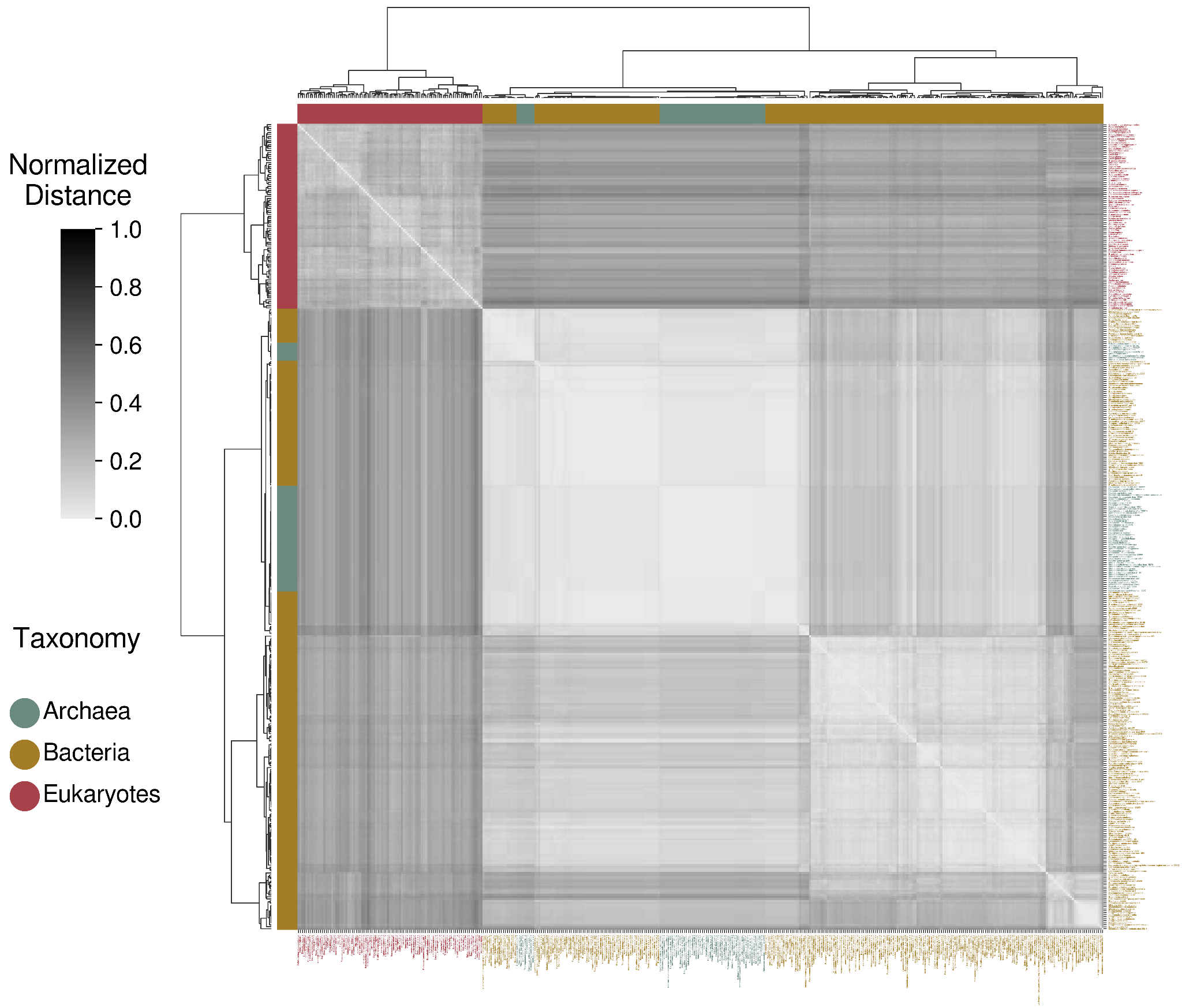


Figure S2.15A: Phylogenetic clustergram derived from the comparison of “cellular localization” semantic networks.


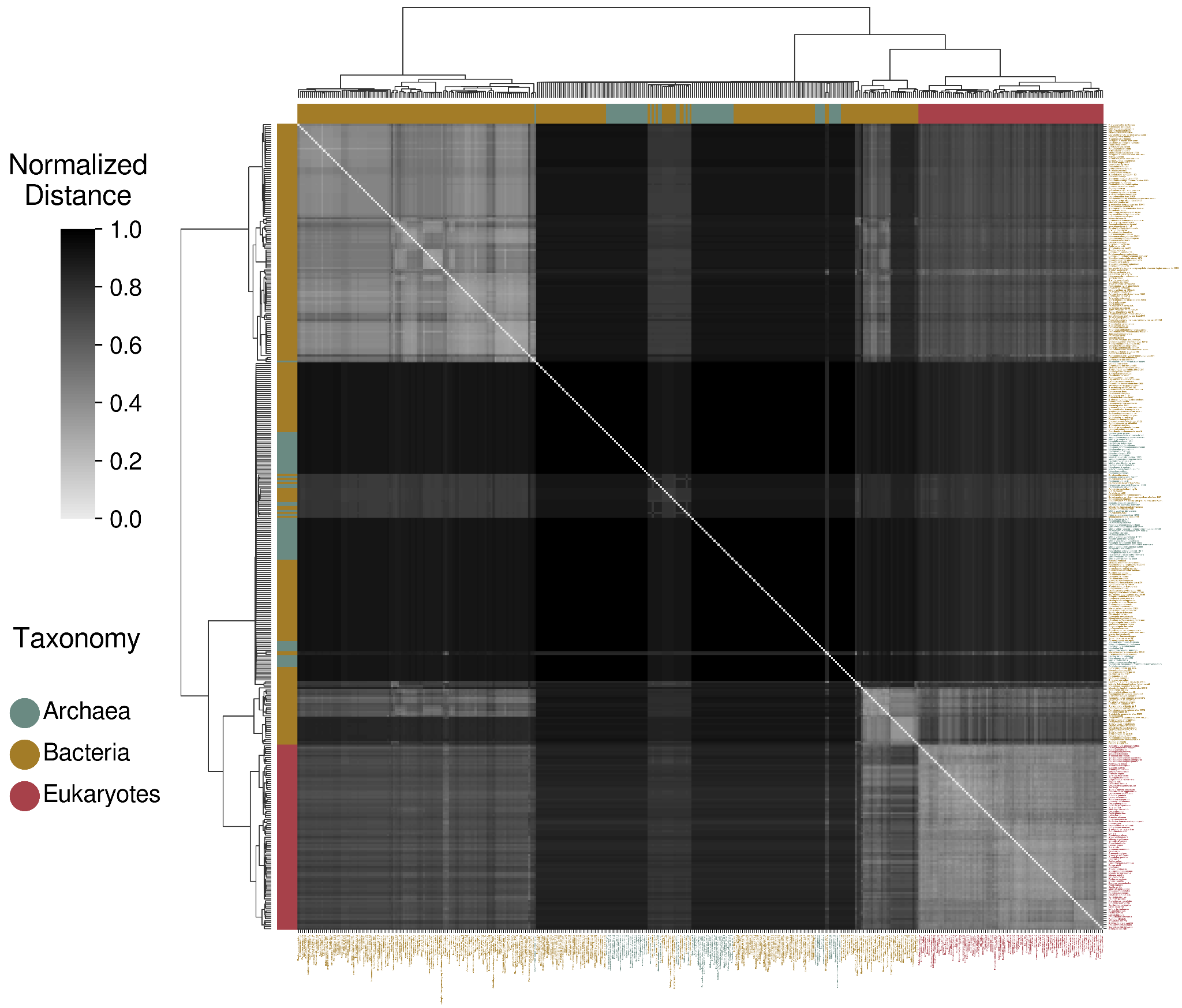


Figure S2.15B: Phylogenetic clustergram derived from the comparison of “cellular localization” semantic networks, excluding terms associated with the obtained PN-related semantic groups.


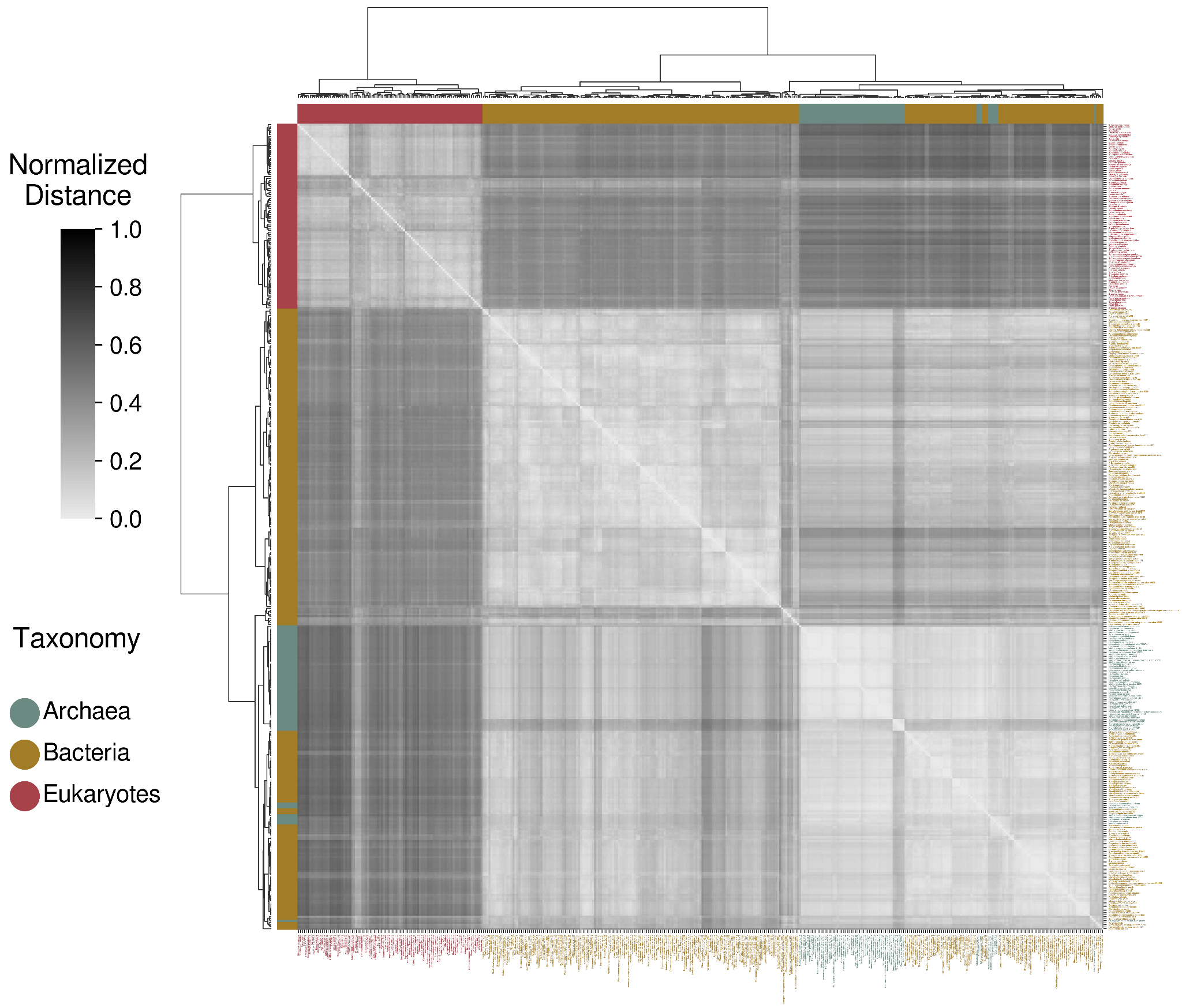


Figure S2.16A: Phylogenetic clustergram derived from the comparison of “cellular response to DNA damage stimulus” semantic networks.


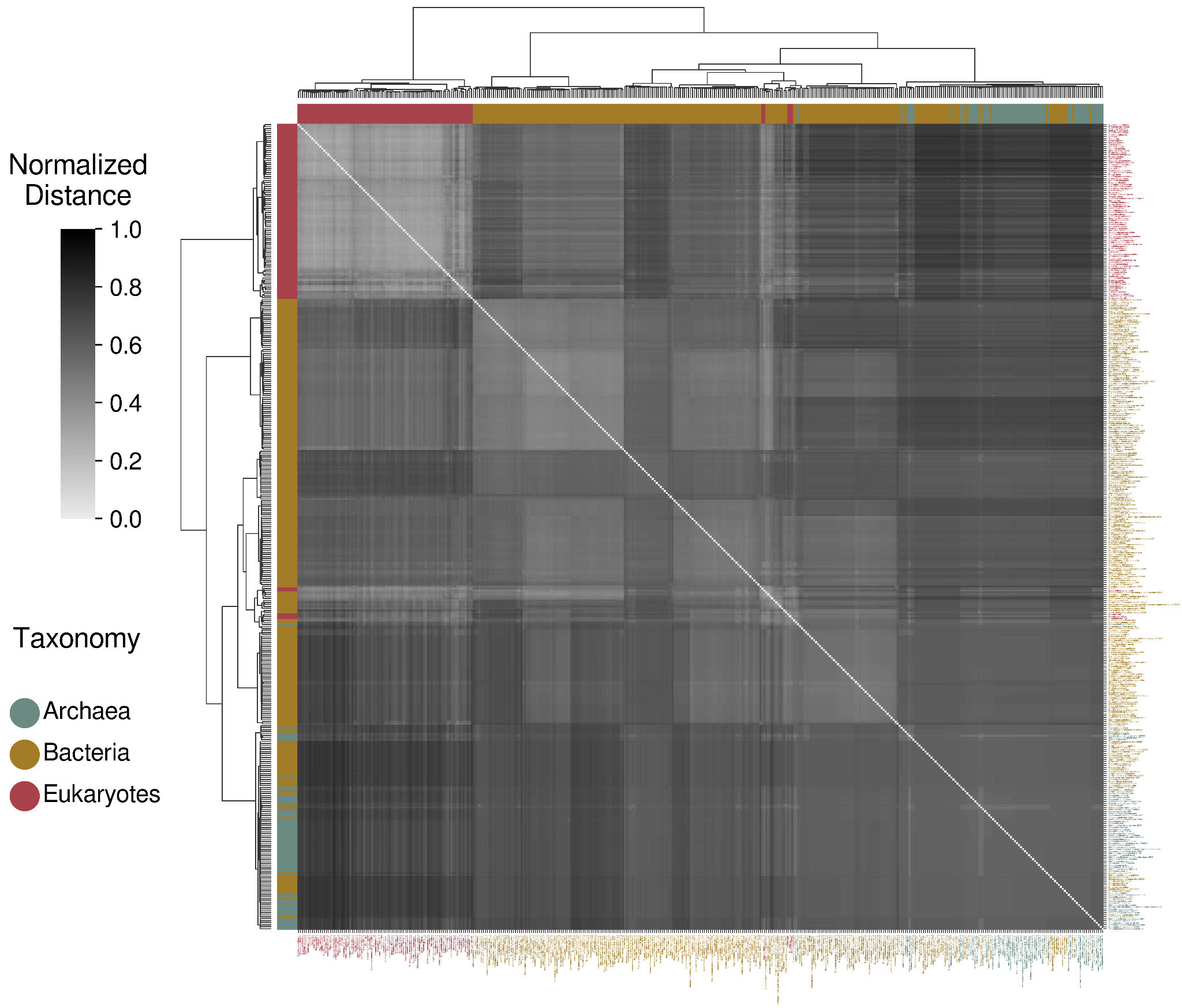


Figure S2.16B: Phylogenetic clustergram derived from the comparison of “cellular response to DNA damage stimulus” semantic networks, excluding terms associated with the obtained PN-related semantic groups.


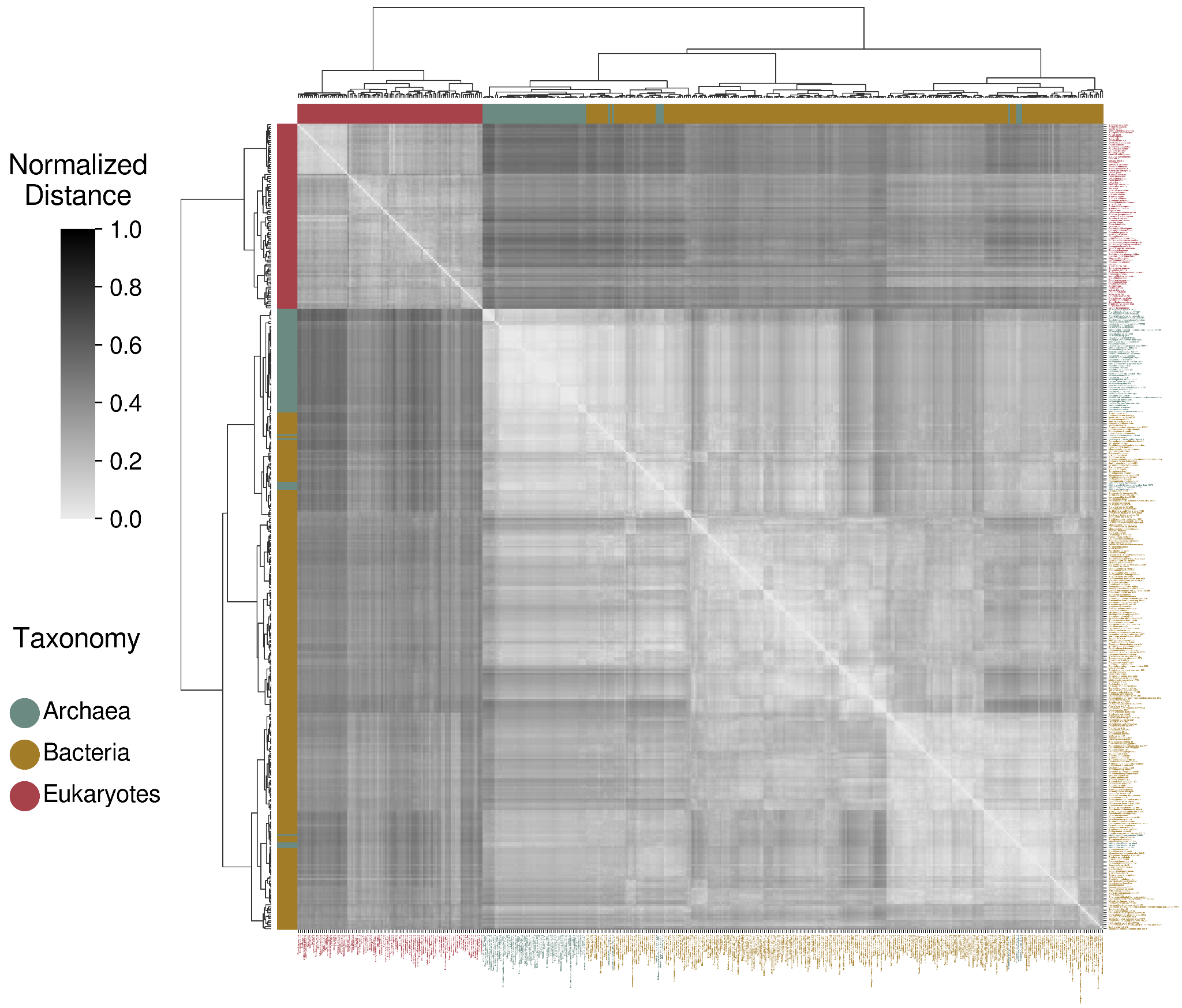


Figure S2.17A: Phylogenetic clustergram derived from the comparison of “cellular response to stress” semantic networks.


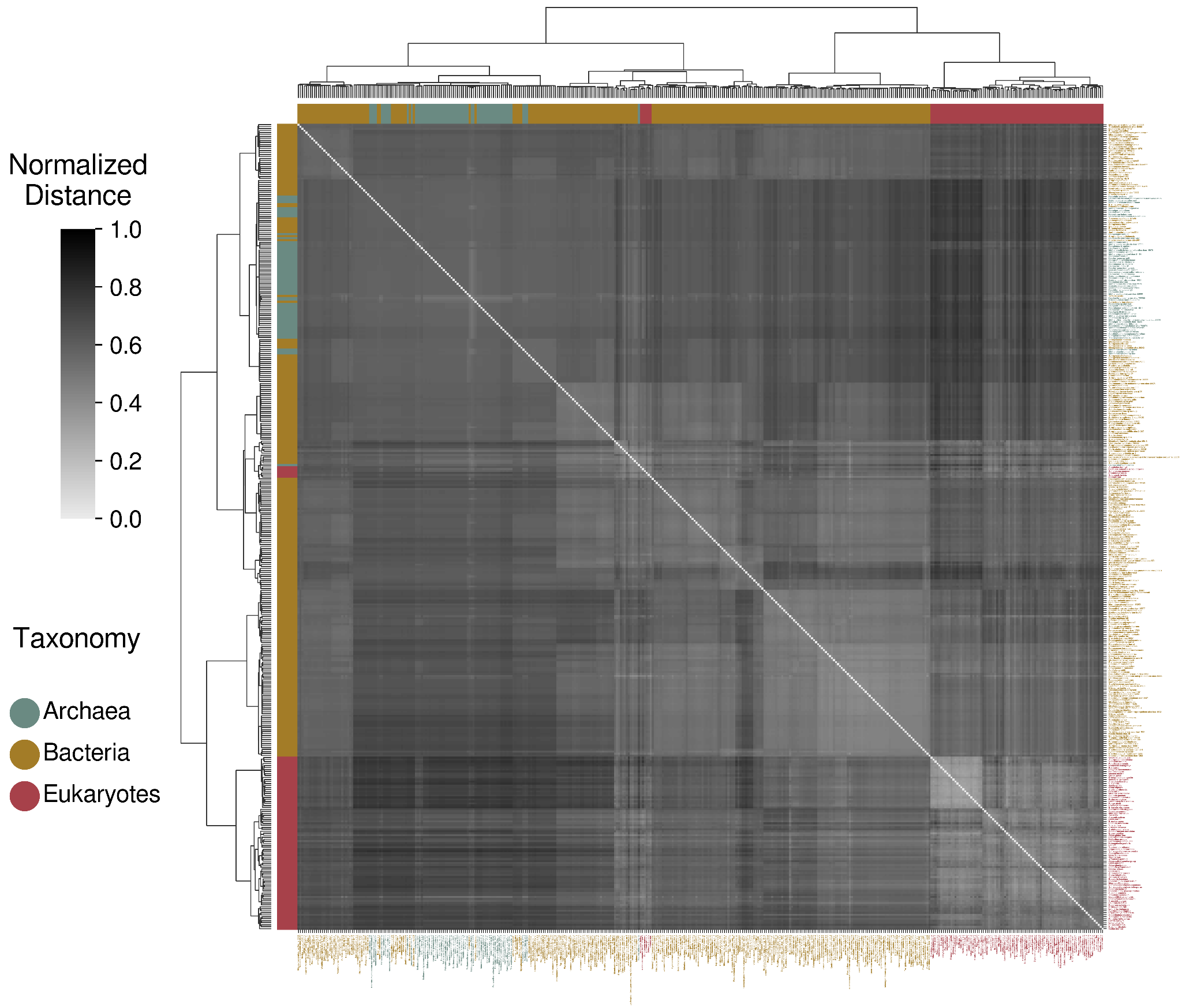


Figure S2.17B: Phylogenetic clustergram derived from the comparison of “cellular response to stress” semantic networks, excluding terms associated with the obtained PN-related semantic groups.


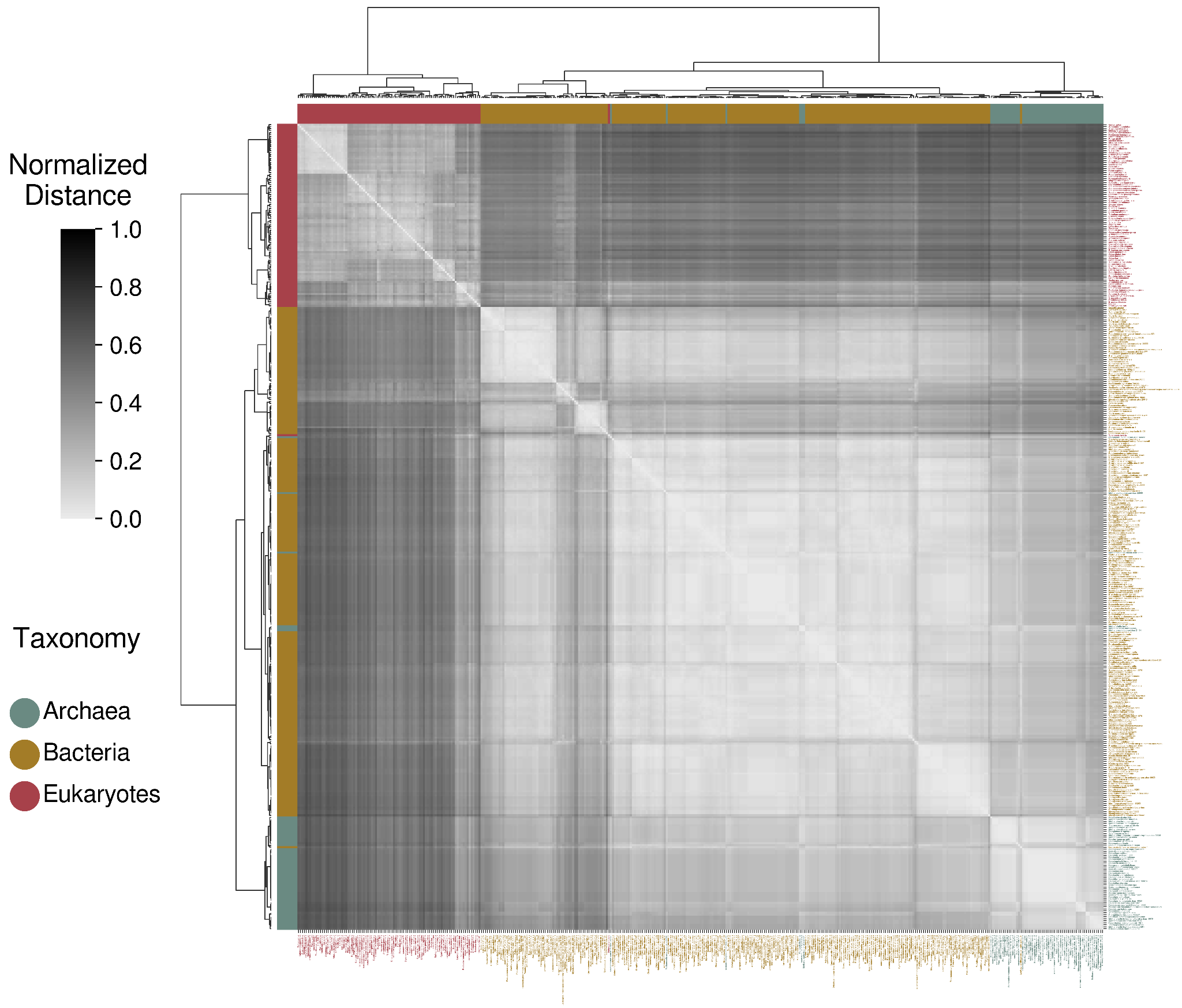


Figure S2.18A: Phylogenetic clustergram derived from the comparison of “DNA recombination” semantic networks.


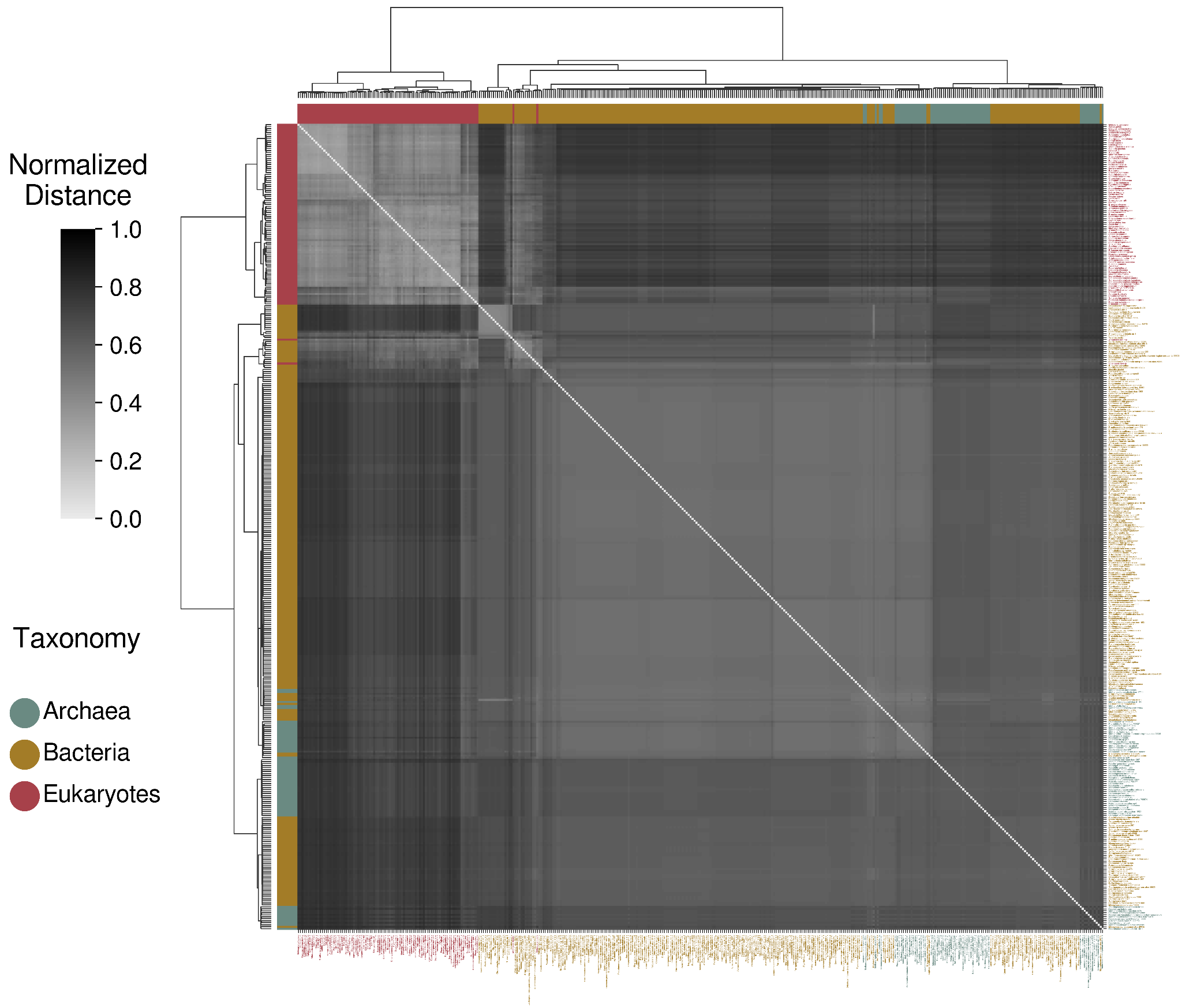


Figure S2.18B: Phylogenetic clustergram derived from the comparison of “DNA recombination” semantic networks, excluding terms associated with the obtained PN-related semantic groups.


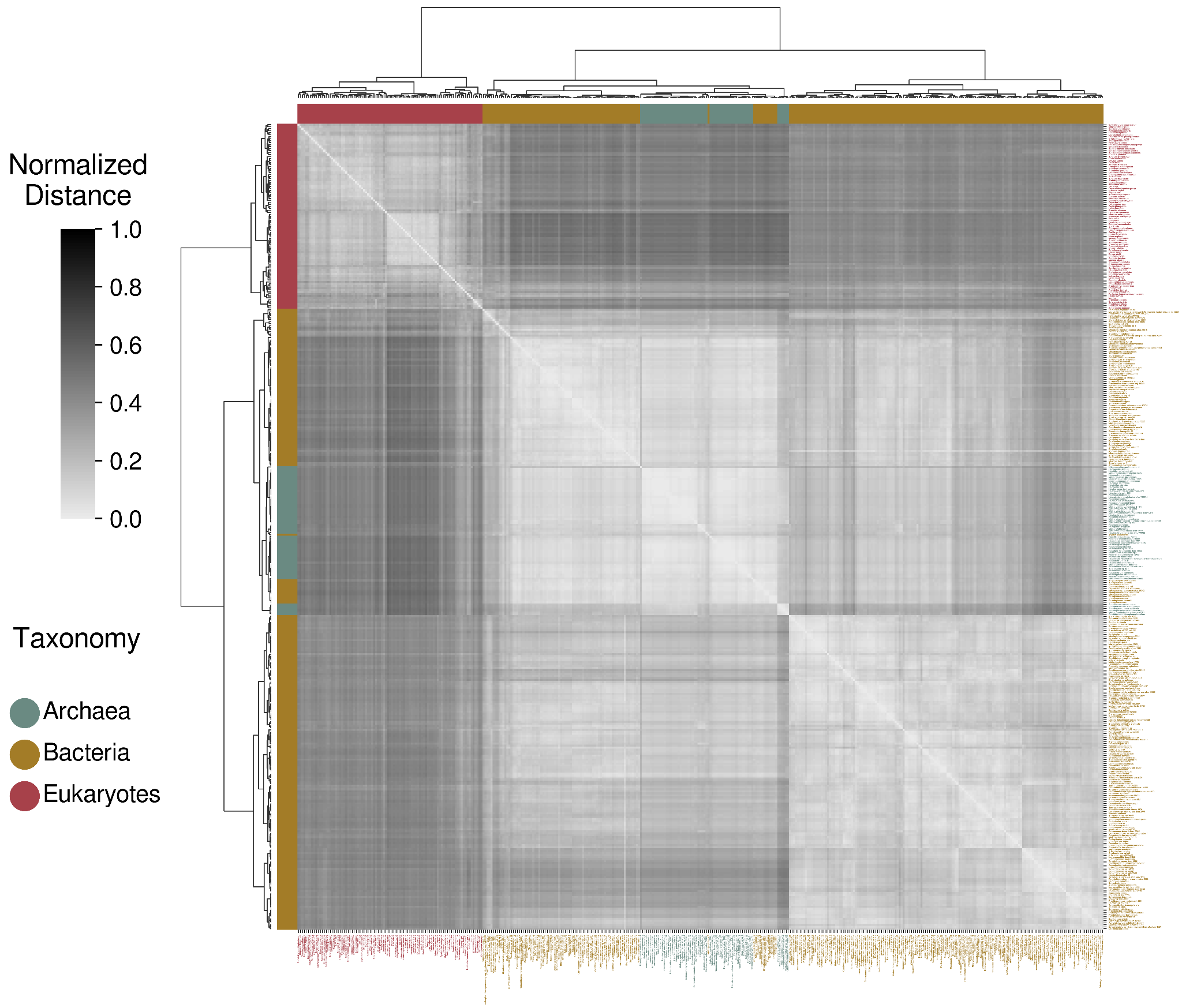


Figure S2.19A: Phylogenetic clustergram derived from the comparison of “DNA repair” semantic networks.


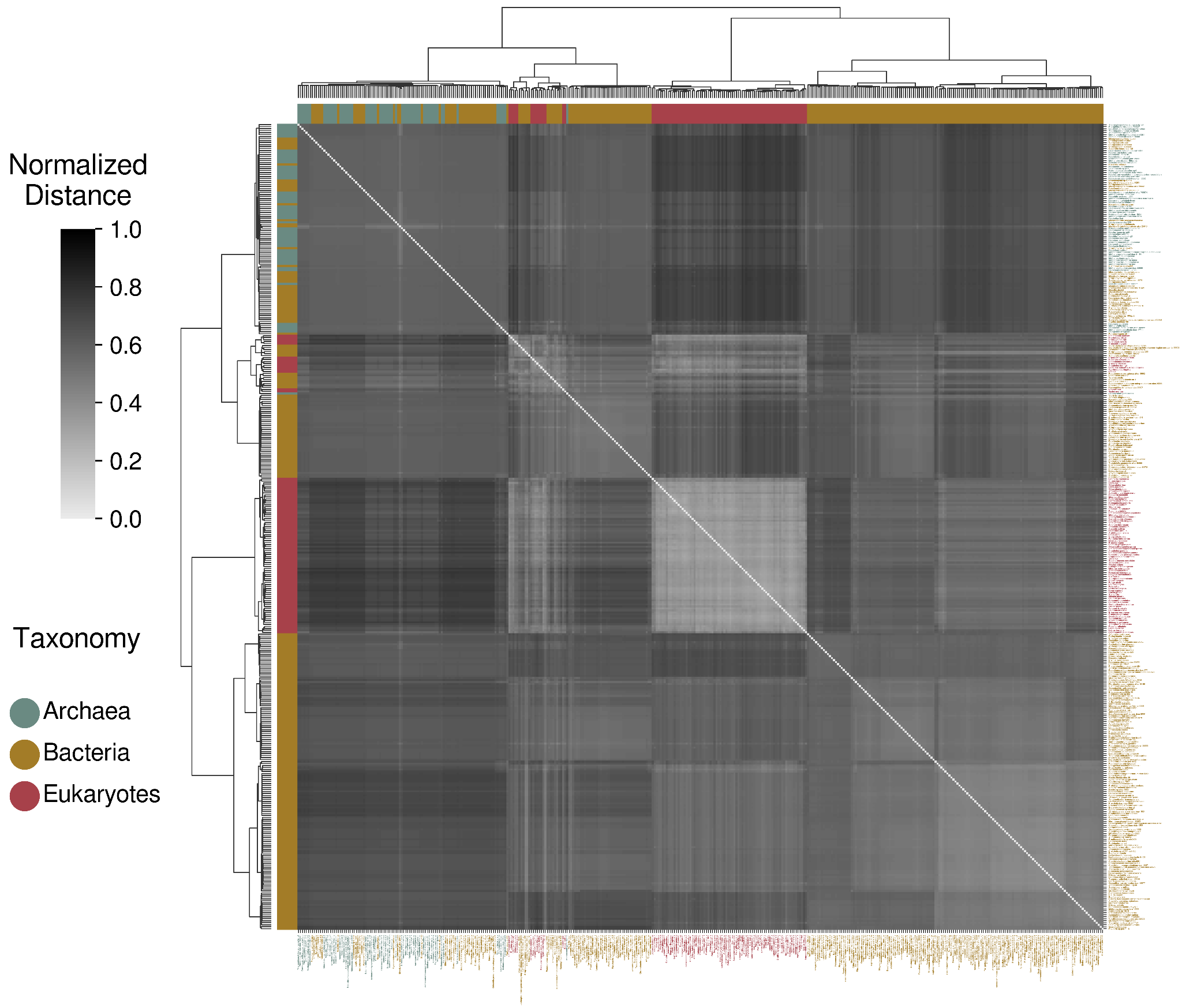


Figure S2.19B: Phylogenetic clustergram derived from the comparison of “DNA repair” semantic networks, excluding terms associated with the obtained PN-related semantic groups.


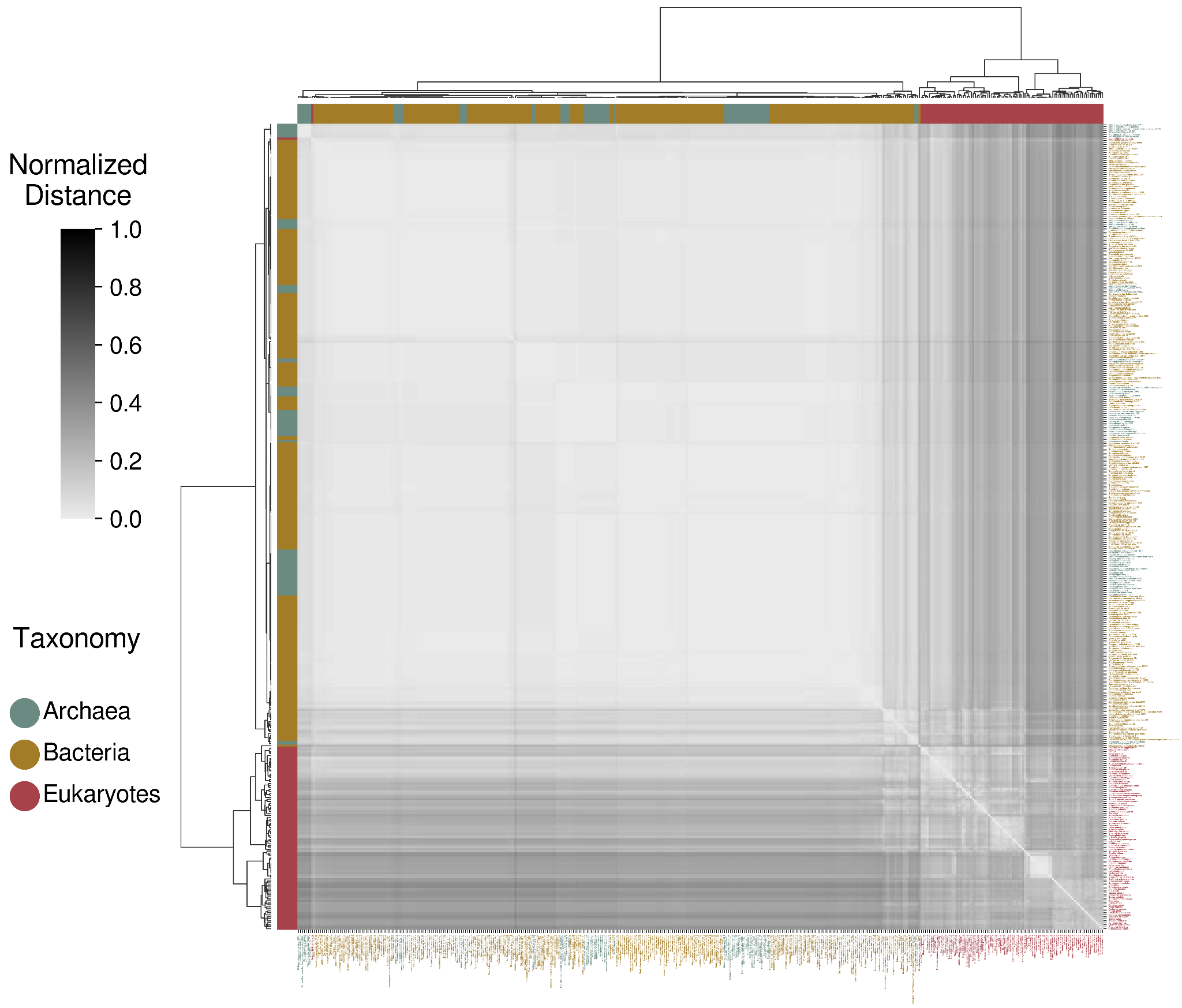


Figure S2.20A: Phylogenetic clustergram derived from the comparison of “glycolytic process” semantic networks.


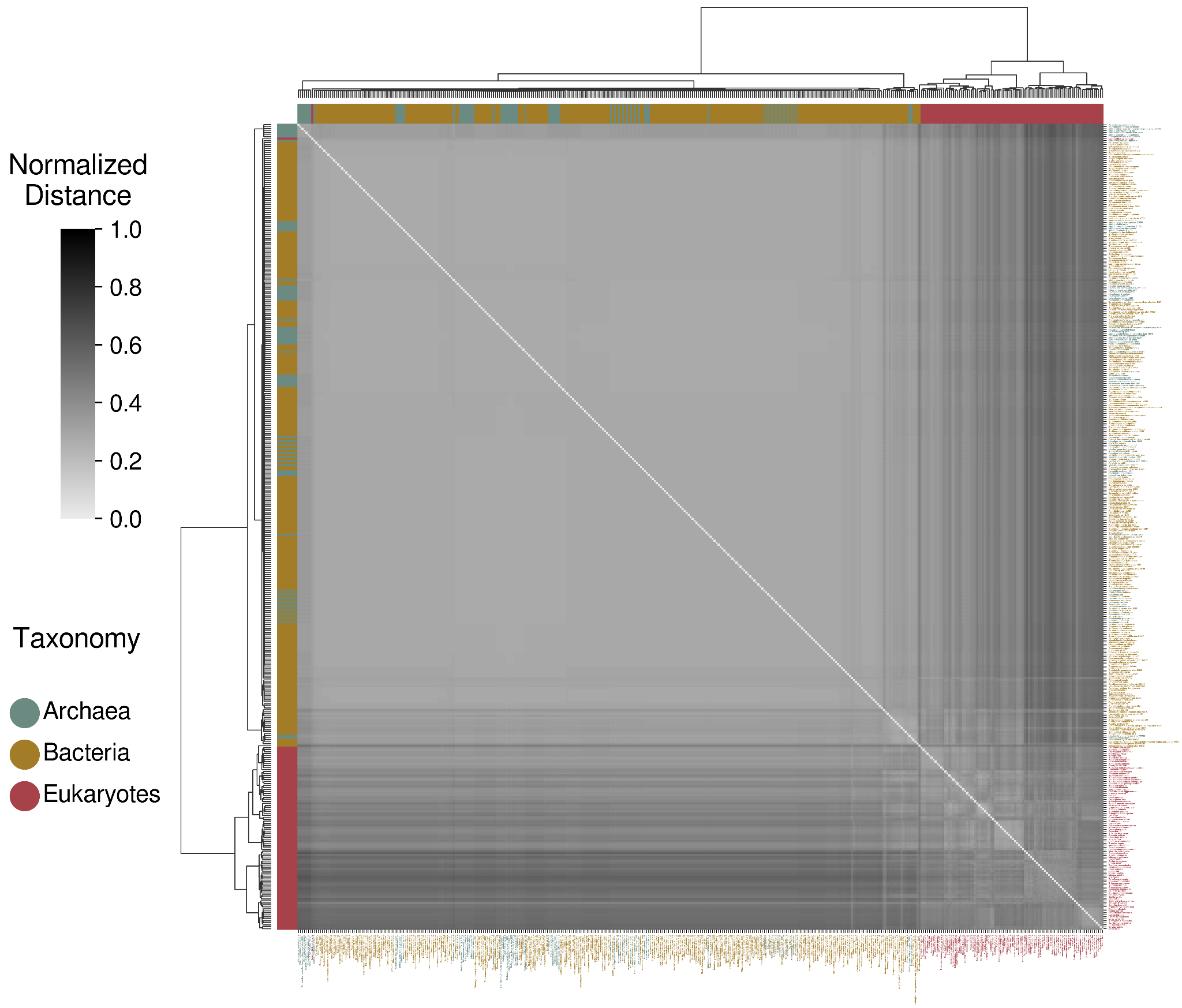


Figure S2.20B: Phylogenetic clustergram derived from the comparison of “glycolytic process” semantic networks, excluding terms associated with the obtained PN-related semantic groups.


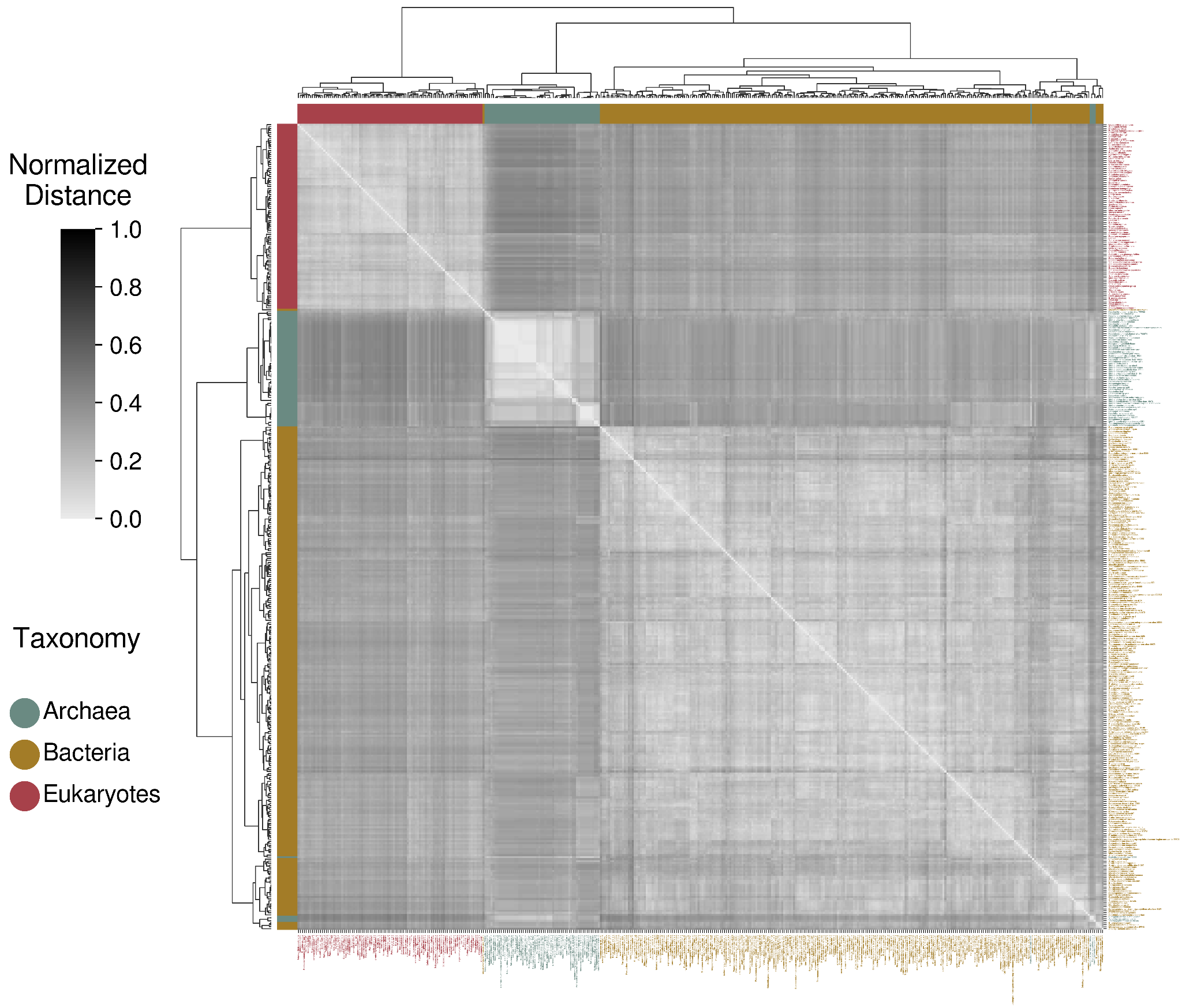


Figure S2.21A: Phylogenetic clustergram derived from the comparison of “lipid metabolic process” semantic networks.


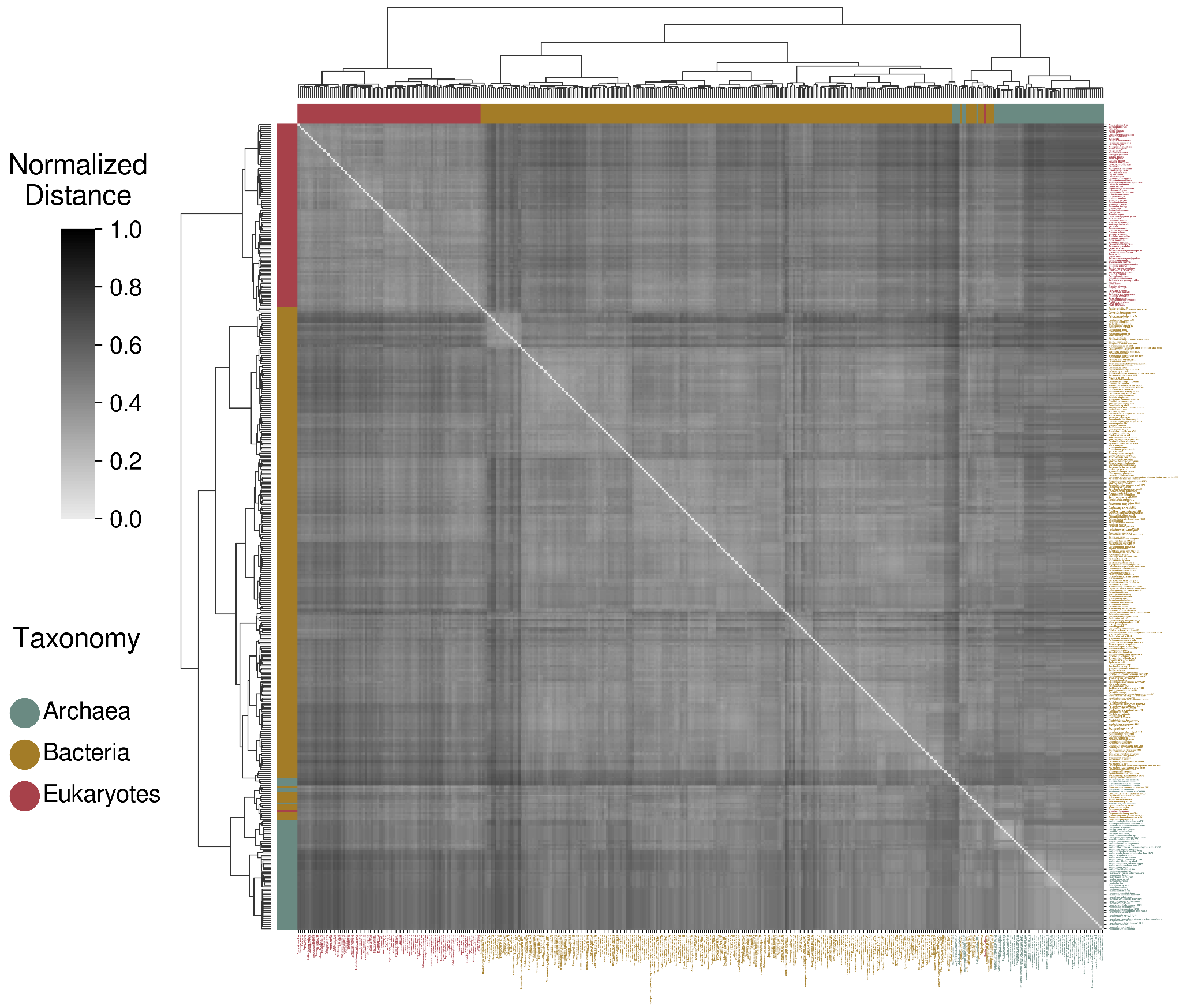


Figure S2.21B: Phylogenetic clustergram derived from the comparison of “lipid metabolic process” semantic networks, excluding terms associated with the obtained PN-related semantic groups.


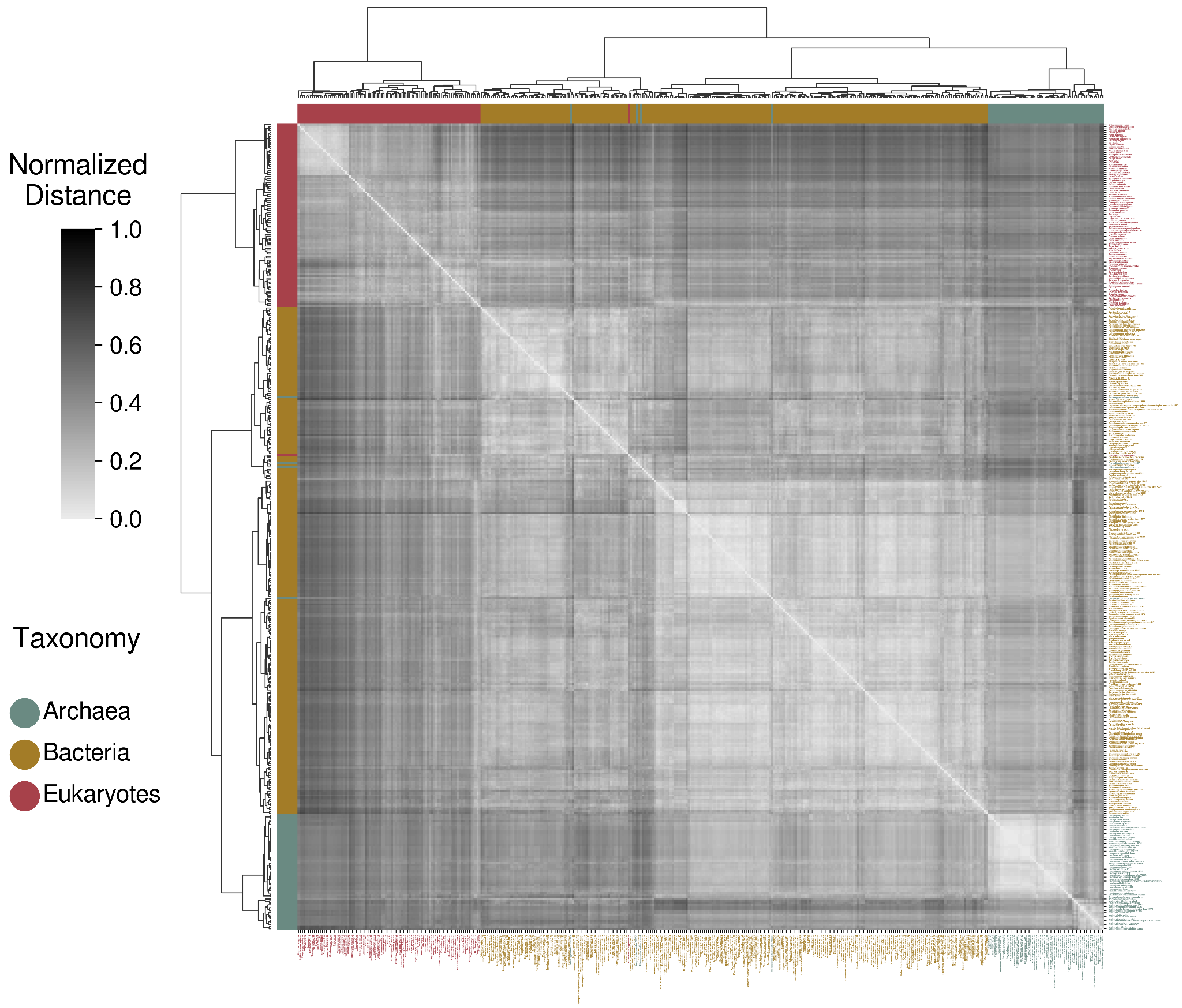


Figure S2.22A: Phylogenetic clustergram derived from the comparison of “methylation” semantic networks.


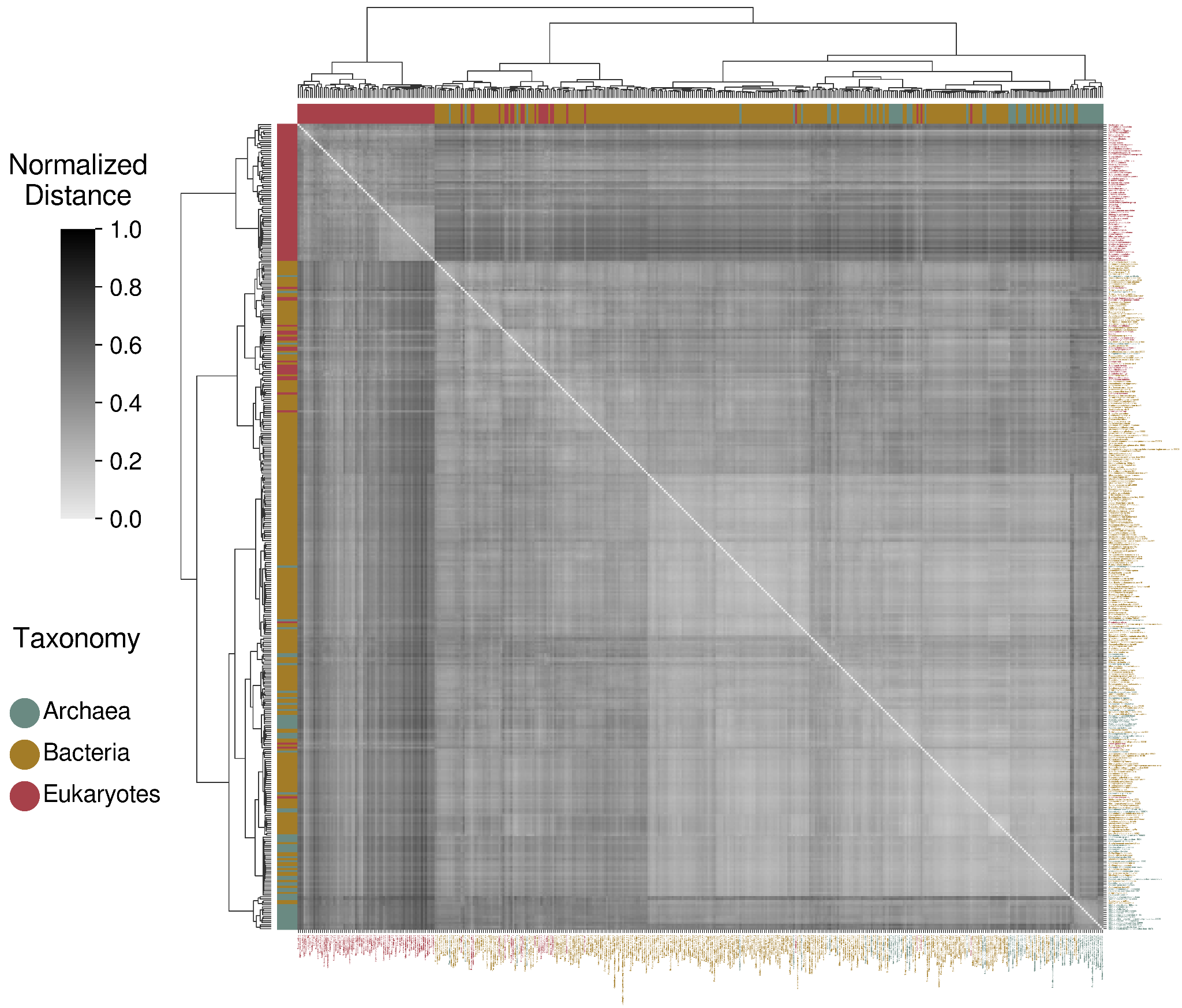


Figure S2.22B: Phylogenetic clustergram derived from the comparison of “methylation” semantic networks, excluding terms associated with the obtained PN-related semantic groups.


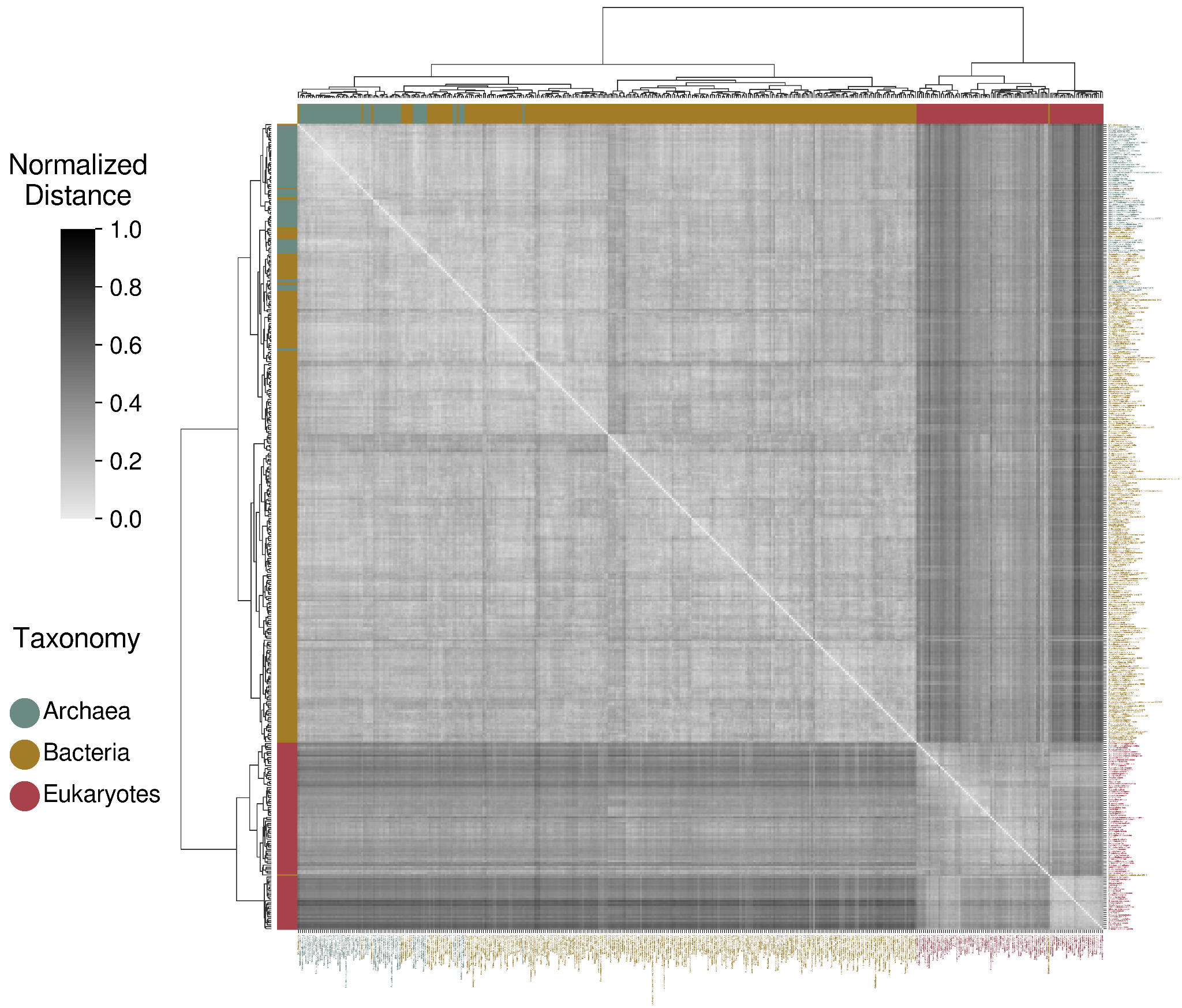


Figure S2.23A: Phylogenetic clustergram derived from the comparison of “phosphorylation” semantic networks.


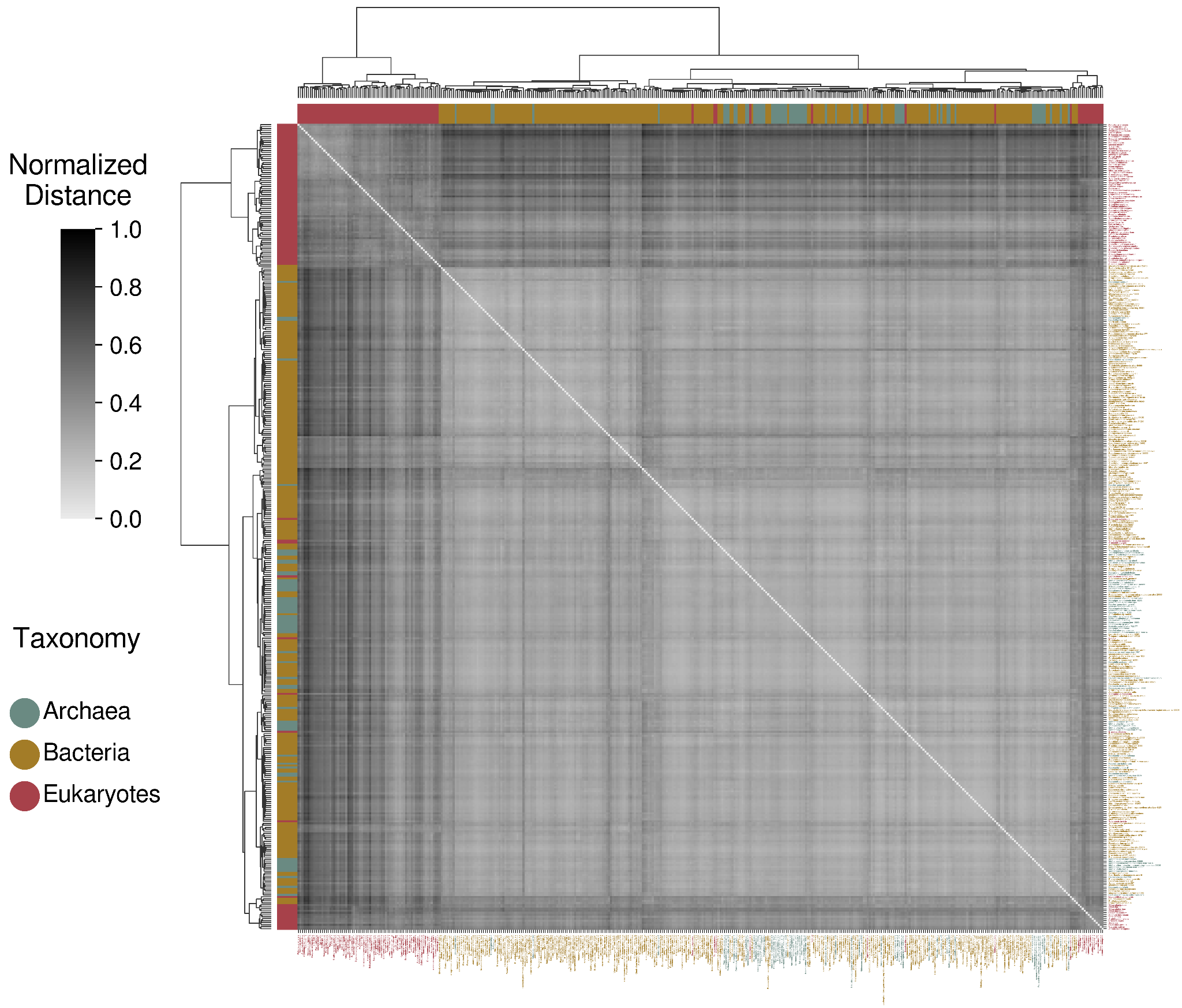


Figure S2.23B: Phylogenetic clustergram derived from the comparison of “phosphorylation” semantic networks, excluding terms associated with the obtained PN-related semantic groups.


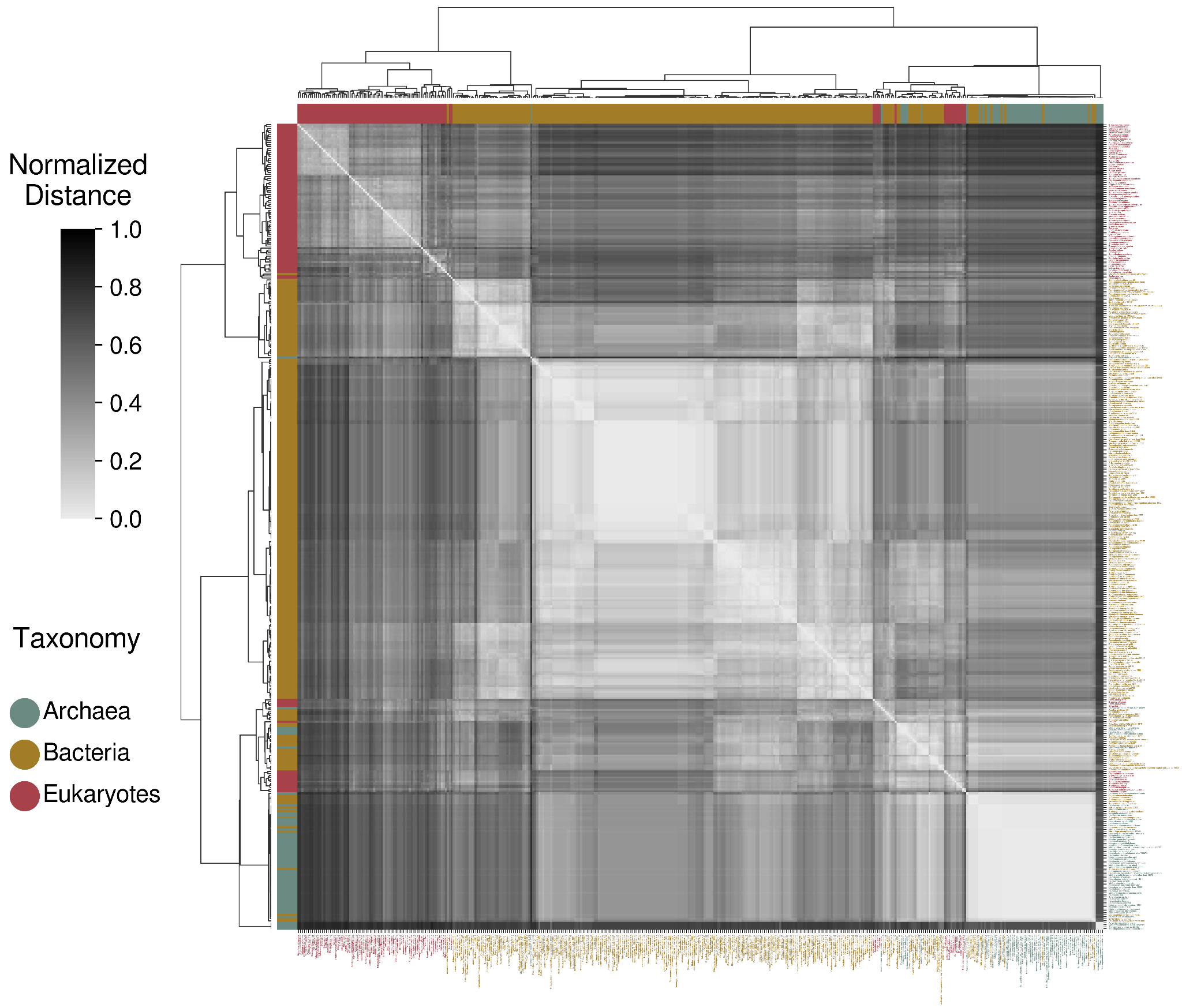


Figure S2.24A: Phylogenetic clustergram derived from the comparison of “protein folding” semantic networks.


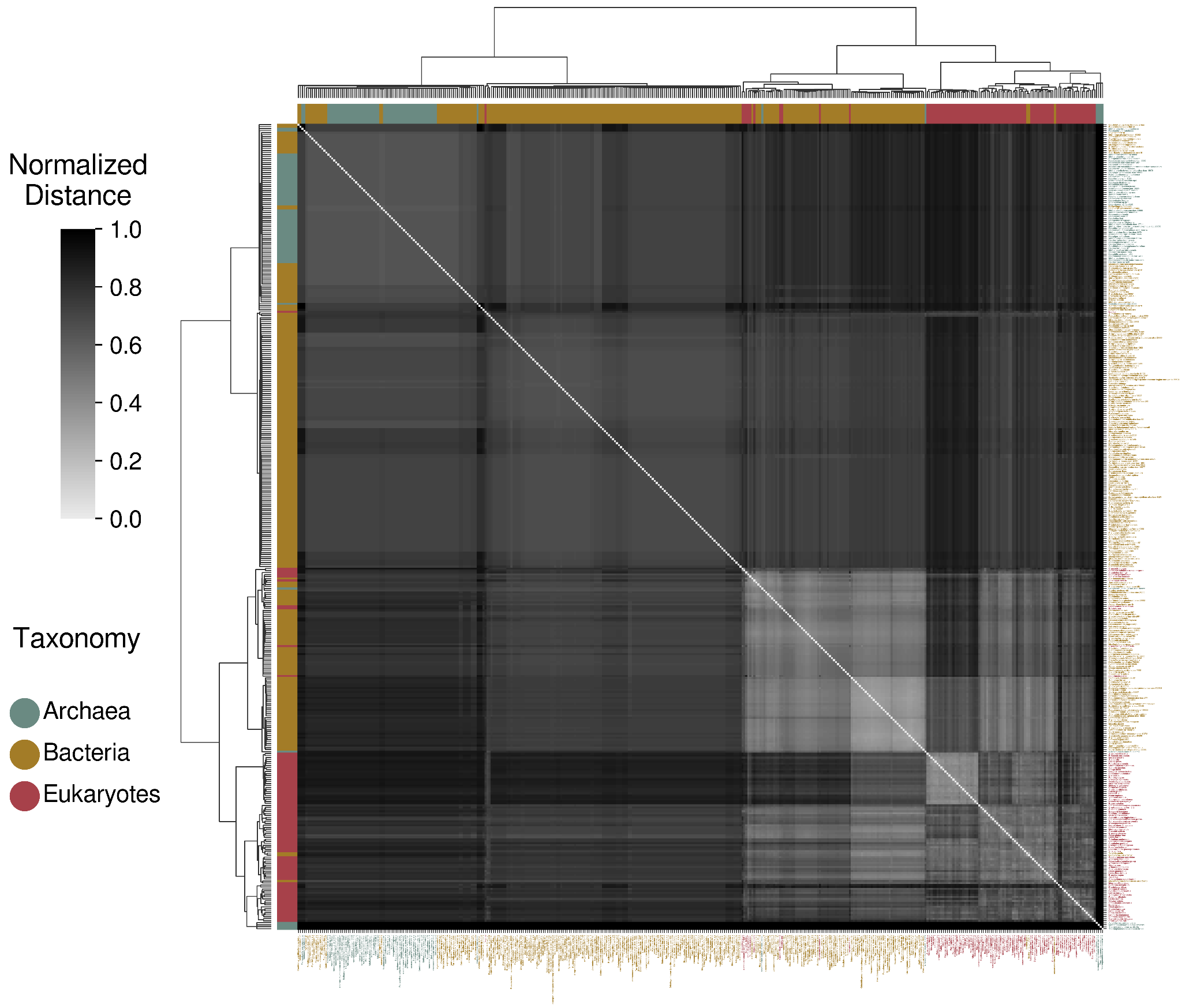


Figure S2.24B: Phylogenetic clustergram derived from the comparison of “protein folding” semantic networks, excluding terms associated with the obtained PN-related semantic groups.


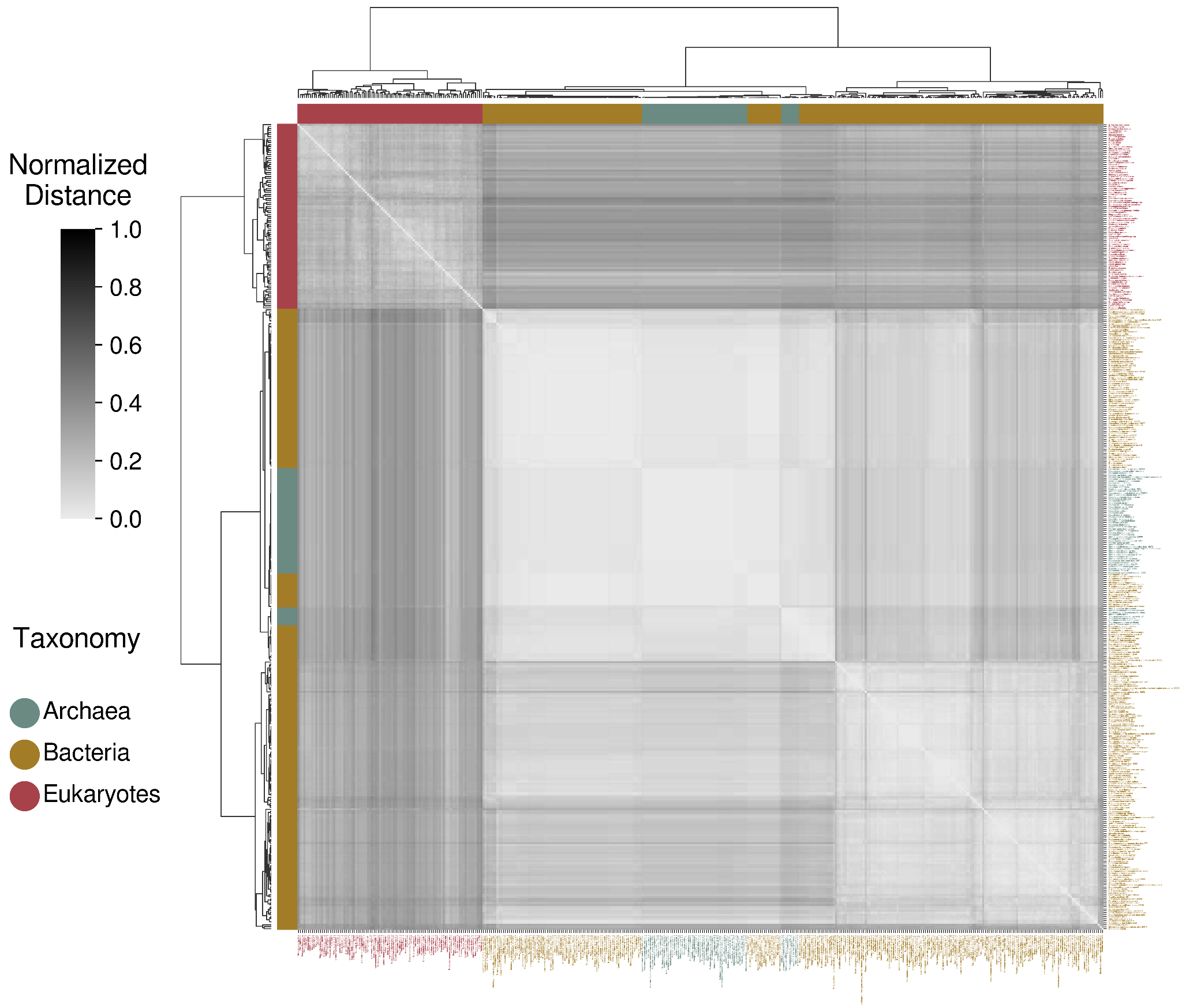


Figure S2.25A: Phylogenetic clustergram derived from the comparison of “protein localization” semantic networks.


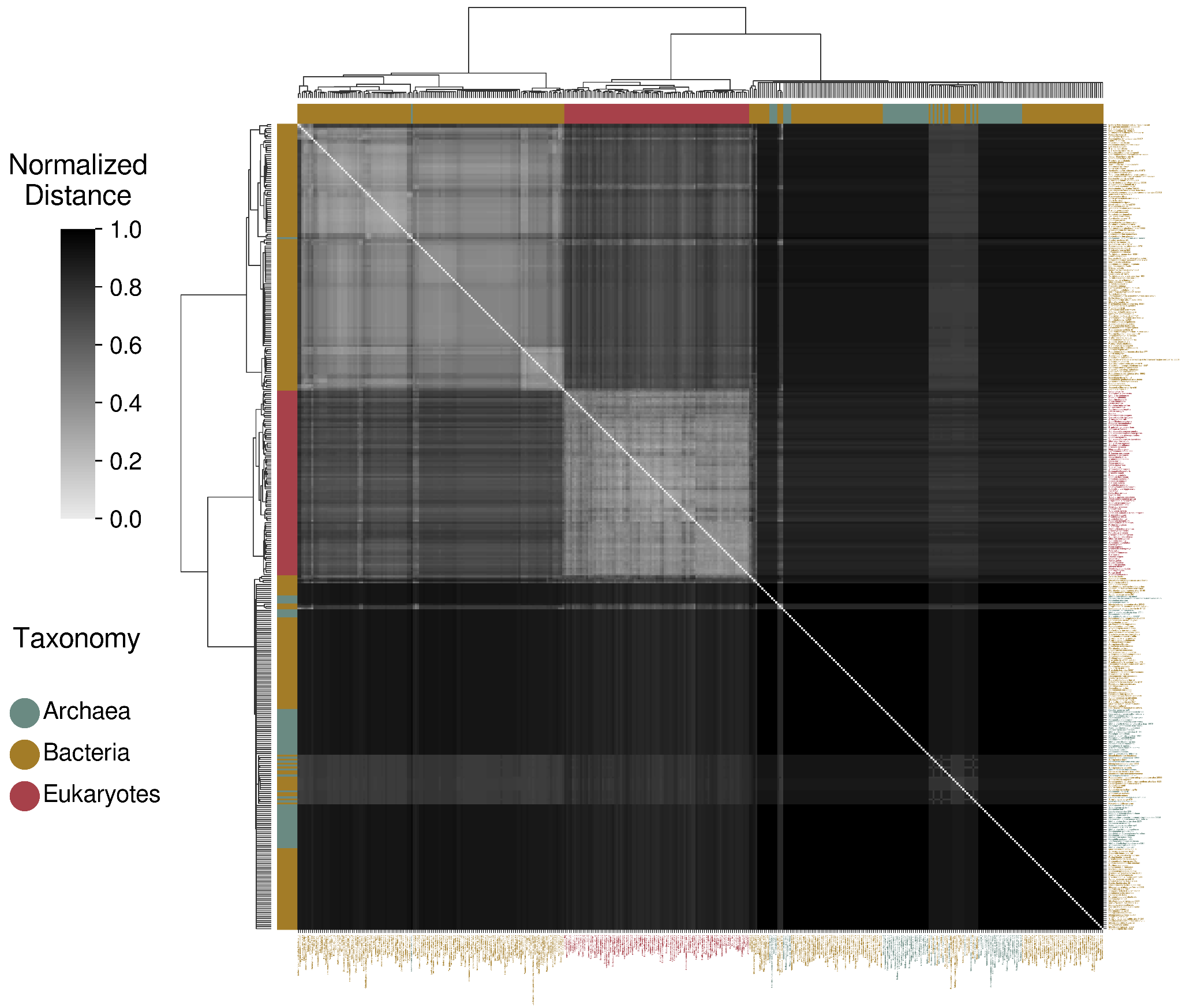


Figure S2.25B: Phylogenetic clustergram derived from the comparison of “protein localization” semantic networks, excluding terms associated with the obtained PN-related semantic groups.


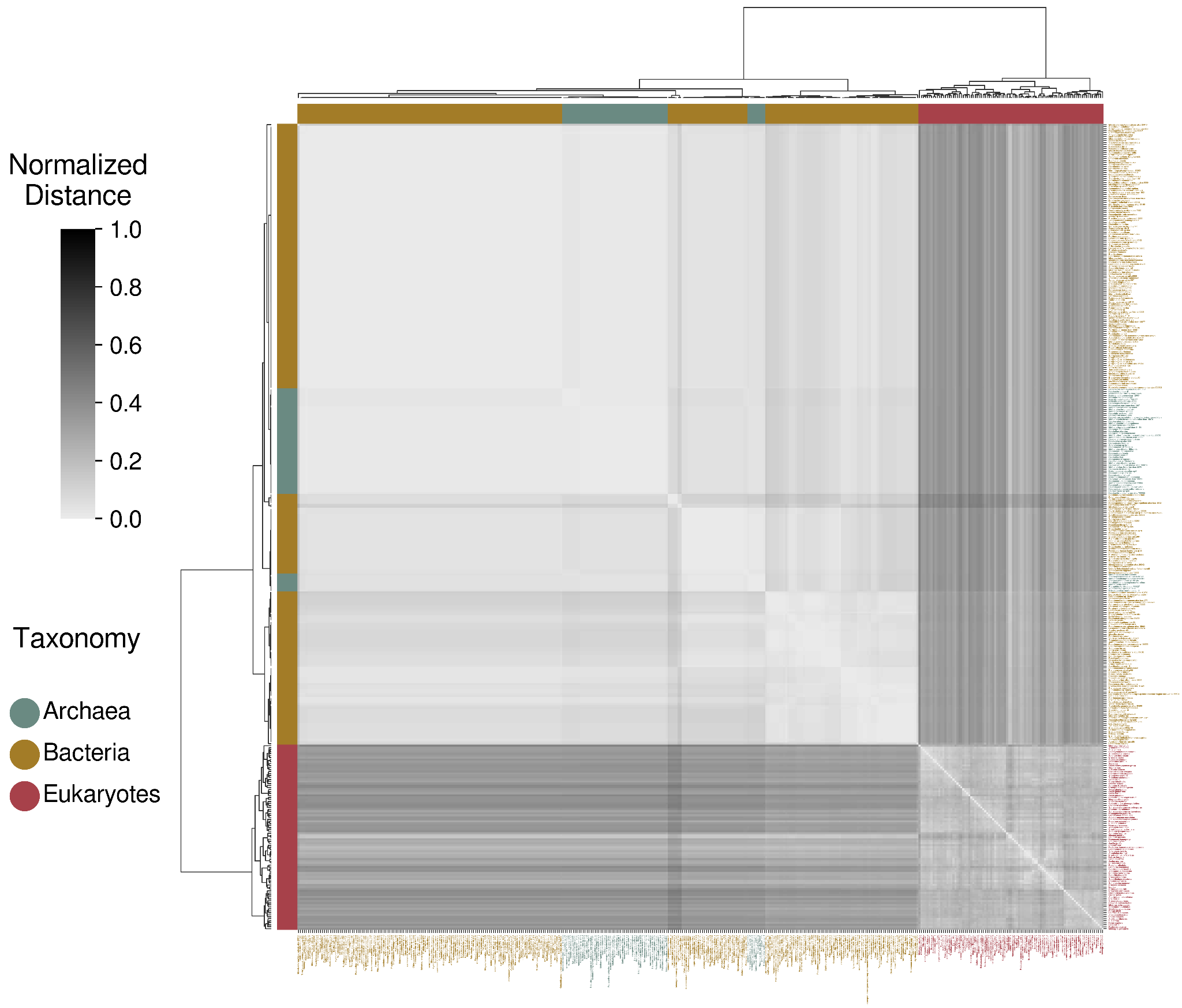


Figure S2.26A: Phylogenetic clustergram derived from the comparison of “protein targeting” semantic networks.


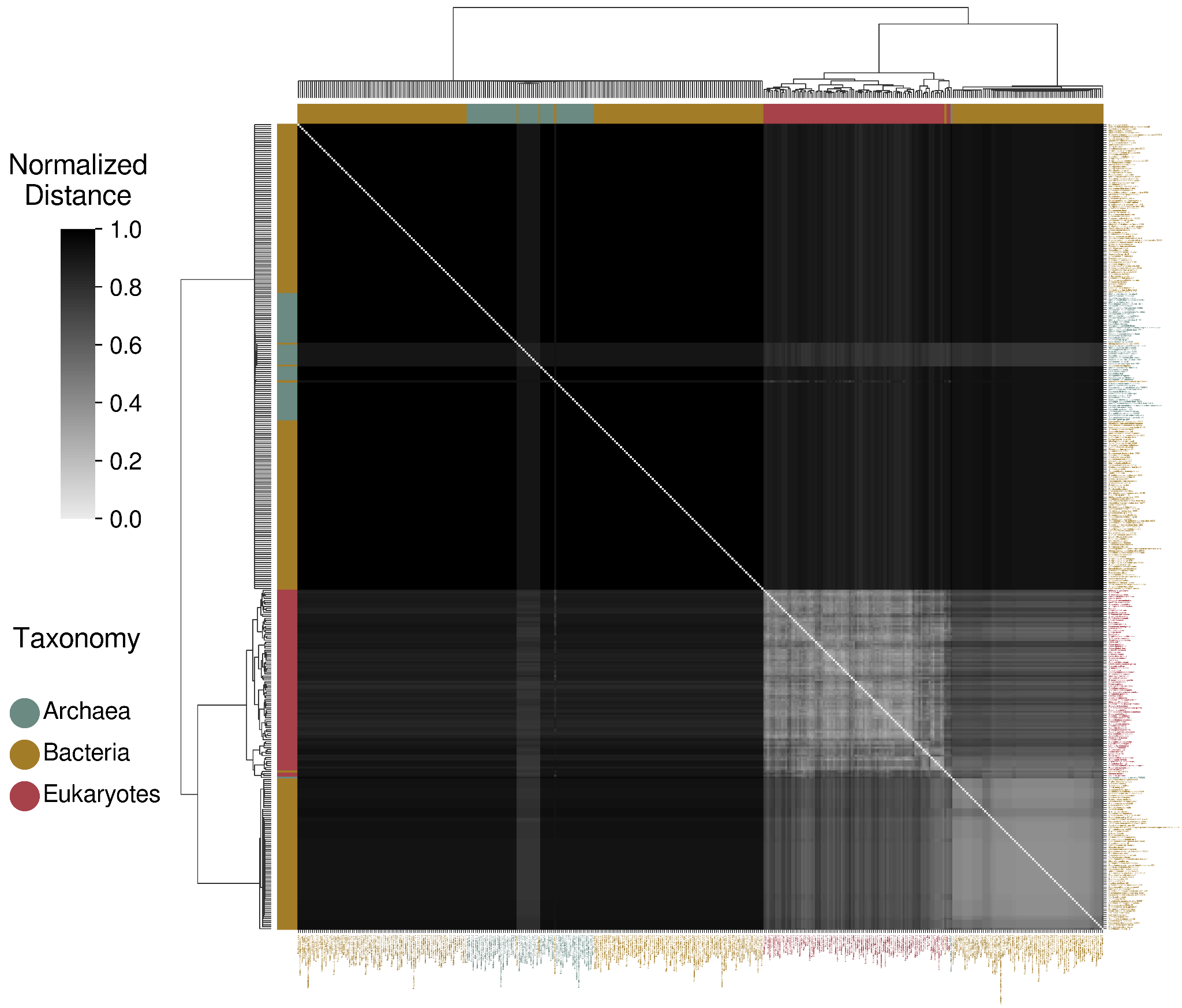


Figure S2.26B: Phylogenetic clustergram derived from the comparison of “protein targeting” semantic networks, excluding terms associated with the obtained PN-related semantic groups.


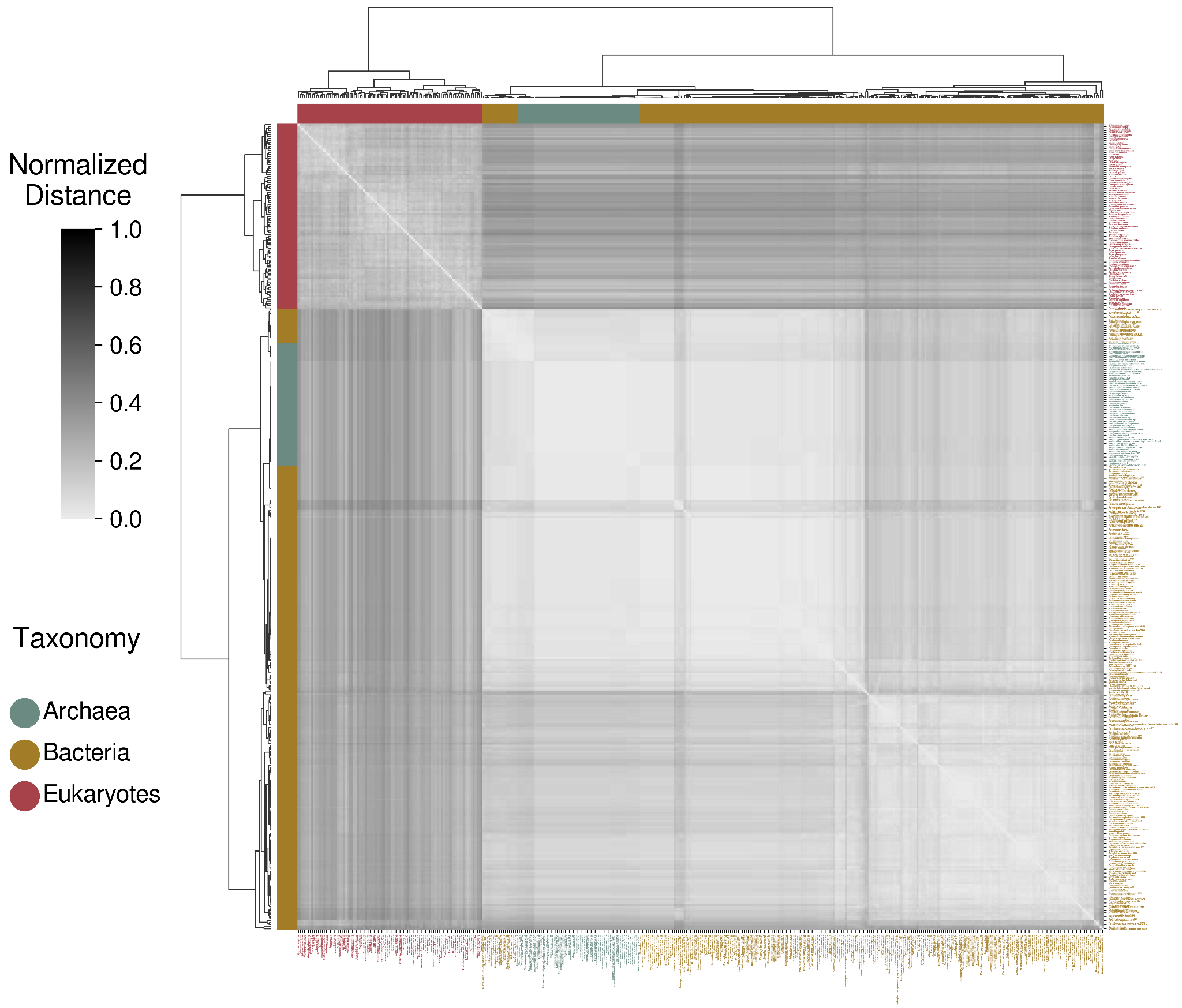


Figure S2.27A: Phylogenetic clustergram derived from the comparison of “protein transport” semantic networks.


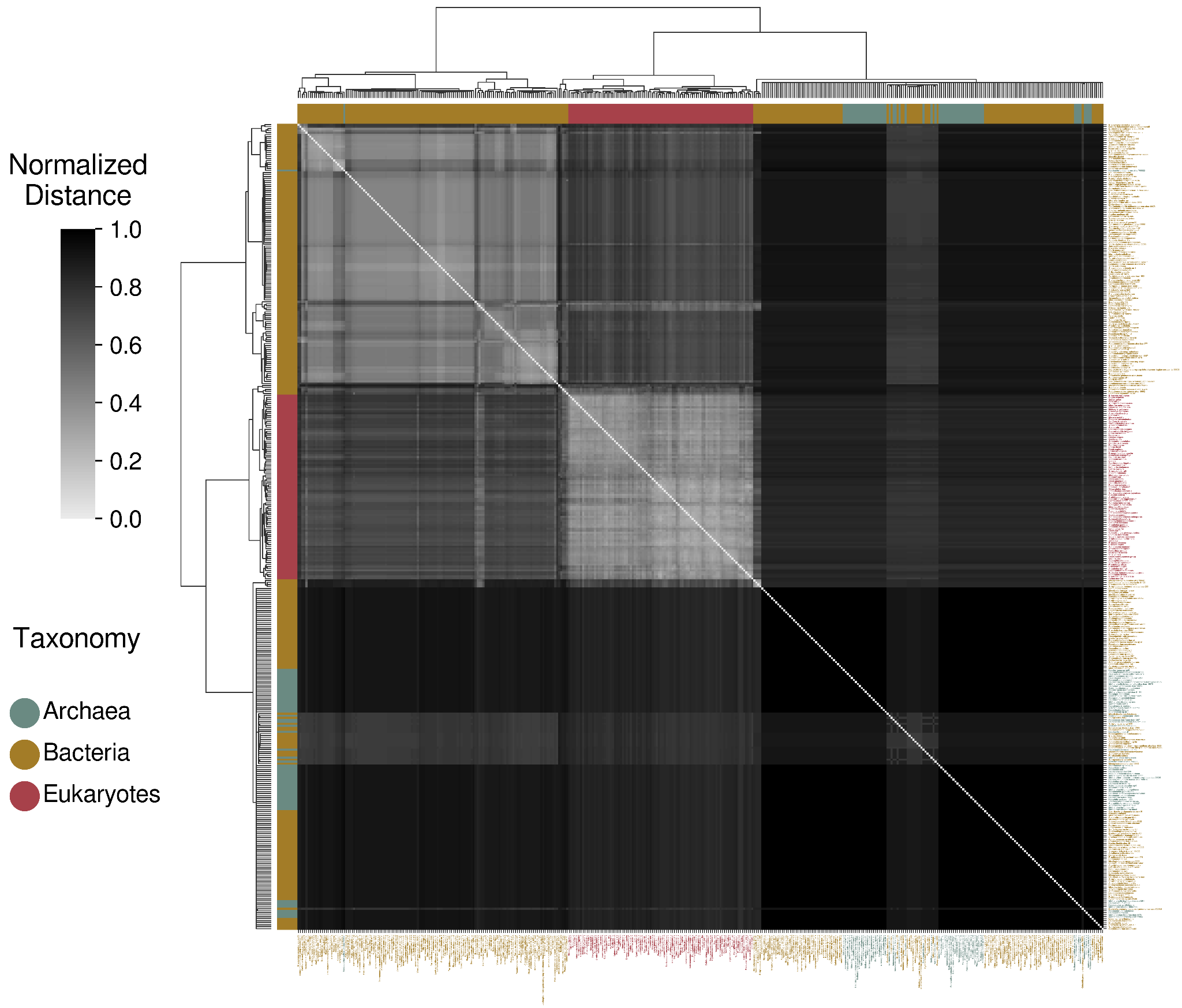


Figure S2.27B: Phylogenetic clustergram derived from the comparison of “protein transport” semantic networks, excluding terms associated with the obtained PN-related semantic groups.


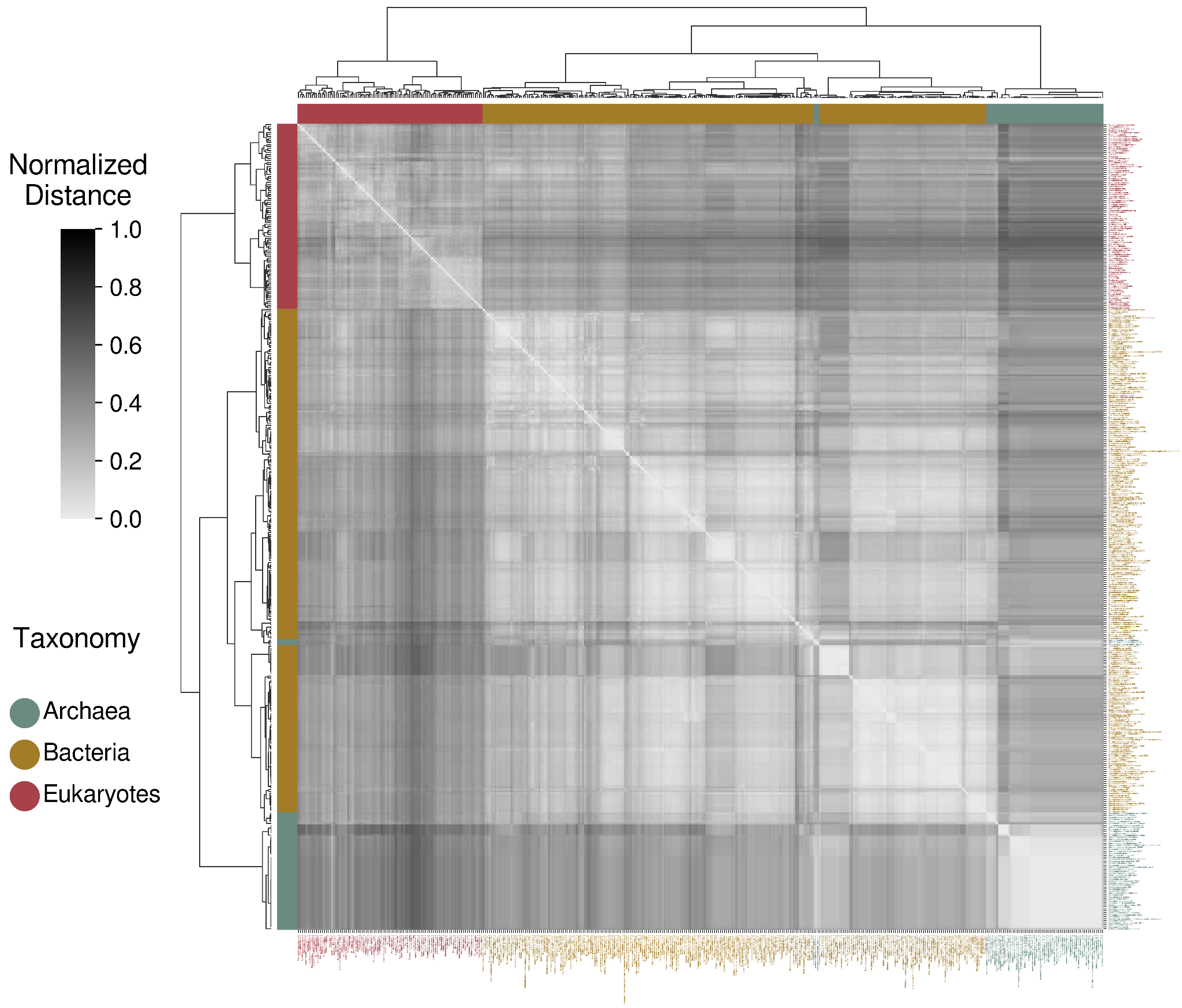


Figure S2.28A: Phylogenetic clustergram derived from the comparison of “regulation of DNA-templated transcription” semantic networks.


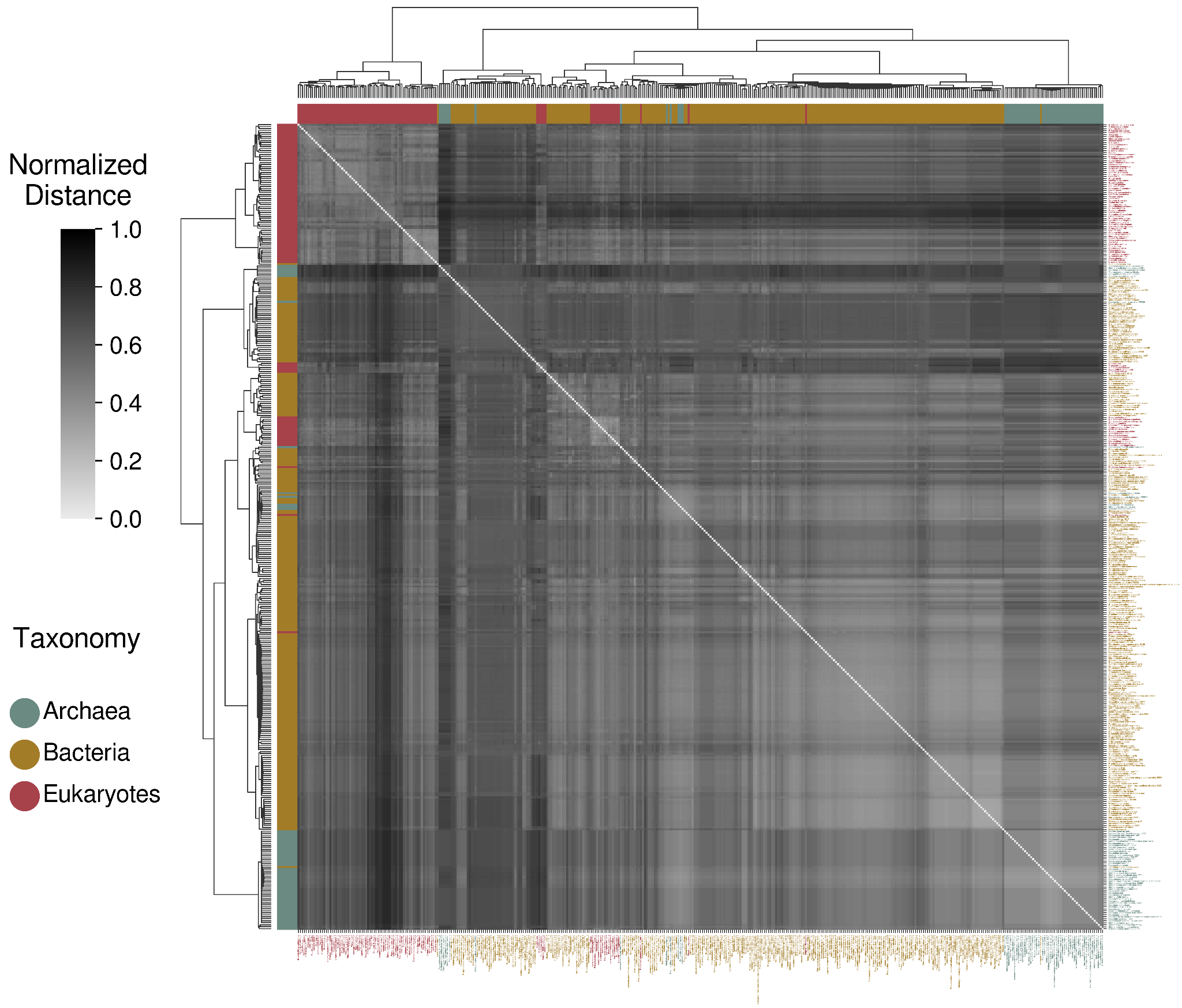


Figure S2.28B: Phylogenetic clustergram derived from the comparison of “regulation of DNA-templated transcription” semantic networks, excluding terms associated with the obtained PN-related semantic groups.


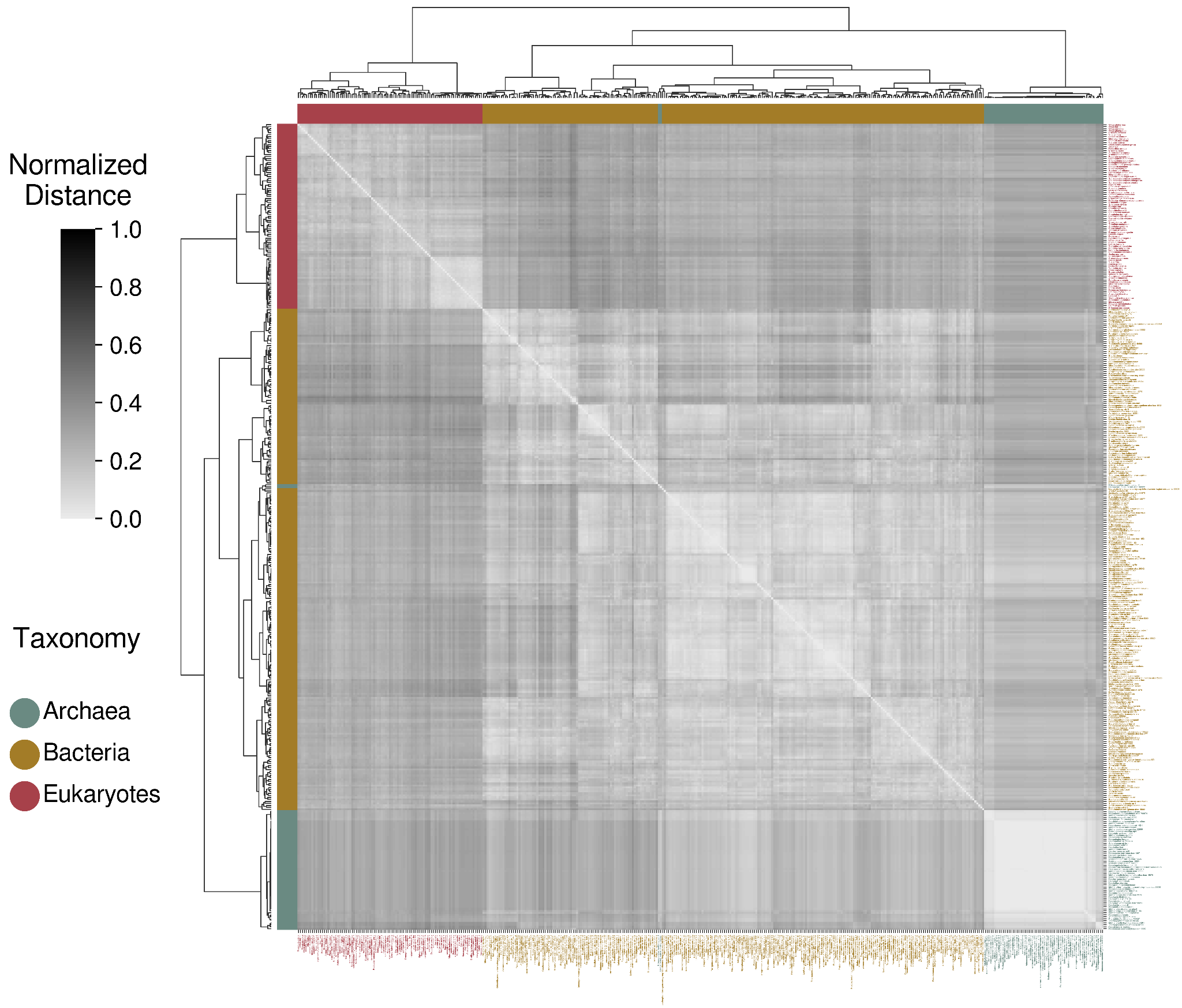


Figure S2.29A: Phylogenetic clustergram derived from the comparison of “regulation of macromolecule metabolic process” semantic networks.


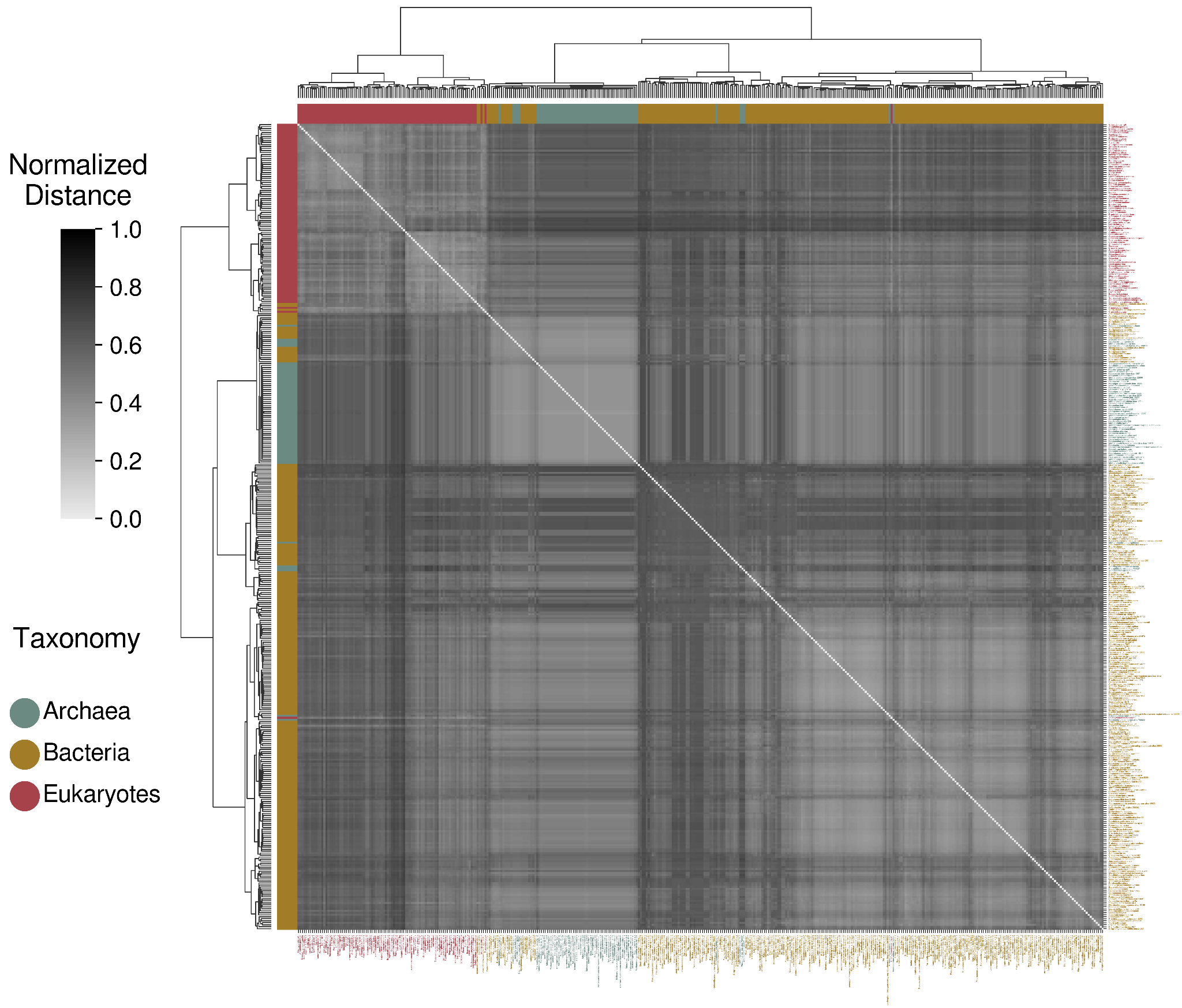


Figure S2.29B: Phylogenetic clustergram derived from the comparison of “regulation of macromolecule metabolic process” semantic networks, excluding terms associated with the obtained PN-related semantic groups.


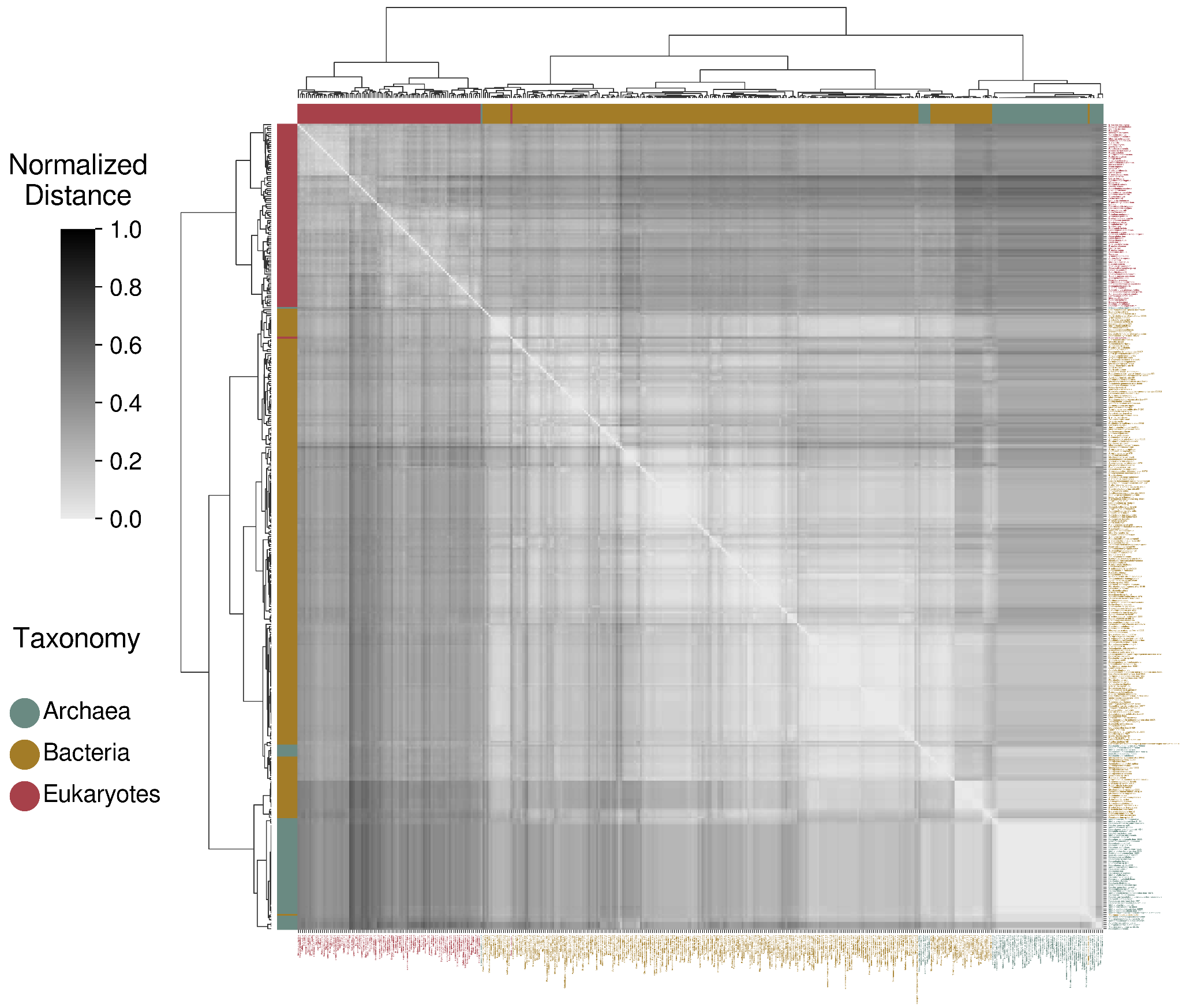


Figure S2.30A: Phylogenetic clustergram derived from the comparison of “regulation of RNA metabolic process” semantic networks.


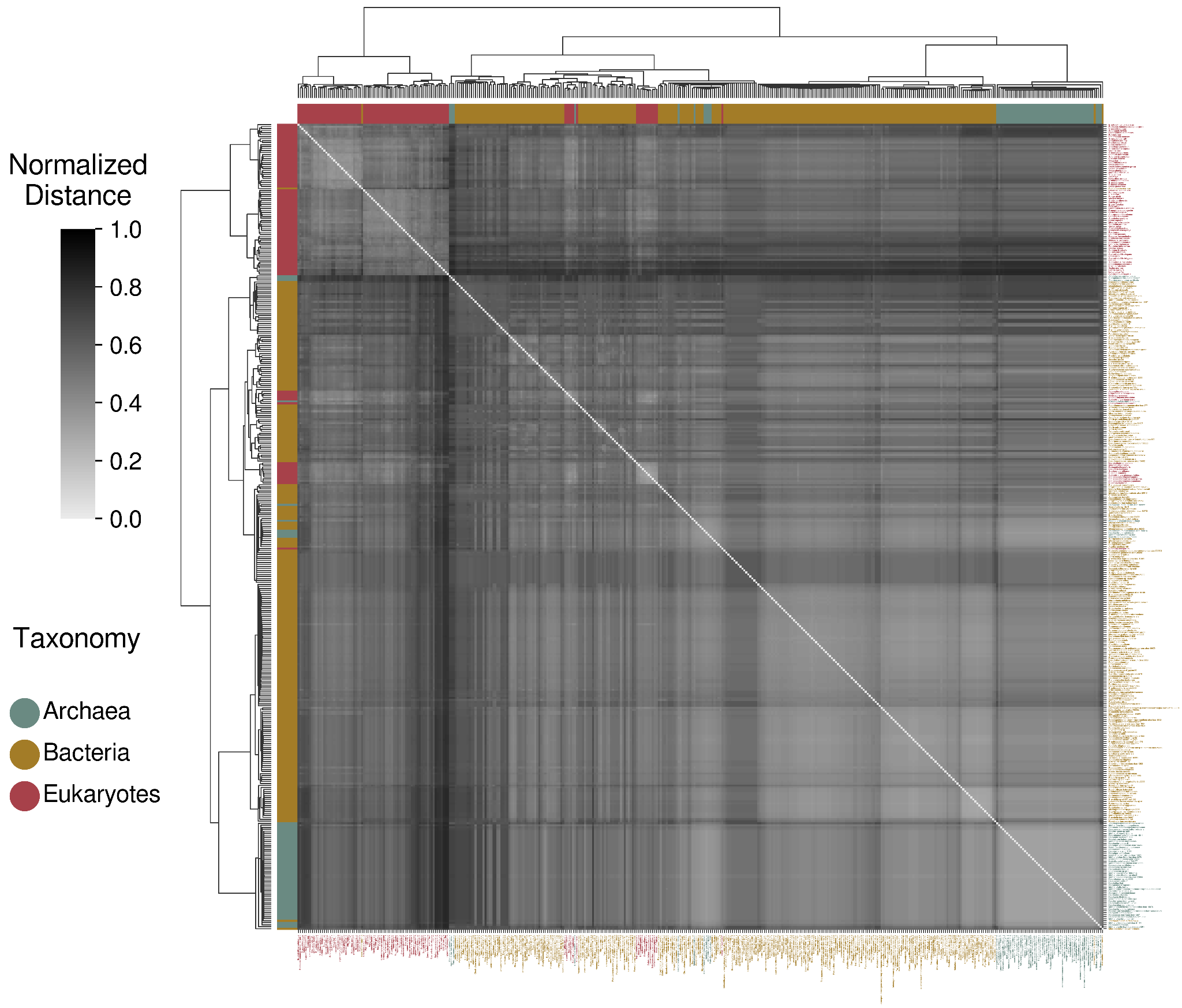


Figure S2.30B: Phylogenetic clustergram derived from the comparison of “regulation of RNA metabolic process” semantic networks, excluding terms associated with the obtained PN-related semantic groups.


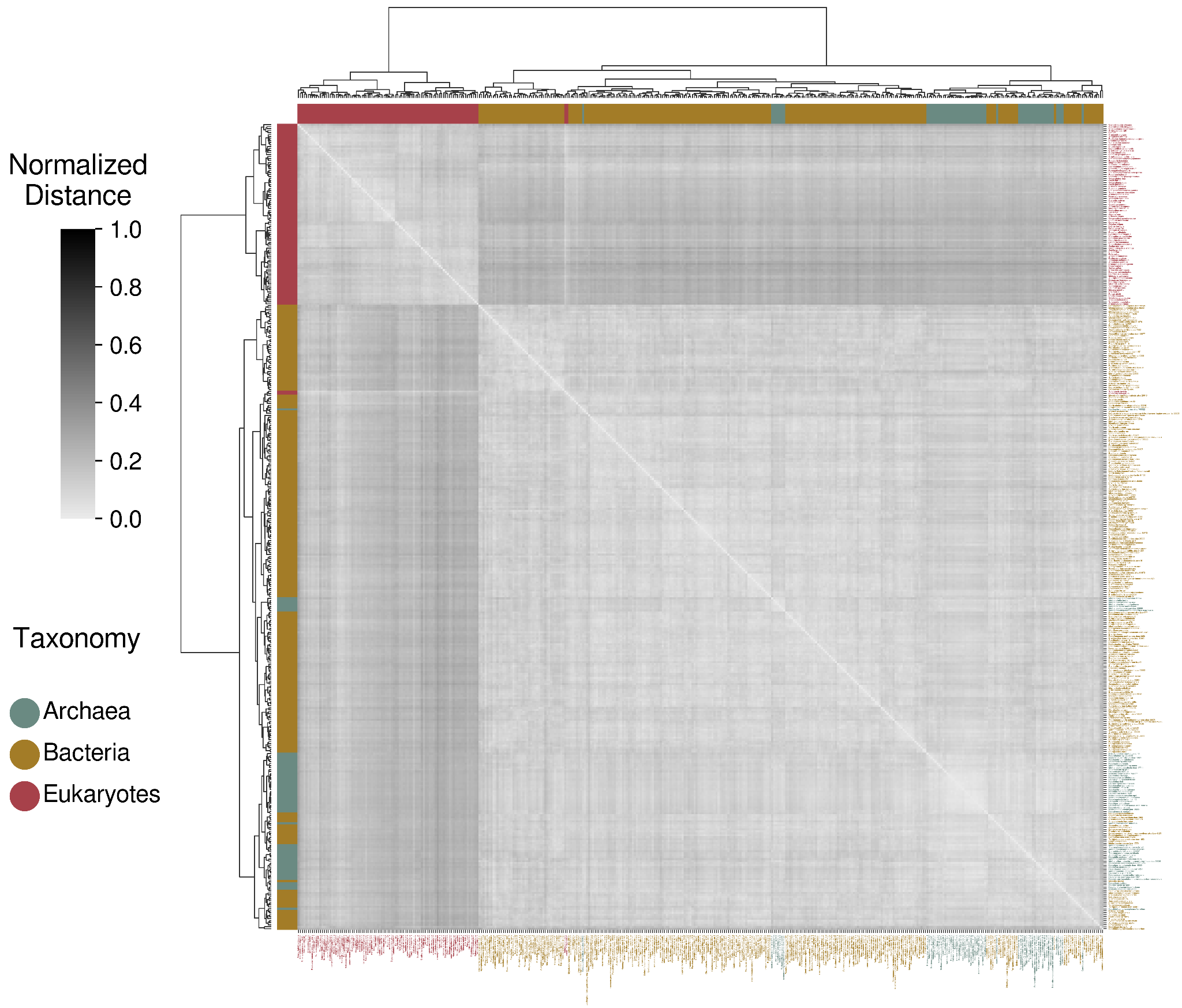


Figure S2.31A: Phylogenetic clustergram derived from the comparison of “ribonucleotide biosynthetic process” semantic networks.


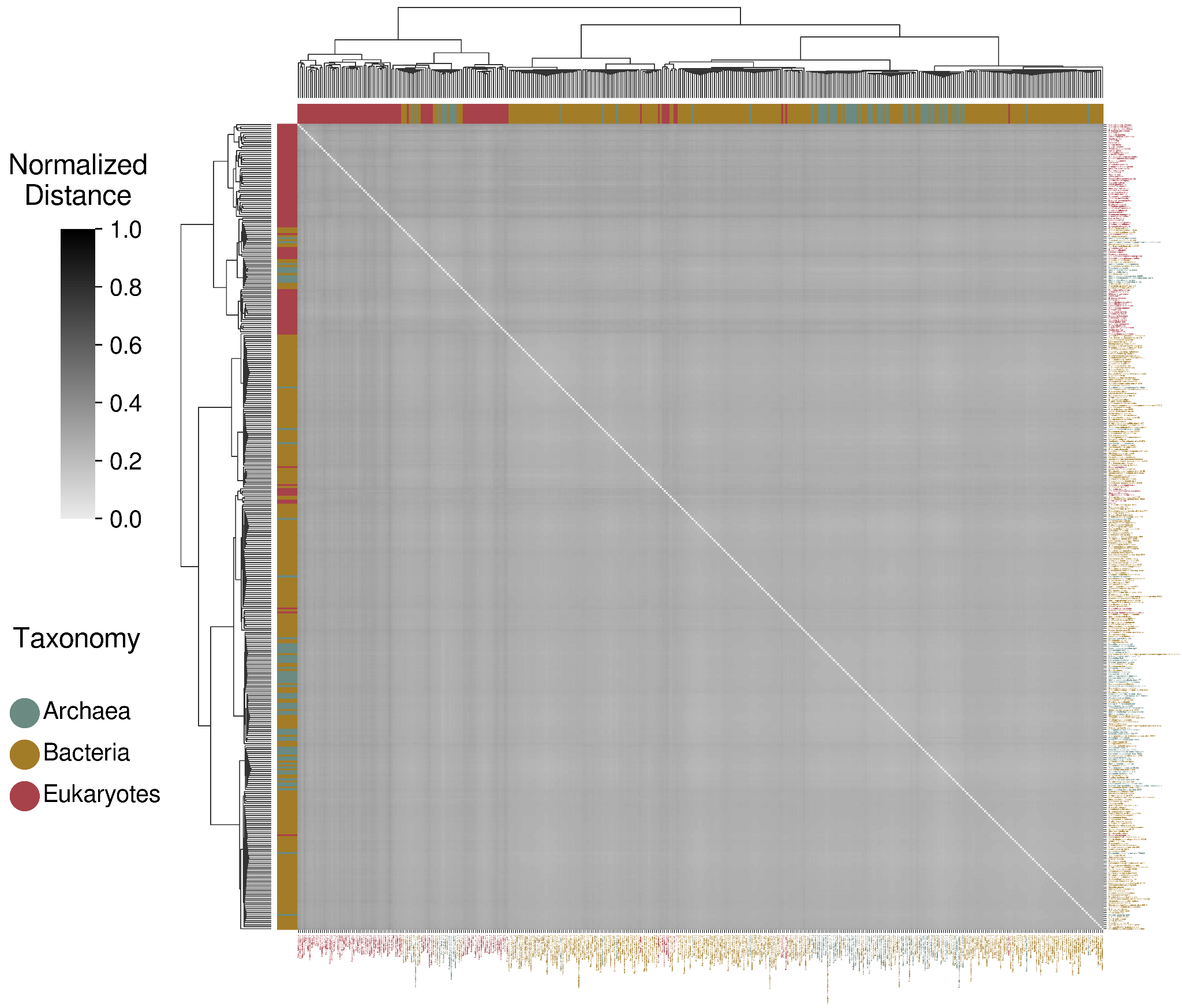


Figure S2.31B: Phylogenetic clustergram derived from the comparison of “ribonucleotide biosynthetic process” semantic networks, excluding terms associated with the obtained PN-related semantic groups.


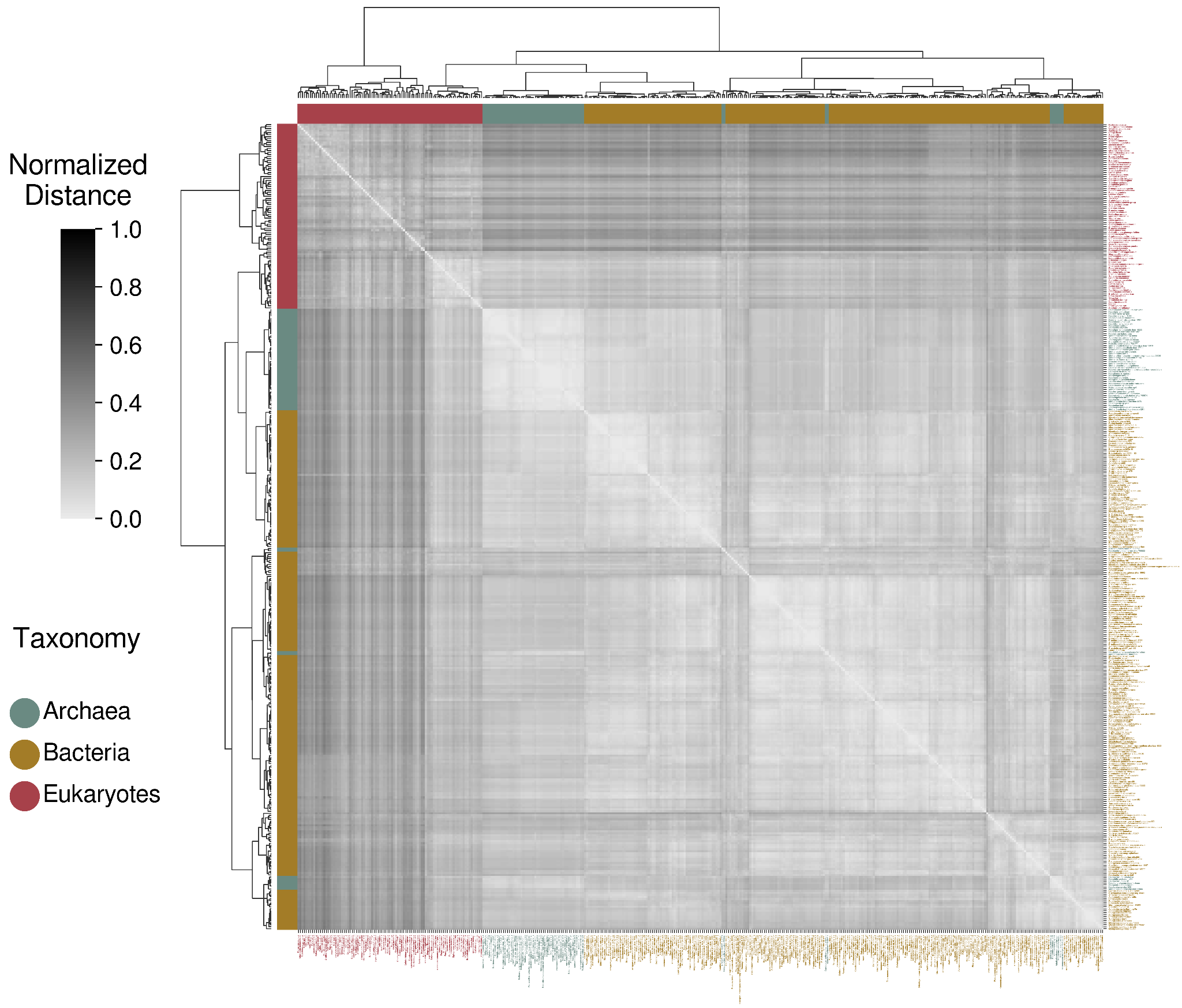


Figure S2.32A: Phylogenetic clustergram derived from the comparison of “tRNA processing” semantic networks.


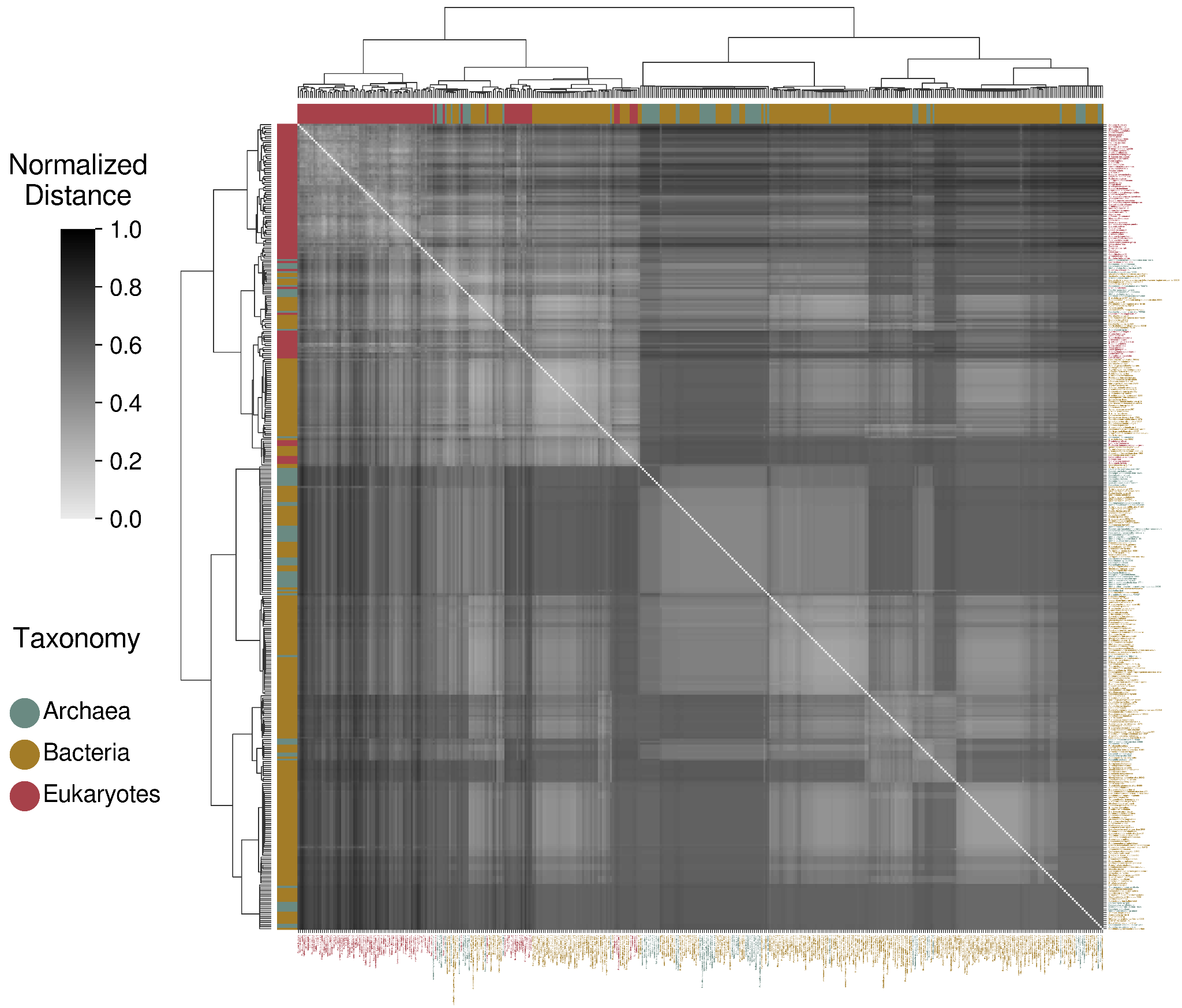


Figure S2.32B: Phylogenetic clustergram derived from the comparison of “tRNA processing” semantic networks, excluding terms associated with the obtained PN-related semantic groups.
